# 53BP1 Integrates DNA Repair and p53-Dependent Cell Fate Decisions via Distinct Mechanisms

**DOI:** 10.1016/j.molcel.2016.08.002

**Published:** 2016-10-06

**Authors:** Raquel Cuella-Martin, Catarina Oliveira, Helen E. Lockstone, Suzanne Snellenberg, Natalia Grolmusova, J. Ross Chapman

**Affiliations:** 1 Chromatin and Genome Integrity Laboratory, Wellcome Trust Centre for Human Genetics, University of Oxford, Oxford OX3 7BN, UK; 2 Bioinformatics and Statistical Genetics Core, Wellcome Trust Centre for Human Genetics, University of Oxford, Oxford OX3 7BN, UK

## Abstract

The tumor suppressor protein 53BP1, a pivotal regulator of DNA double-strand break (DSB) repair, was first identified as a p53-interacting protein over two decades ago. However, its direct contributions to p53-dependent cellular activities remain undefined. Here, we reveal that 53BP1 stimulates genome-wide p53-dependent gene transactivation and repression events in response to ionizing radiation (IR) and synthetic p53 activation. 53BP1-dependent p53 modulation requires both auto-oligomerization and tandem-BRCT domain-mediated bivalent interactions with p53 and the ubiquitin-specific protease USP28. Loss of these activities results in inefficient p53-dependent cell-cycle checkpoint and exit responses. Furthermore, we demonstrate 53BP1-USP28 cooperation to be essential for normal p53-promoter element interactions and gene transactivation-associated events, yet dispensable for 53BP1-dependent DSB repair regulation. Collectively, our data provide a mechanistic explanation for 53BP1-p53 cooperation in controlling anti-tumorigenic cell-fate decisions and reveal these activities to be distinct and separable from 53BP1’s regulation of DNA double-strand break repair pathway choice.

## Highlights

• 53BP1 enhances genome-wide p53-dependent transcriptional responses
• 53BP1’s DNA repair and p53-regulatory roles are distinct and separable
• Regulation requires 53BP1 oligomerization and BRCT domain interactions with p53 and USP28
• 53BP1-USP28 complexes function to stimulate p53 DNA-binding activity

## Graphical Abstract

**Figure:**
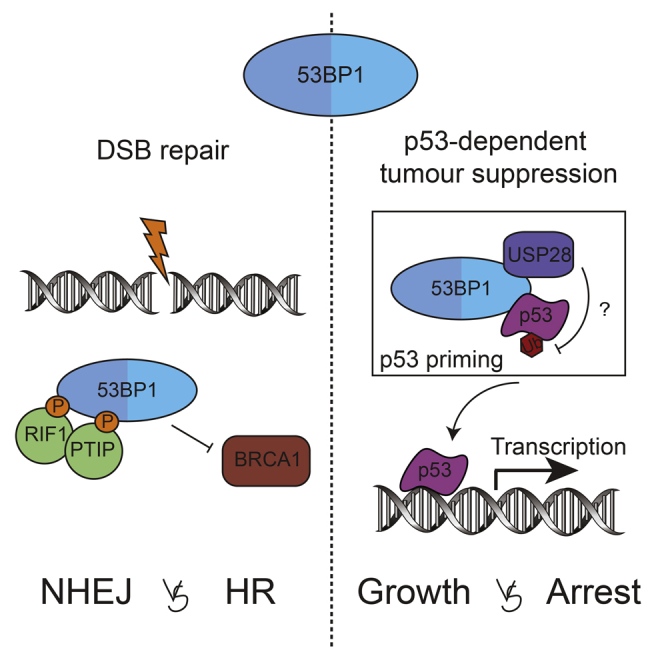

## Teaser

53BP1 was first identified as p53 binding protein 1, yet the function of the p53-53BP1 interaction was unclear. Cuella-Martin et al. show that 53BP1 bridges interactions between p53 and the deubiquitinating enzyme USP28, promoting p53-DNA interactions to globally enhance p53-dependent transcriptional programs.

## Introduction

Tumor suppression requires the integration of molecular signals that collectively function to safeguard genome integrity, while preventing the inheritance of potentially oncogenic mutations within proliferating cell populations. The transcription factor p53 is an impressive integrator and propagator of anti-tumorigenic cellular signals. Responding to a variety of stress signals that include DNA damage and aneuploidy, p53 regulates the transcription of a multitude of target genes to elicit cell-cycle arrest, apoptosis, senescence, DNA repair, and metabolic responses (Toledo and Wahl, 2006, Vousden and Prives, 2009). p53’s importance as a tumor suppressor is well exemplified by estimates that *p53* mutations are founder and/or driver events in around 50% of all cancers. Given that p53 elicits life and death decisions in response to an array of stimuli, it is subject to exquisite control via multiple mechanisms involving an array of interaction partners. One such partner, p53-binding protein 1 (53BP1) was first identified alongside ASPP2 (53BP2) as yeast two-hybrid interactors of the p53 DNA binding domain (DBD) (Iwabuchi et al., 1994). While ASPP2 has since been attributed vital tumor-suppressive roles in regulating p53’s pro-apoptotic responses (Samuels-Lev et al., 2001, Vousden and Prives, 2009), the regulatory role of p53-53BP1 interactions has remained ill defined.

In contrast, 53BP1 has been extensively characterized in the context of its contribution to the non-homologous end-joining (NHEJ) DNA double-strand break (DSB) repair pathway (Chapman et al., 2012, Zimmermann and de Lange, 2014). 53BP1 is an oligomeric chromatin reader that binds a combination of methylated (H4 Lys20me1/2) and ubiquitinated histone (H2A ubi-Lys15) epitopes within DSB-associated chromatin to promote NHEJ (Fradet-Turcotte et al., 2013). Its key function during DSB repair is to inhibit the nucleolytic processing of DNA ends, which it accomplishes by interactions with its downstream effector proteins, including Rif1 and PTIP (Zimmermann and de Lange, 2014). 53BP1-dependent NHEJ is crucial in the immune system, where it is necessary for immunoglobulin class switch recombination (CSR) and T cell receptor rearrangements (Difilippantonio et al., 2008, Manis et al., 2004, Ward et al., 2004), but this same process is oncogenic in cancers driven by *BRCA1* mutation or loss (Bouwman et al., 2010, Bunting et al., 2010). In these circumstances, 53BP1 blocks the accurate homologous recombination repair of DSBs and supports unrestrained NHEJ activity resulting in the massive genome rearrangements that drive oncogenesis (Chapman et al., 2012).

53BP1 also possesses tumor suppressor functions, and germline *53BP1* mutations predispose mice to T cell lymphoma, in a manner exacerbated by p53 loss (Morales et al., 2006, Ward et al., 2005). Thymic lymphomas derived from *53BP1*^*−/−*^ *p53*^*−/−*^ mice fall into two distinct cytogenetic categories: those characterized by aneuploidy that largely resemble equivalent tumors from *p53*^*−/−*^ mice (Liao et al., 1998) and those without aneuploidy that harbor clonal translocations thought to arise from DSB repair intermediates that accumulate as a result of abortive antigen receptor gene-rearrangements and progress into oncogenic translocations when p53-dependent apoptotic responses are ablated (Difilippantonio et al., 2008, Morales et al., 2006, Ward et al., 2005). While these results indicate a synergy between 53BP1 and p53 function, several lines of evidence have suggested putative roles for 53BP1 and p53 cooperation in tumor suppression (Huang et al., 2007, Iwabuchi et al., 1994, Iwabuchi et al., 1998). Interestingly, 53BP1 was identified in a genetic screen designed to reveal components of the p53 network that mediate cytotoxicity in response to Nutlin-3 (N3) (Brummelkamp et al., 2006). N3 is a small molecule antagonist of p53’s inhibitor protein MDM2 that, by binding to MDM2, outcompetes its interaction with p53 resulting in p53 stabilization and activation (Vassilev et al., 2004). 53BP1 depletion conferred resistance to N3-induced senescence in MCF-7, a human breast cancer cell line typically used to model wild-type (WT) p53 function, providing strong evidence for a physiological 53BP1-p53 cooperation. Despite this, the functional relevance and molecular basis of such cooperation, and its relationship to 53BP1’s DNA repair roles, has remained enigmatic.

Here, we use the synthetic viability phenotype of 53BP1-deficient cells in the presence of N3 as a means to dissect the molecular basis of 53BP1’s p53-regulatory roles in the absence of crosstalk from its repair functions. We reveal a role for 53BP1 in directly modulating p53’s transcriptional activities in response to multiple stimuli and find this is genetically, biochemically, and functionally separable from its DSB repair functions. Thus, 53BP1 integrates both p53-dependent functions and DNA repair activities to promote tumor suppression.

## Results

### 53BP1 Is Required for Optimal p53-Dependent Transactivation Events

**Figure 1:**
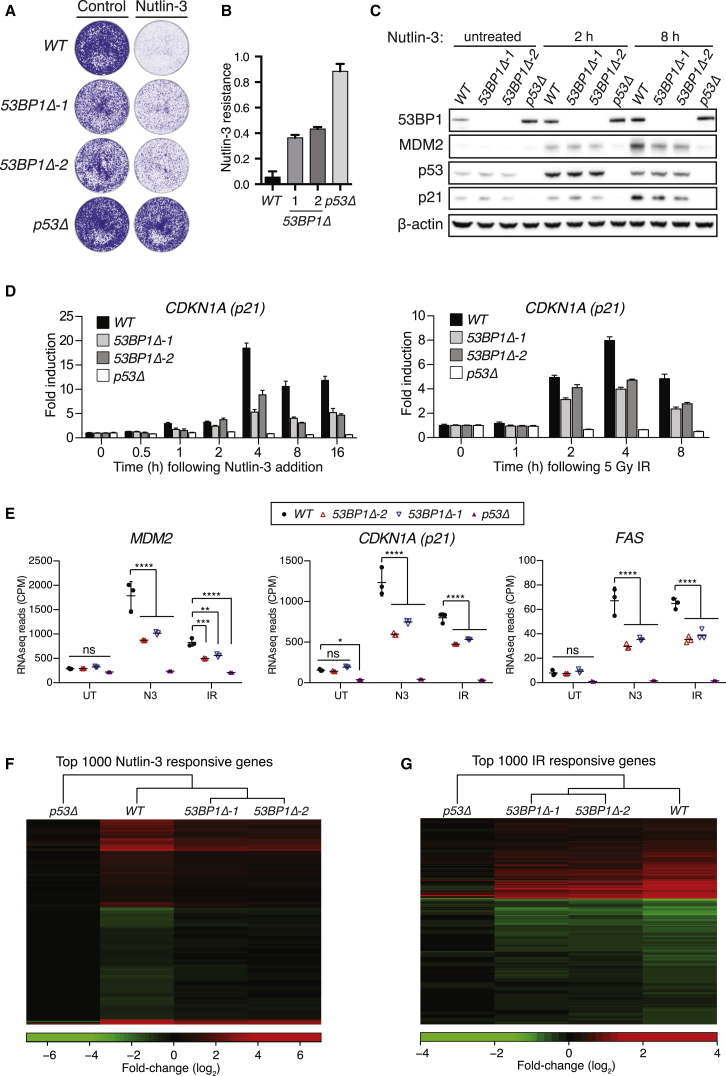
53BP1 Enhances p53-Dependent Transcriptional Programs (A) Cells of indicated genotype were treated with 4 μM N3 for 7 days or mock treated, fixed, and stained with crystal violet. (B) Quantification of two experiments as in (A), each plated in triplicate (mean ± SD). (C) Immunoblot analysis of MCF-7 lines of indicated genotype following exposure to N3. (D) *p21* transcript abundance in RNA isolates from cells treated with 4 μM N3 or 5 Gy IR, as evaluated by qRT-PCR. Fold induction calculated upon normalization against *HPRT1* transcripts. Data are representative of two independent experiments; mean ± SD. (E) Representative p53-responsive transcripts from three RNA-seq replicates. Total RNA was sequenced from indicated MCF-7 lines following N3 (4 μM, 8 hr), IR (5 Gy, 4 hr), or control treatments. CPM, counts per million; *ns*, non-significant; ^∗^p < 0.05; ^∗∗^p < 0.01; ^∗∗∗^p < 0.001; ^∗∗∗∗^p < 0.0001 (two-way ANOVA). Bars represent mean ± SD. (F and G) Heatmaps depicting log_2_ fold changes for top 1,000 responsive genes for each treatment relative to untreated controls (RNA-seq analysis of three biological replicates per condition). Ribo-depleted RNA was sequenced from indicated MCF-7 lines following N3 (4 μM 8 hr), IR (5 Gy, 4 hr), or control treatments. See also Figures S1 and S2 and Table S1.

To investigate 53BP1’s contribution to p53-dependent signaling events, multiple isogenic *TP53BP1*-knockout lines were generated using CRISPR-Cas9 technology in MCF-7 (henceforth termed *53BP1Δ*; Figure S1A). In parallel, *TP53*-knockout lines were generated (*p53Δ*; Figure S1B) to control for p53 loss of function. As expected (Brummelkamp et al., 2006), *53BP1Δ* cells show reduced N3-induced growth arrest (Figure 1A). This reduced sensitivity to N3 was partial when compared to *p53Δ* lines (∼40%; Figure 1B). Consistent with a disruption of p53 function in *53BP1Δ* cells, the N3-induced expression of the p53 targets p21 and MDM2 was strongly perturbed in 53BP1-deficient lines (Figure 1C). The fact that equivalent defects in MDM2 and p21 induction were also apparent following IR treatment (Figure S1C) suggested that 53BP1-mediated p53 regulation was not N3 specific, and 53BP1 likely modulates broader p53 function.

Next, 53BP1’s contribution to p53-dependent transcriptional outputs was examined by quantitative RT-PCR. The induced expression of *p21* (*CDKN1A*) transcripts was consistently reduced by ∼2-fold in *53BP1Δ* cells across all time points examined following each treatment (Figure 1D). Likewise, 53BP1 deficiency resulted in induction defects in other p53-responsive genes, including auto-regulatory (*MDM2*), cell-cycle (*GADD45A*, *TP53I3*), and pro-apoptotic (*BAX*, *PUMA/BBC3*) targets (Figure S1D). To characterize 53BP1’s contribution to p53-dependent transcriptional programs genome wide, total RNA from WT, *p53Δ*, and two different *53BP1Δ* cell lines was analyzed by RNA-sequencing (RNA-seq) following mock, N3, and IR treatments. N3 and IR treatments induced significant alterations to the expression of 6,877 and 2,386 genes, respectively, in WT MCF-7, encompassing both gene activation and repression events (summarized in Table S1). These changes were almost exclusively p53 dependent, as in *p53Δ* cells N3 or IR induced significant changes to the abundance of only 13 and 25 transcripts, respectively. RNA-seq confirmed that N3- and IR-induced transactivation of p53-responsive genes was strongly attenuated in *53BP1Δ* cell lines (Figure 1E). *53BP1Δ* cell lines also displayed defects in p53-dependent gene repression events (Figure S2A). Moreover, the p53-dependent transcriptional programs induced by both treatments were dramatically impaired in 53BP1-deficient cells, with defects in gene activation and repression evident across p53-responsive genes (Figures 1F, 1G, S2B, and S2C). These defects are not due to differences in global transcription, as the abundance of non-treatment-responsive transcripts (Figure S2D) and candidate reference transcripts (Figure S2E) was equivalent across the different genotypes, irrespective of treatment. Taken together, these results indicate a key role for 53BP1 in propagating p53-dependent transcriptional programs. That this effect is seen genome wide excludes a role for 53BP1 in modulating p53 target specificity but rather implies that 53BP1 is a global enhancer of p53-dependent signals.

### Structural Requirements for 53BP1-Dependent p53 Regulation

**Figure 2:**
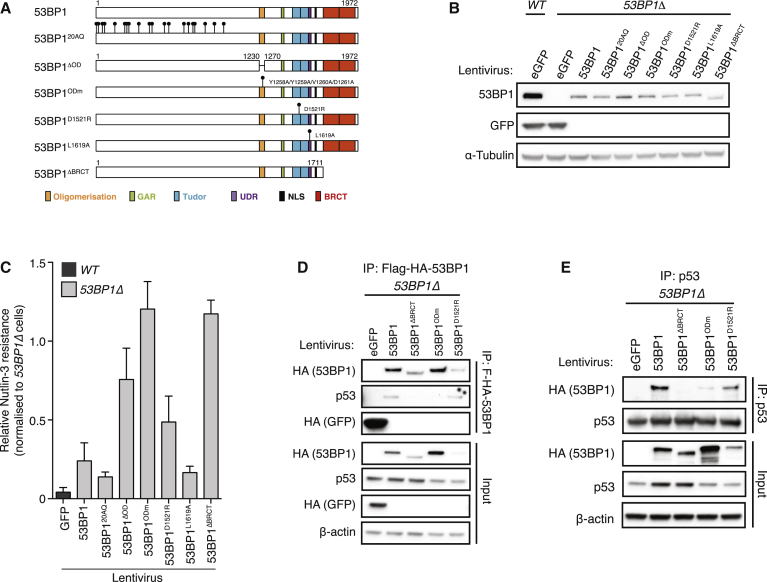
53BP1-Dependent p53 Regulation Requires Oligomerization and BRCT Domain-Mediated p53 Interactions (A) Schematic representation of the 53BP1 domain and point mutants examined for restoration of N3 sensitivity. (B) Western blot showing comparable expression of 53BP1 mutants upon stable lentivirus-mediated transduction in *53BP1Δ* MCF-7. (C) Indicated cell lines treated with N3 (4 μM) for 11 days or left untreated for 7 days were fixed and stained with crystal violet. Relative N3 resistance normalized to control (GFP)-complemented *53BP1Δ*. Mean of three biological replicates ± SD. (D) Flag-HA-53BP1 proteins purified from cell lysates of indicated stably complemented *53BP1Δ* MCF-7 lines following N3 treatment. Interacting proteins analyzed by immunoblotting. (E) Similar to (D), but the composition of p53 immunoprecipitates was analyzed. See also Figure S3.

53BP1 is a protein scaffold and chromatin reader whose modular domain architecture supports binding to an array of interaction partners including post-translationally modified histones (Fradet-Turcotte et al., 2013, Zimmermann and de Lange, 2014). The individual contributions of 53BP1’s domains, motifs, and interaction partners toward its DNA repair roles have been well characterized yet remain uncharacterized in the context of p53 modulation. To address this, we generated *53BP1Δ* lines in which equivalent levels of WT and mutant 53BP1 proteins, or control protein (GFP), are stably expressed (Figures 2A and 2B) and assessed their ability to rescue N3 sensitivity. WT 53BP1 expression largely restored N3 sensitivity, when compared to control GFP-expressing *53BP1Δ* cells (Figure 2C). Likewise, expression of the 53BP1^L1619A^ ubiquitin-dependent recruitment (UDR) motif mutant, whose recruitment to DSB sites is compromised (Fradet-Turcotte et al., 2013), or the 53BP1^20AQ^ mutant that confers NHEJ defects owing to an inability to sustain Rif1 and PTIP recruitment at DSB sites (Callen et al., 2013, Chapman et al., 2013, Di Virgilio et al., 2013) both restored cellular N3 sensitivity to equivalent WT levels. In contrast, 53BP1 mutants bearing a deleted or mutated oligomerization domain (53BP1^ΔOD^ or 53BP1^ODm^) or a C-terminal tandem-BRCT domain deletion (53BP1^ΔBRCT^) failed to restore N3 sensitivity, highlighting the important contribution of these two domains in N3 responses. Interestingly, an intermediate N3 sensitivity was detected in *53BP1Δ* cells complemented with the 53BP1^D1521R^ tudor domain mutant, indicating methyl-lysine-directed interactions with p53 (Huang et al., 2007, Kachirskaia et al., 2008), retinoblastoma protein pRb (Carr et al., 2014), and/or histone H4 methylated on lysine 20 (H4K20me1/2) (Botuyan et al., 2006) might additionally influence p53-dependent transcription. Oligomerization, tudor, and BRCT domain mutant 53BP1 proteins also showed their expected localization patterns, indicating that their inability to restore WT N3 sensitivity is not due to aberrant protein expression or localization (Figure S3).

Given the importance of the 53BP1 oligomerization, BRCT, and tudor domains in conferring N3 sensitivity, the function of each domain in 53BP1-p53 protein interactions was examined in co-immunoprecipitation experiments. Flag-HA-tagged WT or tudor mutant (53BP1^D1521R^) 53BP1 proteins both co-precipitated p53 from cell lysates (Figure 2D). Hence, reported tudor domain-mediated binding to p53 methyl-lysine residues (Huang et al., 2007, Kachirskaia et al., 2008) does not mediate bulk cellular p53-53BP1 interactions. In contrast, p53 was undetectable in 53BP1^ΔBRCT^ immunoprecipitates or when multiple conserved residues within the 53BP1 oligomerization domain were mutated (Zgheib et al., 2009) (Figure 2D). Concordant p53-53BP1 interaction profiles were reproduced upon immunoprecipitation of endogenous p53 from each mutant 53BP1 cell lysate (Figure 2E). These data indicate that the 53BP1 tandem-BRCT domain is responsible for interacting with p53, and this interaction is significantly destabilized by mutation of the 53BP1 oligomerization domain. Combined with our N3 sensitivity data (Figure 2C), this shows that p53-53BP1 interactions are essential for normal p53 function.

### 53BP1-Dependent p53 Regulation and DSB Repair Activities Are Distinct and Separable

**Figure 3:**
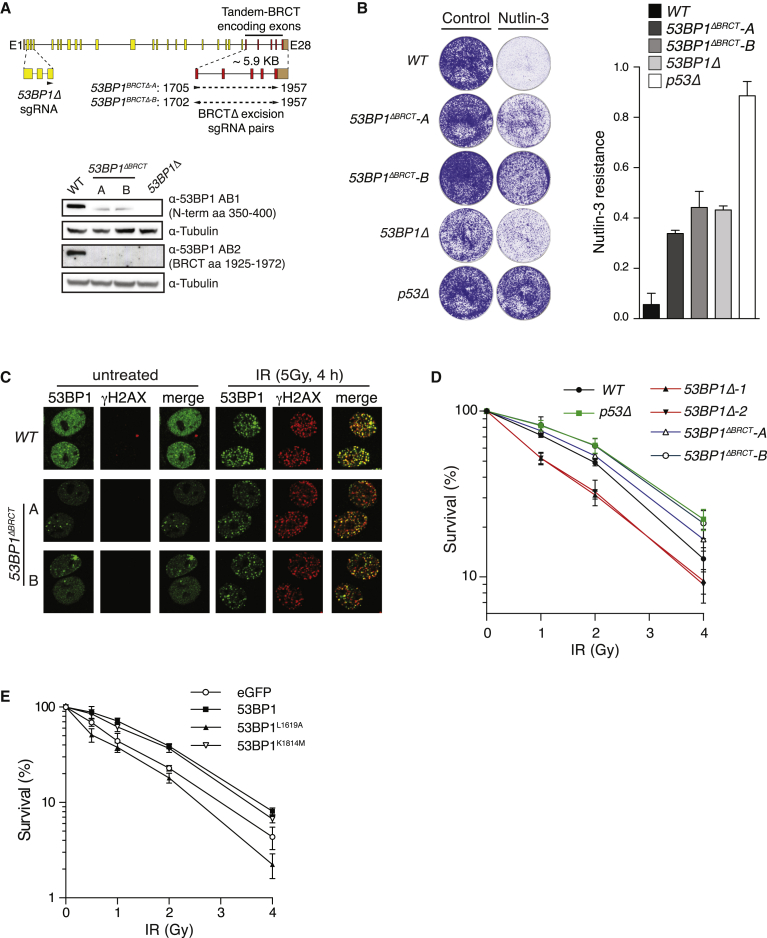
53BP1’s p53-Regulatory and DSB Repair Activities Are Distinct and Separable (A) Generation of *53BP1*^*ΔBRCT*^ alleles using CRISPR-Cas9 technology. Top: schematic representation of *TP53BP1* gene locus showing the two sgRNA pairs (triangles) used to excise BRCT-encoding exonic sequences. Bottom: immunoblot of lysates prepared from two *53BP1*^*ΔBRCT*^ MCF-7 lines with epitope-specific 53BP1 antibodies showing the expression of mutant 53BP1^ΔBRCT^ protein. (B) N3 resistance assay was performed as in Figures 1A and 1B. Mean ± SD (n = 2, plated in triplicate). (C) Subnuclear 53BP1 localization was analyzed by indirect immunofluorescence in indicated cell lines following mock or irradiation (5 Gy, 4 hr) treatments. (D) The survival of MCF-7 cell lines of indicated genotype following control or X-ray irradiation treatments was assessed by colony survival assay. Mean ± SD (n = 3, plated in triplicate). (E) The survival of *53BP1Δ* cells stably complemented with indicated 53BP1 transgenes following control or X-ray irradiation treatments was assessed as in (D). Mean ± SD (n = 3, plated in triplicate). See also Figure S4.

Mutant *53BP1* alleles that encode C-terminal BRCT domain deletion mutations are indistinguishable from WT in their ability to support physiological NHEJ during CSR and pathological NHEJ events in the context of *BRCA1* deficiency and dysfunctional telomeres (Bothmer et al., 2011, Lottersberger et al., 2013). However, several recent reports have suggested roles for the 53BP1 BRCT domain in heterochromatin DSB repair (Baldock et al., 2015, Lee et al., 2010, Noon et al., 2010). Given the importance of this domain in mediating functional p53 interactions, we next explored the relative contributions of this domain toward p53 regulation and DSB repair. Paired guide RNA molecules were used to direct the Cas9-dependent excision of genomic sequences encoding 53BP1’s BRCT domain (Figure 3A), and two validated isogenic clones were further characterized (denoted *53BP1*^*ΔBRCT*^, clones A and B). Consistent with previous work in which the 53BP1-BRCTs were deleted in the mouse germline (Bothmer et al., 2011), 53BP1 expression in *53BP1*^*ΔBRCT*^ cell lines was reduced relative to WT (Figure 3A). Nevertheless, *53BP1*^*ΔBRCT*^ cells proliferated at a normal rate in comparison to slow-growing *53BP1Δ* cells (Figure 3B and data not shown), suggesting that reduced 53BP1^ΔBRCT^ protein levels can support normal cell growth. Despite this, *53BP1*^*ΔBRCT*^ lines were N3 resistant like *53BP1Δ* cells (Figure 3B) and showed attenuated p53-dependent induction of MDM2 and p21 following N3 and IR treatments (Figures S4A and S4B), confirming the importance of an intact BRCT domain in modulating p53 function. As the BRCT domains are largely dispensable for 53BP1-dependent NHEJ (Bothmer et al., 2011, Lottersberger et al., 2013), we reasoned the normal growth rate of *53BP1*^*ΔBRCT*^ lines indicated that 53BP1^ΔBRCT^ expression could support 53BP1-dependent repair activities. In line with this, 53BP1^ΔBRCT^ protein was recruited into IRIF (Figure 3C), where it supported normal Rif1 recruitment (Figure S4C), events essential for its canonical NHEJ functions (Chapman et al., 2013, Di Virgilio et al., 2013, Escribano-Díaz et al., 2013, Zimmermann et al., 2013). To investigate this potential separation of function further, the relative IR sensitivity of WT, *53BP1Δ*, *p53Δ*, and *53BP1*^*ΔBRCT*^ cell lines was compared (Figure 3D). As expected (Chapman et al., 2013, Iwabuchi et al., 2006), asynchronous *53BP1Δ* cultures were moderately IR sensitive (Figure 3D). Remarkably, however, *53BP1*^*ΔBRCT*^ lines showed significantly improved survival following IR treatments when compared to WT cells, closely mimicking the radioresistance evident in *p53Δ* cells (Figure 3D). It is known that p53 deficiency bypasses apoptotic and cellular senescence responses following IR treatments (Krenning et al., 2014, Lowe et al., 1993). Our data thus suggest that 53BP1 BRCT-mediated p53 interactions positively influence these functions. We therefore speculated that the expression of 53BP1 mutants that only disrupt its repair activities might further increase the sensitivity of *53BP1Δ* cells to IR treatments by restoring their ability to promote p53-dependent cell-cycle arrest or exit responses. In line with this, stable expression of the 53BP1 UDR mutant 53BP1^L1619A^, which is unable to assemble at DSB sites (Fradet-Turcotte et al., 2013) yet is proficient for N3 responses, increased the radiosensitivity of *53BP1Δ* cells, in contrast to WT 53BP1 expression that suppressed IR sensitivity (Figure 3E). These data therefore reveal a major function for the 53BP1 BRCT domain in regulating p53-dependent cell-cycle exit responses and argue that these activities function independently of 53BP1-dependent DNA repair.

### The 53BP1 BRCT Domain Mediates Bivalent Interactions with p53 and USP28

**Figure 4:**
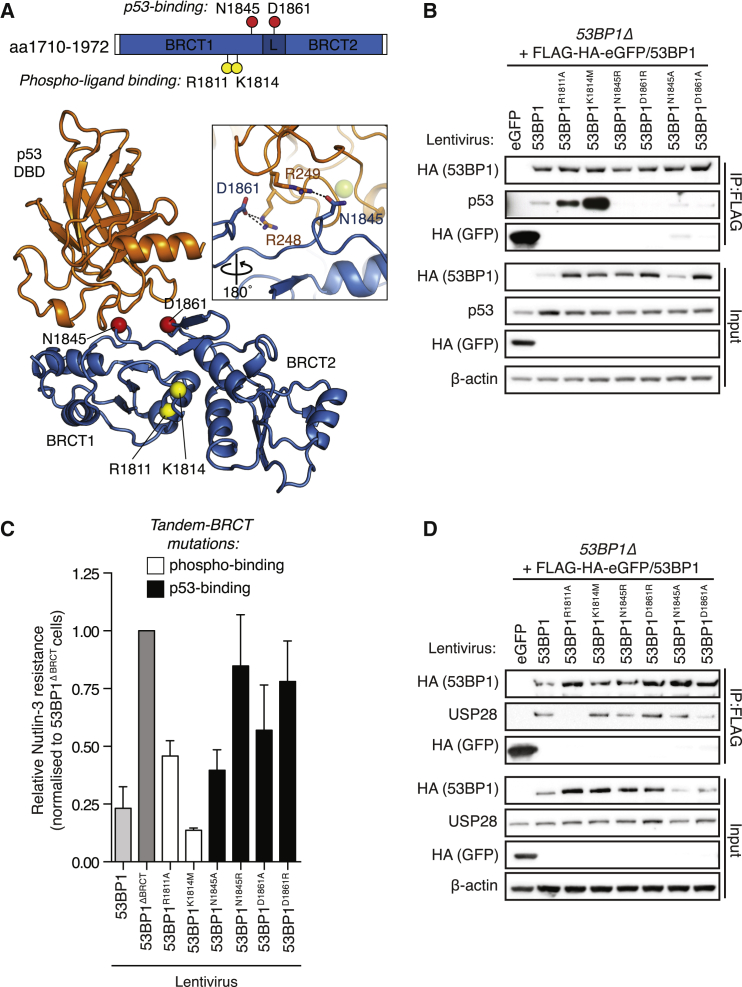
The 53BP1 BRCT Domain Mediates Bivalent Interactions with p53 and USP28 (A) The p53-53BP1 co-crystal structure (PDB: 1KZY; Joo et al., 2002) indicates the distinct tandem-BRCT surface residues involved in p53- and phospho-ligand interactions (red and yellow spheres, respectively). Dotted lines in zoom panel indicate hydrogen bonds between residues in 53BP1 and p53. (B) The interaction of p53 with indicated FLAG-HA-53BP1 protein complexes was probed by immunoblotting, following immunopurification from N3-treated cell lysates. (C) N3 resistance of *53BP1Δ* cells complemented with indicated 53BP1 point mutants. N3 resistance was normalized to 53BP1^ΔBRCT^-complemented lines. Mean ± SD (n = 3, in triplicate). (D) As in (B), but FLAG-HA-53BP1 protein complexes were examined for USP28 co-purification. See also Figure S5.

Tandem-BRCT domains are common structural features of DDR proteins that typically possess a phosphopeptide-binding surface within the inter-BRCT repeat interface that interacts with phospho-serine-containing motifs in partner proteins (Reinhardt and Yaffe, 2013). Somewhat atypically, the 53BP1 tandem-BRCT-mediated p53 interaction utilizes the opposite face of the BRCTs, which contains conserved surface residues spanning the first BRCT (BRCT1) and the inter-BRCT linker that mediate multiple contacts with residues in the L3-loop of the p53 DBD (Figure 4A) (Joo et al., 2002). In the p53-53BP1 co-crystal structure, the conserved phospho-binding pocket within the 53BP1 tandem-BRCT domain remains available, suggesting additional BRCT-mediated protein interactions could contribute to p53 modulation. We thus investigated the contributions of the p53- and phospho-binding surfaces of the 53BP1 BRCTs in p53 responses. In validation of structural models (Derbyshire et al., 2002, Joo et al., 2002), alanine substitution of p53-binding residues Asn1845 and Asp1861 in 53BP1 (Figure 4A) weakened 53BP1-p53 interactions (Figure 4B). Moreover, p53-binding was more dramatically destabilized by bulky arginine substitutions at these positions (Figure 4B). Notably, the differential effects of Ala and Arg mutations at these positions on p53 binding were consistent with the greater N3 resistance of 53BP1^N1845R^- and 53BP1^D1861R^-expressing *53BP1Δ* lines, compared to their 53BP1^N1845A^- and 53BP1^D1861A^-expressing counterparts (Figures 4C and S5), confirming the importance of these residues in regulating p53 function. In contrast, p53-53BP1 interactions were enhanced by 53BP1^R1811A^ and 53BP1^K1814M^ phospho-binding pocket mutations (Figures 4A and 4B), suggesting the 53BP1 BRCTs might bind p53 and phospho-ligands within distinct and competing protein complexes. Intriguingly, 53BP1^R1811A^ expression could only partially suppress N3 resistance in *53BP1Δ* cells (Figure 4C), which was in contrast to 53BP1^K1814M^ expression that rescued N3 sensitivity to levels equivalent to WT-53BP1 complementation. This suggested that Arg1811 might support interactions with an additional 53BP1 interactor independently of Lys1814, despite their usual cooperation in phospho-peptide binding (Baldock et al., 2015, Kleiner et al., 2015). The Ubiquitin Specific Protease USP28 is a reported interactor of the 53BP1 BRCT domain (Knobel et al., 2014, Zhang et al., 2006), and a USP28-targeting shRNA construct was modestly enriched in the same loss-of-function screen that identified 53BP1 as a suppressor of N3-induced senescence (Brummelkamp et al., 2006). We found that 53BP1 binding to USP28 was specifically attenuated by the R1811A BRCT mutation, yet unaffected by the K1814M phospho-binding or p53-binding mutations (Figure 4D). These data show that 53BP1 binds independently to p53 and USP28 via distinct BRCT domain surfaces and pointed toward a potential cooperative role for USP28-53BP1 complexes in p53 regulation.

### USP28 Is a Component of the p53-53BP1 Axis

**Figure 5:**
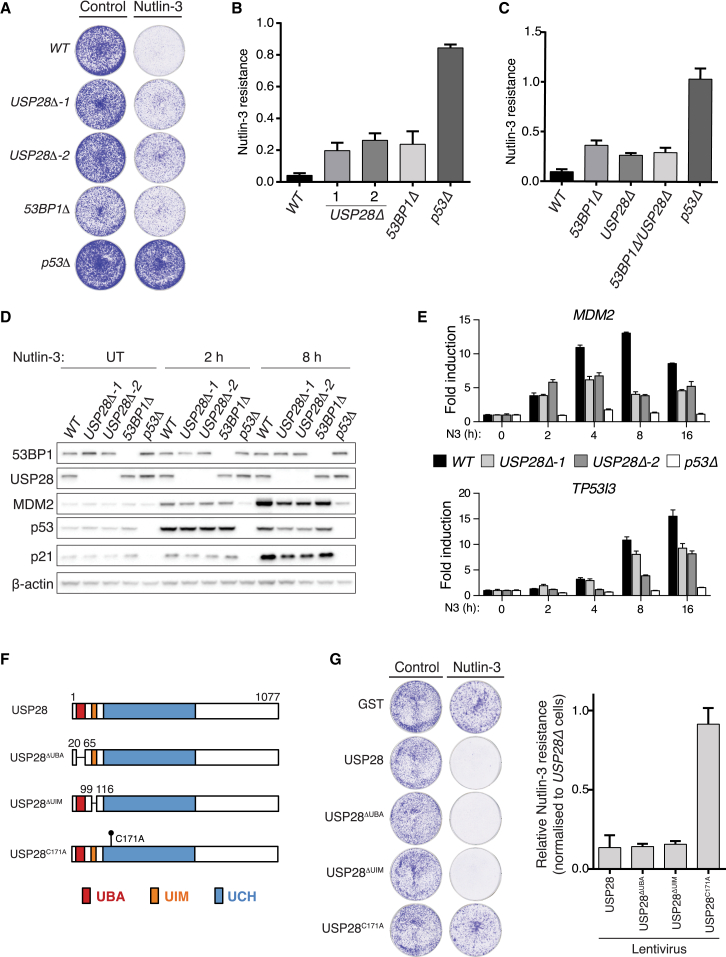
USP28 Is a Component of the p53-53BP1 Axis (A) N3 resistance of indicated cell lines assessed as in Figures 1A and 1B. (B) Quantification of (A). Mean ± SD (n = 3). (C) N3 resistance of a *53BP1Δ*, *USP28Δ* double-knockout cell line relative to single mutants. Mean ± SD (n = 3, in triplicate). (D) Lysates prepared from indicated control and N3-treated cell lines were immunoblotted with indicated antibodies. (E) The transactivation of p53-responsive genes *MDM2* and *TP53I3* was assessed by qRT-PCR in indicated cell lines. Indicated fold changes were calculated upon normalization against *HPRT1* transcripts. Data are representative of two independent experiments; mean ± SD. (F) Schematic representation of the USP28 protein domain architecture and the domain and point mutants used in this study. UBA, ubiquitin-associated domain; UIM, ubiquitin-interaction motif; UCH, ubiquitin carboxyl-terminal hydrolase domain. (G) Visual and quantitative analysis of N3 resistance of *USP28Δ* cell lines stably transduced with indicated USP28 expressing lentiviruses. N3 resistance relative to a control (GST)-transduced *USP28Δ* cell line was calculated as in (A) and (B). Mean ± SD (n = 3, in triplicate). See also Figure S6.

USP28 was originally implicated in counteracting the proteasomal degradation of 53BP1, CHK2, and multiple additional DDR proteins (Zhang et al., 2006). However 53BP1’s stability or repair activities have since been found to be unaffected by germline *USP28* deletion in mice (Knobel et al., 2014). We thus considered if USP28 might cooperate with 53BP1 in regulating p53. Multiple isogenic *USP28Δ* MCF-7 lines generated using CRISPR-Cas9 (Figure S6A) displayed N3 resistance at levels equivalent to *53BP1Δ* cells (Figures 5A and 5B). Moreover, the N3 resistance of a *USP28Δ 53BP1Δ* double-knockout line was not enhanced over single mutants (Figure 5C), confirming USP28 and 53BP1 function epistatically in this context. Further consistent with USP28’s participation in p53-dependent signaling, *USP28Δ* cells were defective at inducing MDM2 and p21 protein expression following N3 treatment (Figure 5D), correlating with an impaired stimulation of p53-dependent transcription under similar conditions (Figure 5E). Lastly, USP28 deubiquitinase (DUB) activity was essential in this context, as restored expression of WT USP28, or mutants in which its N-terminal ubiquitin-binding UBA or UIM domains were deleted, restored N3 sensitivity, while the catalytic dead USP28^C171A^ mutant (Zhang et al., 2006) could not (Figures 5F and 5G). Consistent with this, the expression of WT or ubiquitin-binding mutant USP28 rescued p21 and MDM2 induction defects in *USP28Δ* cells, whereas expression of USP28^C171A^ did not (Figure S6B). Together, these data reveal a co-regulatory role for 53BP1 and USP28 in supporting p53 function.

### 53BP1 and USP28 Are Co-regulators of p53-Dependent Cell-Cycle Checkpoints

**Figure 6:**
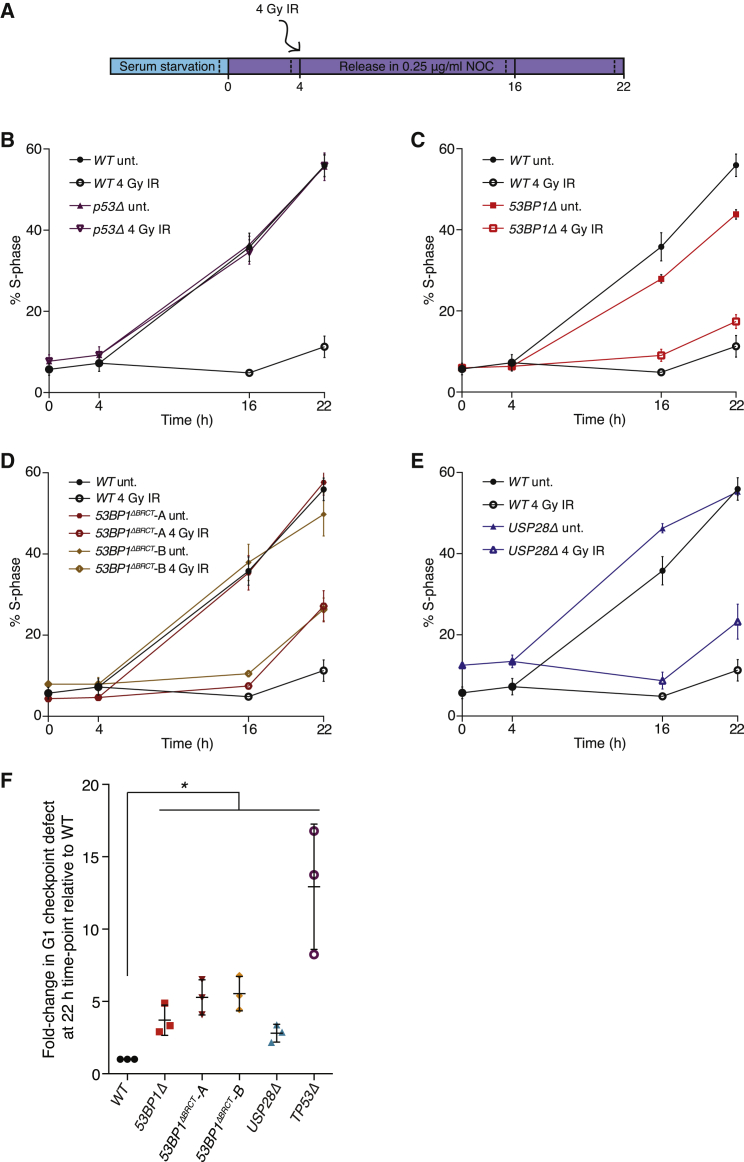
53BP1 and USP28 Co-regulate p53-Dependent G1 Checkpoint Arrest (A) Schematic representation of the G1 checkpoint assay. Briefly, cells serum arrested in G0 for 24 hr were released in serum-containing medium supplemented with 0.25 μg/ml nocodazole before irradiation (4 Gy) and collection at indicated time points for cell-cycle analysis. Solid bars indicate experimental time points. Dotted lines indicate time of BrdU pulse addition. (B–E) S phase cell indices for each indicated condition as defined by BrdU pulse labeling immediately prior to collection at indicated time points. Relative cell-cycle phase distributions were calculated by flow cytometry in BrdU immunolabeled cells counterstained for DNA content. Three biological replicates mean ± SD. (F) Significance of G1 checkpoint defects detected in (B)–(E) as a measurement of changes in S phase indices in irradiated samples between 4 and 22 hr time points. IR-treated sample values first corrected against the S phase index change in the corresponding untreated sample were then normalized against the WT value for each experiment. Values are plotted as fold change relative to WT. Mean ± SD; ^∗^p < 0.05 (Student’s t test).

Central to p53’s suppression of tumor growth is its ability to sustain cell-cycle arrest and exit responses following stress signals (Vousden and Prives, 2009). The cooperation between 53BP1 and USP28 in triggering p53-dependent growth arrest following N3 treatments prompted us to investigate their function in arresting the cell cycle in response to DNA damage. p53-dependent p21 upregulation is essential for G1-phase arrest in response to irradiation (Dulić et al., 1994), and 53BP1 has additionally been implicated in enforcing this checkpoint in a manner involving interactions with the TopBP1 checkpoint protein (Cescutti et al., 2010). To examine the relative contributions of 53BP1, its BRCT domains, and USP28 to G1 checkpoint activation, the progression of serum-released WT, *53BP1Δ*, *53BP1*^*ΔBRCT*^, and *USP28Δ* cultures into S phase was monitored following mock or IR treatments (Figure 6A). While untreated WT cultures readily proceeded from G1 into S following serum release, irradiated cells remained arrested in G1 (Figure 6B), consistent with robust activation of the G1 checkpoint. As expected (Dulić et al., 1994), G1 arrest was completely p53 dependent; IR-treated *p53Δ* cells re-entered the cell cycle with kinetics indistinguishable from non-irradiated controls (Figure 6B). The G1 checkpoint activation was strongly influenced by 53BP1 and its BRCT domains, as a significant proportion of *53BP1Δ* and *53BP1*^*ΔBRCT*^ cell lines entered S phase at time points where WT cells remained robustly arrested, with escape from G1 arrest increasing to around half of all cells at 22 hr following irradiation (Figures 6C and 6D). These defects correlate well with the intermediate effects that were seen for 53BP1-deficient and 53BP1^ΔBRCT^ mutant cell lines in N3 and IR responses, indicating that while 53BP1 and its BRCT-mediated p53 interactions function to reinforce p53 function, some residual p53 function remains in the absence of this regulation. Similar intermediate G1 checkpoint defects were observed in *USP28Δ* cells (Figures 6E and 6F), revealing a role for USP28 in regulating p53-dependent checkpoint responses. Thus, our data identify an important role for 53BP1-bridged p53-USP28 interaction in regulating p53 function in human cells.

### 53BP1 and USP28 Co-stimulate p53-Responsive Element Interactions

**Figure 7:**
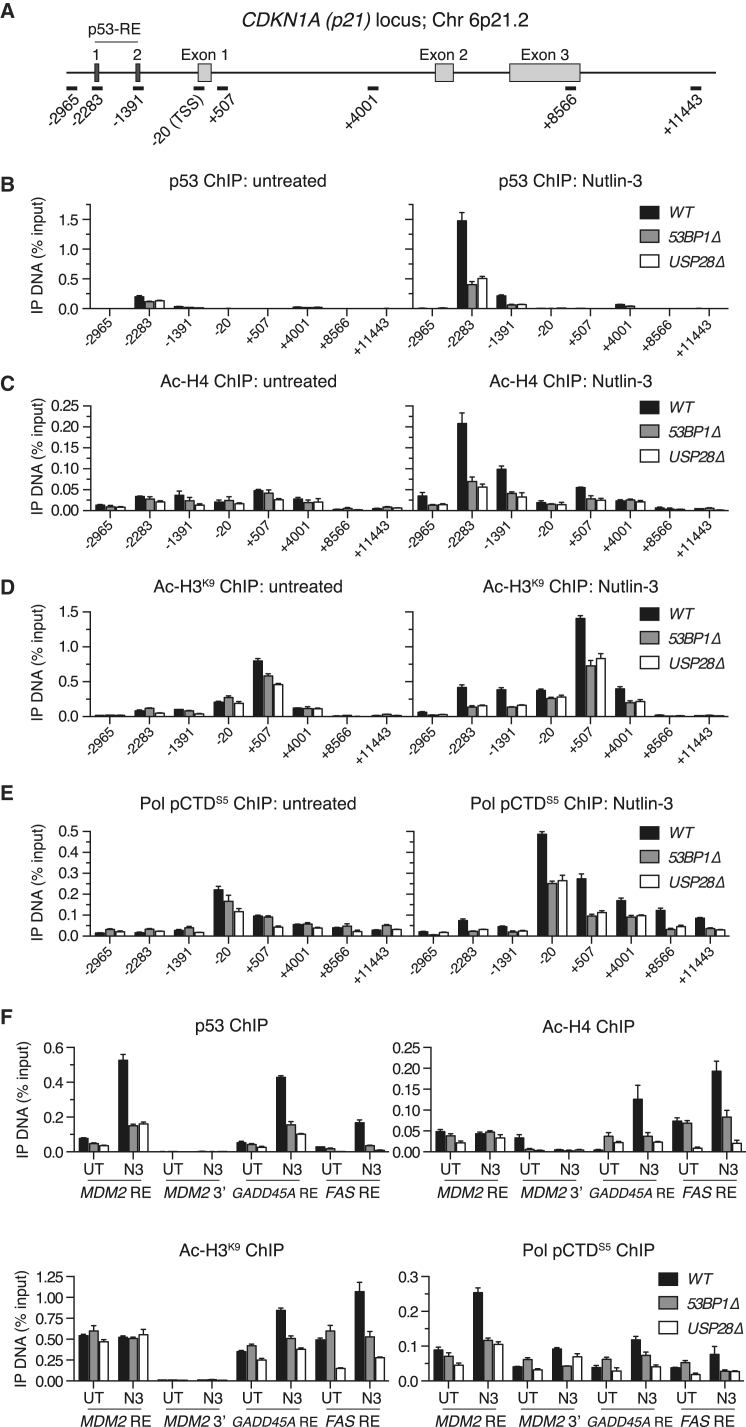
53BP1 and USP28 Enhance p53-Responsive Element Interactions (A) Schematic of the *p21* (*CDKN1A*) locus indicating high and low affinity p53 binding sites (p53-REs 1 and 2, respectively) and general gene structure. qPCR amplicons used to quantify ChIP-enriched DNA are indicated (bars) and named according to their relative distance to the transcription start site (TSS). (B–E) ChIP was performed in chromatin extracts prepared from indicated untreated or N3-treated (4 μM, 6 hr) cell lines using antibodies against p53 (B), pan-acetyl-H4 (C), acetyl-histone H3 Lys9 (D), and RNAP2 CTD phosphorylation (pSer5) (E). Immunoprecipitated DNA was calculated as a percentage of total input DNA. Data are representative of two independent experiments with PCRs performed in triplicate. Mean ± SD. (F) As in (B)–(E), but using p53-RE-spanning amplicons in indicated p53-responsive genes, except MDM2 3′, which indicates a control amplicon within the last *MDM2* coding exon. See also Figure S7.

We next considered the mechanism by which 53BP1-USP28 complexes might modulate p53-dependent transcription. p53 transcriptional activities under basal and stimulated conditions are fine-tuned via regulated changes to p53’s stability, subcellular localization, and DNA-binding activities (Kruse and Gu, 2009, Vousden and Prives, 2009). However, we were unable to link the defects we detected to alterations in the stability (Figures S7A and S7B) or sub-cellular localization (Figure S7C) of p53 in untreated or irradiated *53BP1Δ* or *USP28Δ* cells. To determine the contribution of 53BP1-USP28 to p53-responsive element (p53-RE) binding, we performed chromatin immunoprecipitation (ChIP) experiments under basal and N3-stimulated conditions. N3 stimulated ∼7-fold increases in p53 binding to its two binding sites in the *p21* promoter in WT cells (Figures 7A and 7B). Both of these binding events strongly relied on 53BP1 and USP28 status. Specifically, basal p53 binding to both p53 REs was reduced by ∼2-fold in both *53BP1Δ* and *USP28Δ* cells, and its induction upon N3 treatment was severely impaired, resulting in overall ∼3-fold reductions in p53 residency at both loci upon stimulation (Figure 7B). These defects corresponded to a diminished induction of histone acetylation events associated with p53-dependent gene transactivation (Donner et al., 2007). Specifically, histone H4 pan-acetylation (H4ac) across the *p21* promoter was reduced by ∼3-fold in N3-treated *53BP1Δ* and *USP28Δ* cultures relative to WT (Figure 7C), with equivalent defects detected in H3 K9 acetylation (H3 K9ac) across promoter and 5′ intragenic regions (Figure 7D). These abnormalities correlated to diminished elongating RNA Pol2 residency across the *p21* gene body in *53BP1Δ* and *USP28Δ* cells (Figure 7E), a defect entirely consistent in magnitude with the p53-dependent transcriptional defects detected in earlier experiments (Figures 1F, 1G, and 5E). These abnormalities were not unique to the *p21* locus, as p53 DNA binding, histone acetylation, and RNA Pol2 residency defects were reproduced at p53 REs within multiple additional p53-responsive genes in *53BP1Δ* and *USP28Δ* cells (Figure 7F). In line with our transcriptomic analyses (Figure 1), our data collectively reveal a function for 53BP1-dependent bivalent interactions with USP28 and p53 in enhancing p53-promoter element interactions, thereby amplifying p53-dependent transcriptional programs.

## Discussion

The repair activities of 53BP1 synergize with p53 in tumor suppression (Difilippantonio et al., 2008, Morales et al., 2006, Ward et al., 2005), yet interactions between 53BP1 and p53 have hinted at additional cooperative contributions. Taking advantage of the discovery that 53BP1 and p53 co-participate in N3-induced senescence responses (Brummelkamp et al., 2006), we now reveal the molecular basis of 53BP1-p53 cooperation. In contrast to initial speculation that p53-53BP1 cooperation relied on a synergy between p53-dependent transcriptional responses and the signaling of stochastic DNA damage via 53BP1 (Brummelkamp et al., 2006), we find that 53BP1 plays a direct role in enhancing the magnitude of p53-dependent gene activation and repression events triggered by N3 and IR. Perhaps surprisingly, 53BP1-dependent p53-regulatory and DNA-repair activities can be entirely separated at the level of mutations in 53BP1 that selectively either block p53 binding or prevent its enrichment at DSB sites and interaction with downstream repair effector proteins. Thus, our data demonstrate that 53BP1 DNA repair and p53-regulatory roles are independent of one another and rely on thier association with distinct interaction partners. This separation of function is best illustrated by mutation of 53BP1’s tandem-BRCT domain, where our data are consistent with previous models in which the 53BP1 tandem-BRCT domain is dispensable for its canonical repair activities (Bothmer et al., 2011, Lottersberger et al., 2013, Ward et al., 2006). In addition, by disrupting p53-53BP1 interactions with single point mutations, we have validated previous structural models of the p53-53BP1 interaction (Derbyshire et al., 2002, Joo et al., 2002) and revealed that these interactions are critical for optimal p53 function.

Recent evidence implicates the 53BP1 tandem-BRCT domain in binding the Rad50, ATM, and γH2AX proteins in interactions important for DSB resolution within heterochromatin (Baldock et al., 2015, Lee et al., 2010, Noon et al., 2010). In these studies, loss of this function resulted in persistent γH2AX foci in irradiated 53BP1 BRCT mutant-expressing cell lines. However, our data suggest that this defect plays no role in the IR hypersensitivity of *53BP1Δ* lines, as reconstitution of a 53BP1 BRCT domain phospho-ligand binding mutant (53BP1^K1814M^) that blocks γH2AX binding (Baldock et al., 2015, Kleiner et al., 2015) restored the IR resistance of *53BP1Δ* lines to WT levels (Figure 3E). In fact, the creation of *53BP1*^*ΔBRCT*^ alleles actually enhanced cellular survival following IR treatments relative to WT, correlating closely with the radioresistant phenotype we and others have described for cells of diminished p53 function (Figure 3D) (Lowe et al., 1993) while also reflecting an overall proficiency for 53BP1 BRCT mutant protein in supporting bulk 53BP1-dependent DNA repair activities. Thus, we propose that the direct regulation of p53 represents a prime function for the conserved 53BP1 tandem-BRCT domain.

In addition to distinct 53BP1 domain requirements for p53 and DNA repair regulation, some 53BP1 domain features were found to contribute to both roles. 53BP1’s recruitment to DSB sites is critical for its repair functions and involves auto-oligomerization and bivalent nucleosomal contacts (Fradet-Turcotte et al., 2013). Here, we report that 53BP1 oligomerization is similarly crucial for 53BP1-dependent p53 regulation. Mutations that prevent oligomerization also destabilize p53-53BP1 interactions (Figures 2D and 2E), suggesting 53BP1 oligomerization either reinforces BRCT-mediated p53 interactions or increases its avidity toward multimeric p53 complexes. Interestingly, 53BP1 UDR and tudor domain mutations elicit differential effects on p53 function, despite their cooperation during DSB repair. Specifically, 53BP1 UDR point mutants were able to fully rescue N3 sensitivity, while methyl-lysine-binding tudor domain mutants did so only partially. While the tudor domain’s participation in this context might be explained by its ability to mediate binding to methyl-K370/K382 residues in p53 (Huang et al., 2007, Kachirskaia et al., 2008), we were unable to detect any significant impact of tudor domain mutation on bulk 53BP1-p53 interactions, in contrast to previous findings (Huang et al., 2007). Despite this, it is noteworthy that p53-K370me2-mediated 53BP1 interactions were speculated to fine tune p53 function by potentially enhancing p53-RE interactions in a manner counteracted by the lysine-demethylase LSD1 (Huang et al., 2007). Indeed, this is in line with our observations that 53BP1 protein complexes have a role in stimulating p53-RE interactions (see below).

Lastly, we establish a role for USP28-53BP1 cooperation in regulating p53-dependent transcription, attributing function to protein interactions first described a decade ago (Zhang et al., 2006). Our data provide a mechanistic explanation for USP28’s reported contribution to apoptotic responses via a Chk2-p53-PUMA pathway, and in agreement we detected defects in the p53-dependent activation of PUMA and other genes in *USP28Δ* cell lines (Figures S6C and 5E). Importantly, the binding of p53 to p53-REs across multiple genes was consistently diminished under both basal and stimulated conditions in 53BP1- and USP28-deficient cell lines to similar levels, confirming the positive regulation of p53 DNA binding to be a central role for 53BP1-USP28 complexes. While we have not yet defined the precise mechanism in which 53BP1-USP28 complexes stimulate p53 DNA binding, our data show that the DUB activity of USP28 is required in this context. Given that p53 activity is quenched via MDM2-dependent p53 ubiquitination (Brooks and Gu, 2006, Toledo and Wahl, 2006), it is tempting to speculate that a 53BP1-dependent targeting of USP28 into p53-protein complexes might counteract such events. In this light, it is interesting that HAUSP (USP7), a DUB originally proposed to counteract MDM2-dependent p53 ubiquination (Li et al., 2002), has since been found to target MDM2 as its prime substrate, reconciling contradictory observations that p53 was stabilized in cells of diminished HAUSP (Cummins et al., 2004, Li et al., 2004). Thus, the existence of alternative DUBs that target p53 has been speculated (Brooks and Gu, 2006), and USP28 represents an attractive candidate. While undoubtedly important for p53 turnover, not all of MDM2’s inhibitory roles on p53 activity can be explained at this level, and MDM2 has been proposed to inhibit p53 activities in other ways (Brooks and Gu, 2006). A ubiquitination-dependent inhibition of p53 DNA-binding activity could represent an additional means by which p53 activity can be down-tuned by MDM2. If so, the 53BP1-USP28-p53 axis identified here could represent a means by which such inhibition can be reversed, rapidly enhancing p53’s transcriptional potential upon release from inhibitory MDM2 complexes. In this regard, it is noteworthy that while 53BP1 readily co-immunoprecipitated p53 from cell lysates, we were unable to detect 53BP1 at p53-REs by ChIP. We therefore hypothesize that 53BP1-USP28 complexes interact with nucleoplasmic p53 pools, where they function to prime p53 DNA-binding activity. Such a notion would be consistent with structural observations that showed common p53 DBD residues mediate both 53BP1 and DNA binding and the accompanying prediction that the two events could not occur simultaneously (Joo et al., 2002). While further work will be needed to define the exact mechanism of 53BP1-USP28-p53 interplay, here we provide an integrated model in which p53’s transcription-regulating activities are actively enhanced by 53BP1-USP28 protein complexes. This would be consistent with a putative cooperation between p53, 53BP1, and USP28 in tumor suppression. Indeed, our study paves the way for investigations to disentangle 53BP1’s roles during p53 and DNA repair regulation to define the relative importance of each function in counteracting cancer.

## Experimental Procedures

### Cell Lines, Cell Culture, and Genome Editing

All MCF-7 human breast adenocarcinoma cell lines were cultured in DMEM supplemented with 10% FBS, Pen-Strep, and 2 mM L-Glutamine at 37°C in 5% CO_2_. *p53Δ*, *53BP1Δ*, *53BP1*^*ΔBRCT*^, and *USP28Δ* MCF-7 were generated using the CRISPR-Cas9 system. Briefly, gene-specific gRNAs (sequences in Supplemental Experimental Procedures) were cloned in a modified pX330 vector (Addgene #42230) containing a puromycin resistance cassette. MCF-7 transfected (Fugene HD, Promega) with pX330-puro were enriched by puromycin pulse selection (48 hr, 2 μg/ml), and isogenic clones were isolated by limiting dilution. The presence of gene-disrupting indels in edited cell lines was confirmed by Sanger sequencing, and the ablation of protein production was assessed by immunoblotting and indirect immunofluorescence. Stable-complemented cell lines were generated by lentivirus-mediated transduction, using viral supernatants harvested from 293T cells co-transfected with third generation packaging vectors and pLenti-PGK-PURO-DEST (Addgene #19068) or pHAGE-N-FLAG-HA DEST vectors containing cloned transgene inserts.

### Nutlin-3 Sensitivity Assays

Cells seeded in triplicate at 1.25 × 10^4^ cells/well in 6-well plates were untreated or treated with 4 μM (±)-Nutlin-3 (Cayman Chemicals) 16 hr later. 7 days after treatment, cells were fixed and stained using crystal violet solution (0.5% [w/v] in 20% methanol). For quantification, bound crystal violet was dissolved in 10% (v/v) acetic acid, and absorbance of 1:50 dilutions was measured at 595 nm.

### Immunoprecipitation

Cells lyzed in Benzonase Lysis Buffer (20 mM HEPES [pH 7.9], 40 mM KCl, 2 mM MgCl_2_, 12% glycerol, 0.5% CHAPS, 50 U/ml Benzonase [Novagen], 0.05% [v/v] phosphatase inhibitors [Sigma-Aldrich] and protease inhibitors [Roche]) were supplemented with KCL to a 450 mM final concentration and gently mixed for 30 min at 4°C. Clarified lysates were then cassette dialyzed (Slide-A-Lyzer, Thermo Fisher Scientific) in dialysis buffer (20 mM HEPES [pH 7.9], 100 mM KCl, 0.2 mM EDTA, 10% Glycerol, 0.5 mM DTT, 0.5 mM PMSF, 5 mM NaF, 10 mM β-glycerolphosphate). Flag-HA-53BP1 or endogenous p53 complexes were purified from 1–2 mg total protein using anti-FLAG M2 magnetic resin (Sigma-Aldrich) or p53 DO-1 antibody (Santa Cruz Biotechnology) coupled to protein G Dynabeads (Invitrogen). Protein-bead complexes washed extensively in dialysis buffer were either boiled in Laemmli buffer or eluted in 3× Flag peptide (Sigma-Aldrich).

### Chromatin Immunoprecipitation

ChIP experiments were performed from 30–50 μg MCF-7 chromatin essentially as previously described (Chapman et al., 2013). Briefly, chromatin was immunoprecipitated using 3 μg anti-p53 (DO-1, Santa Cruz), 1.5 μg anti-RNAP2 CTD (phospho-S5; Clone 4H8), 2.5μg Anti-Histone H3 (acetyl K9) (ab4441, Abcam), or 2.5 μg anti-acetyl-histone H4 (06-598, Millipore) antibody coupled to 25 μl Protein-A/G Dynabeads (Life Technologies). DNA quantities recovered in control IgG ChIP experiments were consistently below the detectable range. Relative quantities of ChIP-enriched DNA were calculated relative to total input chromatin by qPCR in triplicate on a CFX96 Real-Time Analyzer (Bio-Rad) using Quantifast SYBR Green reagent (QIAGEN) and locus-specific primer pairs (sequences in Supplemental Experimental Procedures).

## Author Contributions

J.R.C. conceived and supervised the study and wrote the manuscript. J.R.C. and R.C.-M. designed the experiments. R.C.-M. performed the majority of the experiments with assistance from C.O., N.G., S.S., and J.R.C. H.E.L. analyzed all NGS datasets.

## Accession Numbers

The accession number for RNA-seq data reported in this paper is NCBI GEO: GSE84986.

## Supplemental Information

Document S1. Supplemental Experimental Procedures, Figures S1–S7, and Table S1

Document S2. Article plus Supplemental Information
