# Supplementary material for: 53BP1 Integrates DNA Repair and p53-Dependent Cell Fate Decisions via Distinct Mechanisms
Source: Mol Cell. 2016 Oct 6;64(1):51–64. doi: 10.1016/j.molcel.2016.08.002 (PMC5065530; doi:10.1016/j.molcel.2016.08.002)
Supplement: Document S1. Supplemental Experimental Procedures, Figures S1–S7, and Table S1 [file mmc1.pdf]

Molecular Cell, Volume *64*

Supplemental Information

53BP1 Integrates DNA Repair and p53-Dependent Cell Fate Decisions via Distinct Mechanisms

Raquel Cuella-Martin, Catarina Oliveira, Helen E. Lockstone, Suzanne Snellenberg, Natalia Grolmusova, and J. Ross Chapman

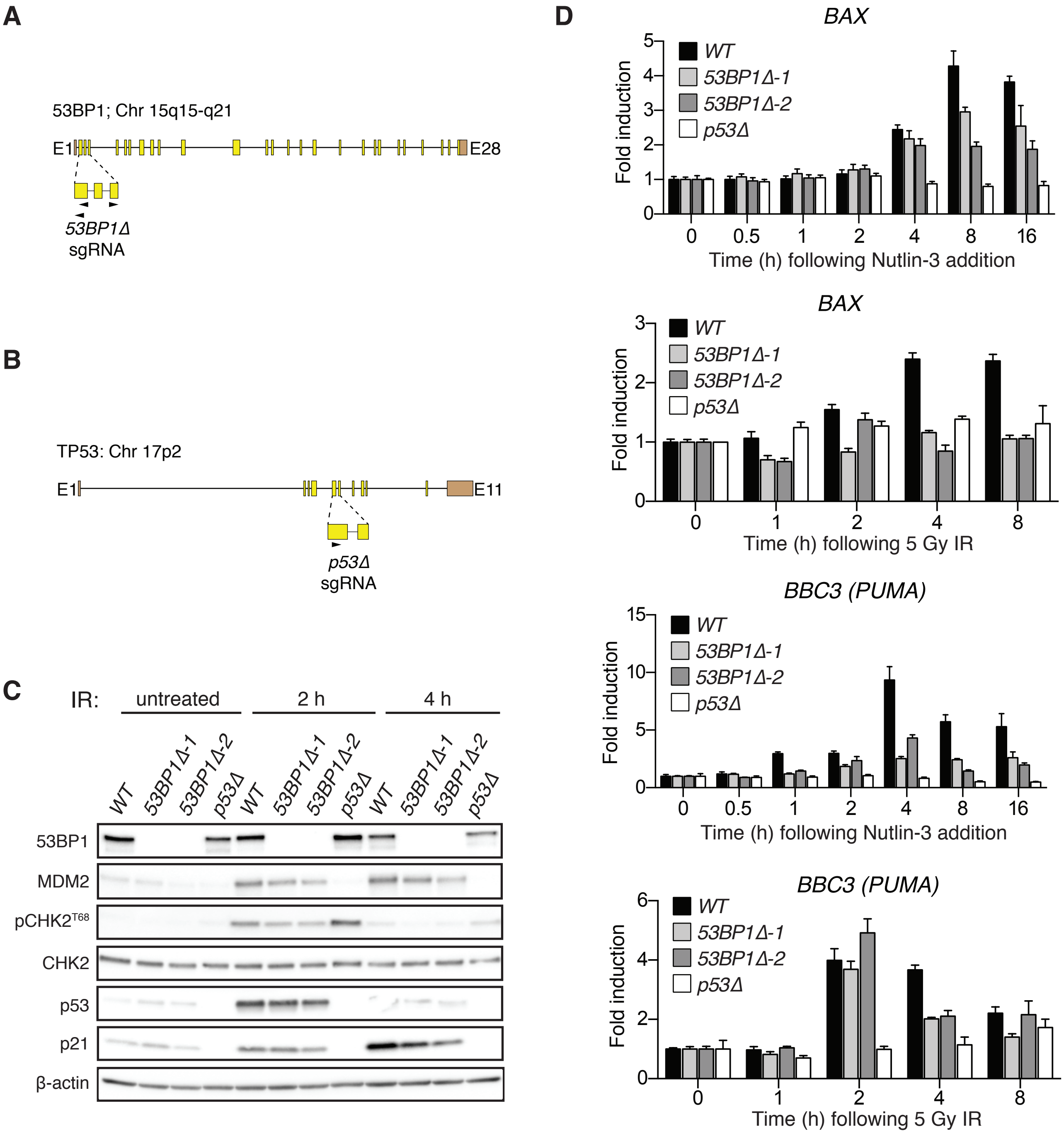
A
53BP1; Chr 15q15-q21
E1
E28
53BP1Δ
sgRNA
B
TP53: Chr 17p2
E1
E11
p53Δ
sgRNA
C
IR:
untreated
2 h
4 h
WT
53BP1Δ-1
53BP1Δ-2
p53Δ
53BP1
MDM2
pCHK2T68
CHK2
p53
p21
β-actin
D
BAX
Fold induction
WT
53BP1Δ-1
53BP1Δ-2
p53Δ
Time (h) following Nutlin-3 addition
BAX
Time (h) following 5 Gy IR
BBC3 (PUMA)
Time (h) following Nutlin-3 addition
BBC3 (PUMA)
Time (h) following 5 Gy IR

Figure S1

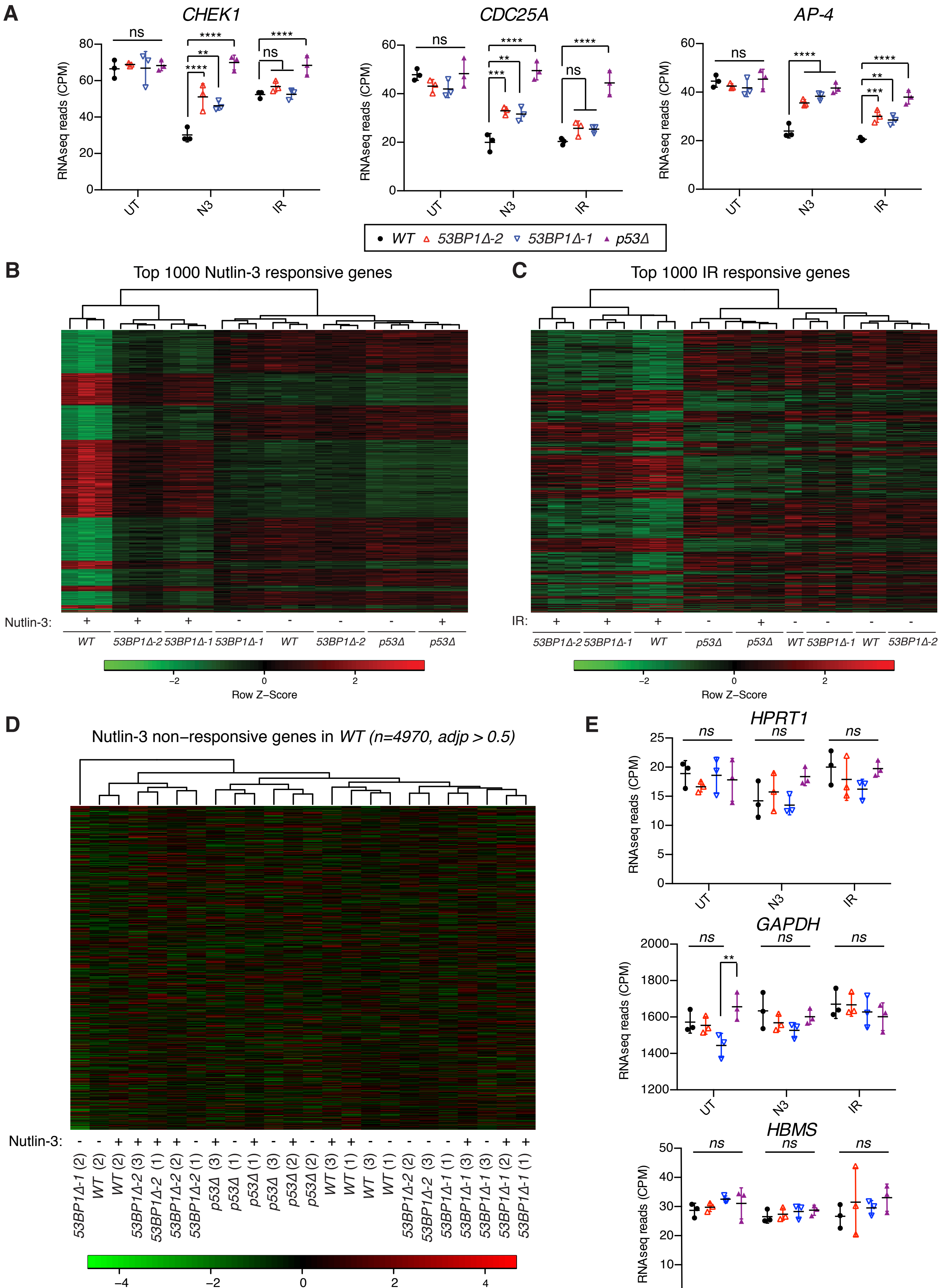
A
CHEK1
CDC25A
AP-4
RNAseq reads (CPM)
UT
N3
IR
ns
****
**
***
• WT ▵ 53BP1Δ-2 ▿ 53BP1Δ-1 ▴ p53Δ
B
Top 1000 Nutlin-3 responsive genes
Nutlin-3:
WT
53BP1Δ-2
53BP1Δ-1
p53Δ
Row Z-Score
C
Top 1000 IR responsive genes
IR:
D
Nutlin-3 non-responsive genes in WT (n=4970, adjp > 0.5)
Row Z-score
E
HPRT1
GAPDH
HBMS

Figure S2

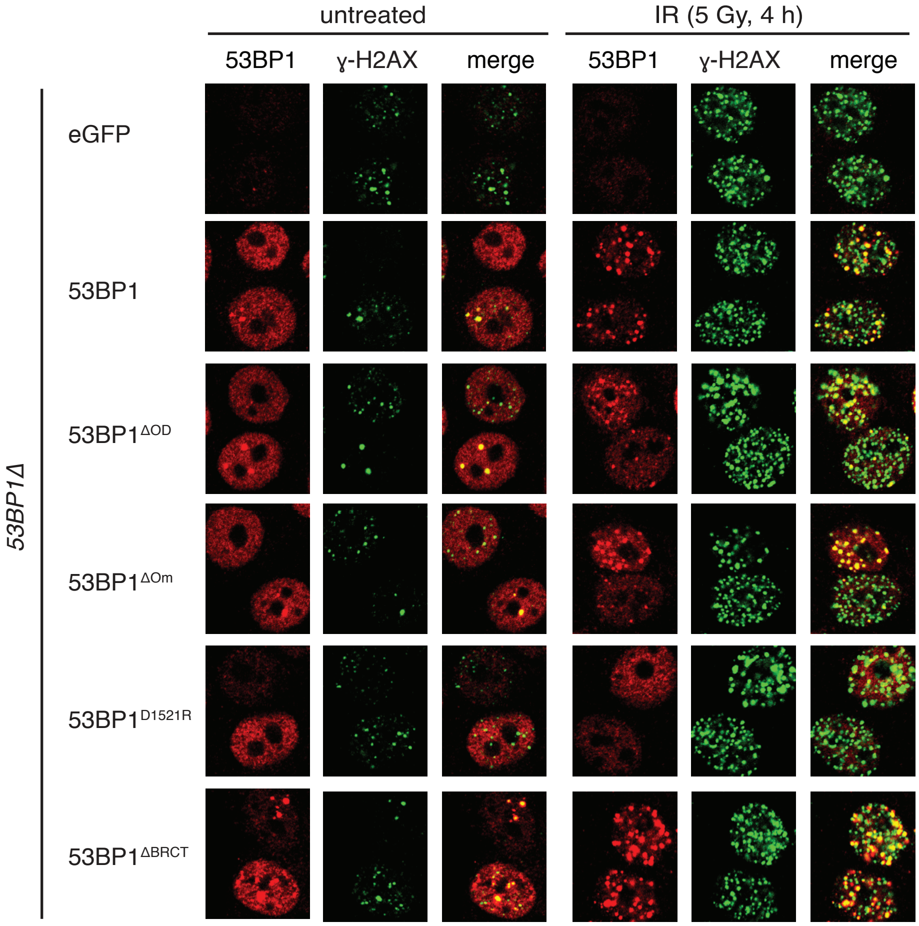
untreated
IR (5 Gy, 4 h)
53BP1
γ-H2AX
merge
53BP1
γ-H2AX
merge
53BP1Δ
eGFP
53BP1
53BP1ΔOD
53BP1ΔOm
53BP1D1521R
53BP1ΔBRCT

Figure S3

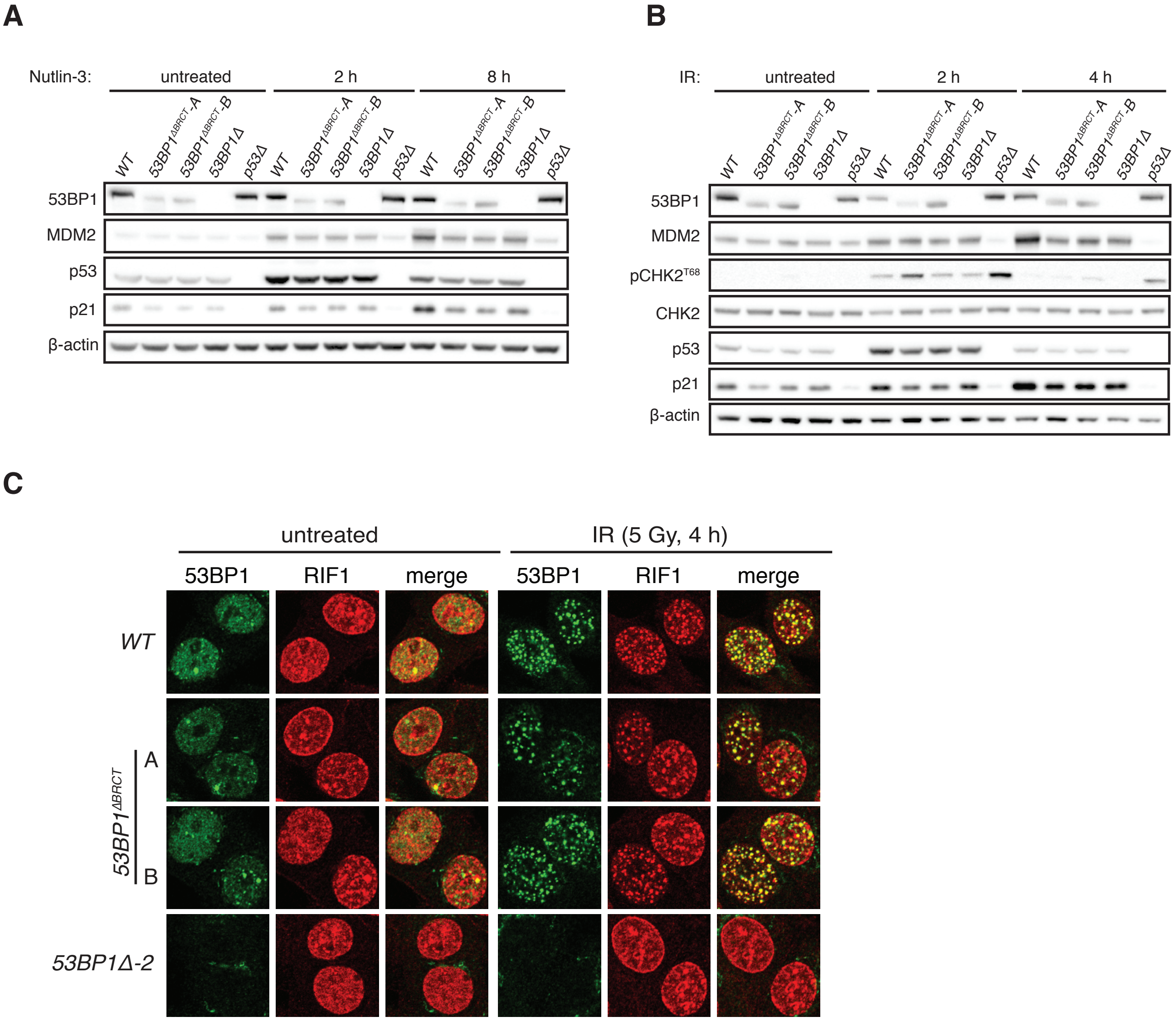
A
Nutlin-3:
untreated
2 h
8 h
WT
53BP1ΔBRCT-A
53BP1ΔBRCT-B
53BP1Δ
p53Δ
53BP1
MDM2
p53
p21
β-actin
B
IR:
untreated
2 h
4 h
53BP1
MDM2
pCHK2T68
CHK2
p53
p21
β-actin
C
untreated
IR (5 Gy, 4 h)
53BP1
RIF1
merge
WT
53BP1ΔBRCT
A
B
53BP1Δ-2

Figure S4

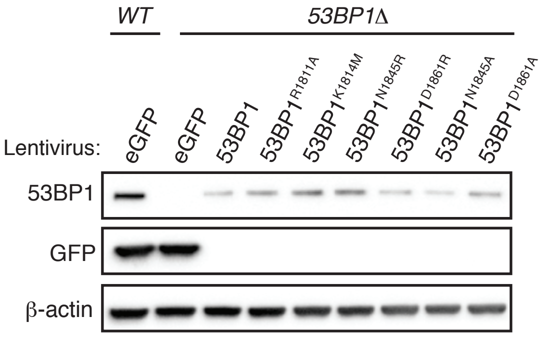
WT
53BP1Δ
Lentivirus:
eGFP
eGFP
53BP1
53BP1R1811A
53BP1K1814M
53BP1N1845R
53BP1D1861R
53BP1N1845A
53BP1D1861A
53BP1
GFP
β-actin

Figure S5

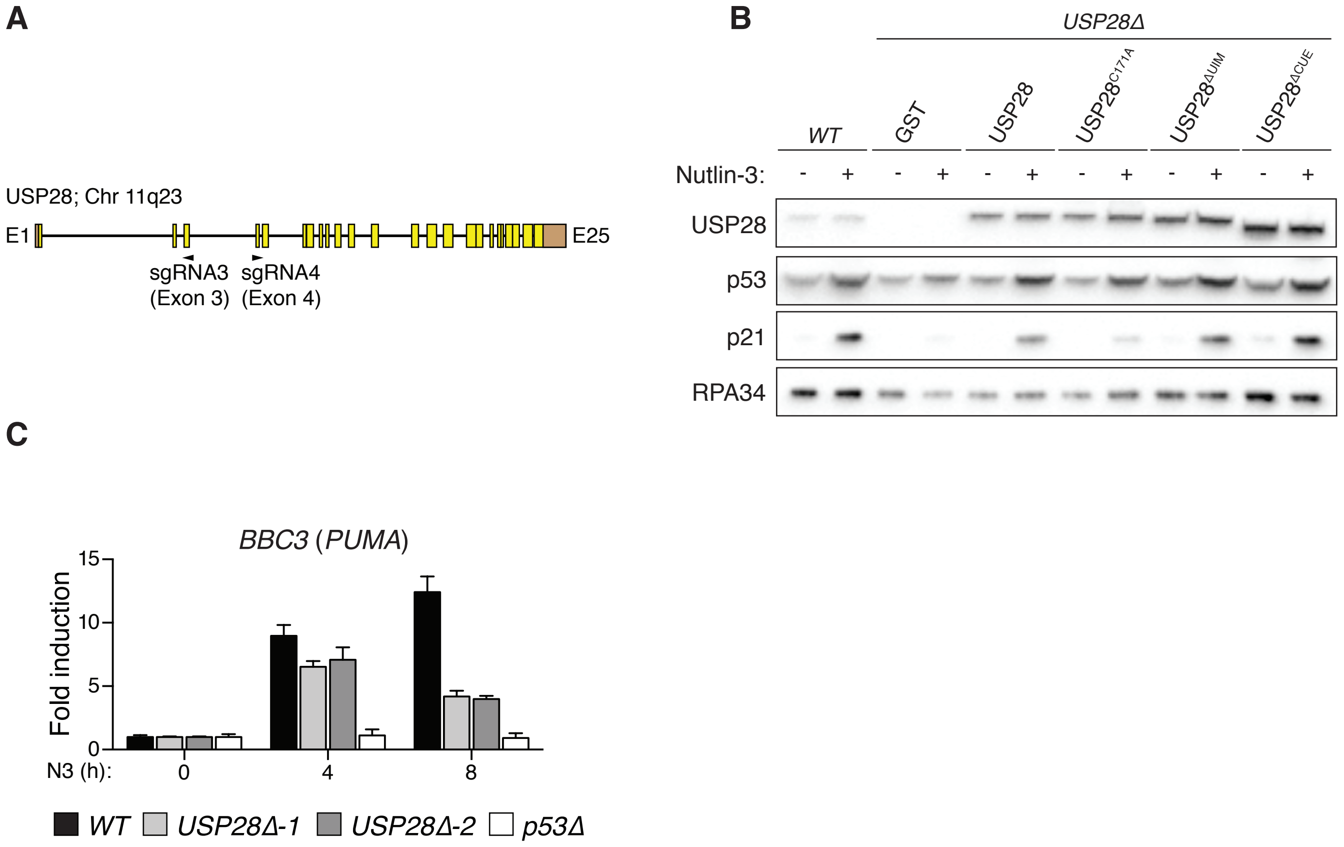
A
USP28; Chr 11q23
E1
E25
sgRNA3 (Exon 3)
sgRNA4 (Exon 4)
B
USP28Δ
WT
GST
USP28
USP28C171A
USP28ΔUIM
USP28ΔCUE
Nutlin-3:
USP28
p53
p21
RPA34
C
BBC3 (PUMA)
Fold induction
N3 (h):
0
4
8
WT
USP28Δ-1
USP28Δ-2
p53Δ

Figure S6

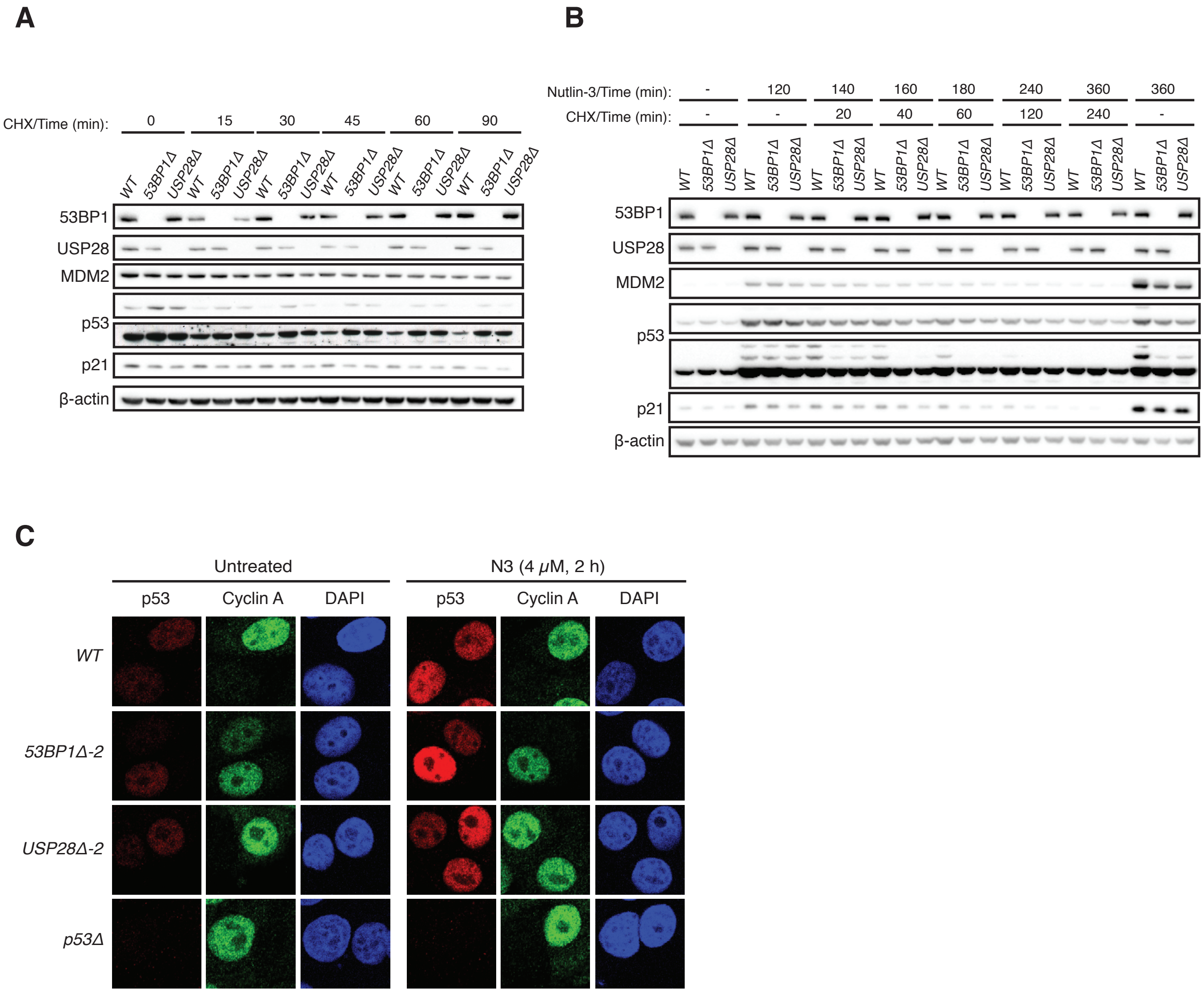
A
CHX/Time (min):
0
15
30
45
60
90
WT
53BP1Δ
USP28Δ
53BP1
USP28
MDM2
p53
p21
β-actin
B
Nutlin-3/Time (min):
120
140
160
180
240
360
CHX/Time (min):
20
40
60
120
240
C
Untreated
N3 (4 μM, 2 h)
p53
Cyclin A
DAPI
WT
53BP1Δ-2
USP28Δ-2
p53Δ

Figure S7

SUPPLEMENTARY FIGURE LEGENDS

Supplementary Figure S1, **related to Figure 1**. **53BP1 is required for optimal p53-dependent transactivation events.**

Schematic representations of the human *53BP1* **(A)** and *TP53* **(B)** loci, illustrating the hybridization sites of the gRNAs selected for the generation of *53BP1*Δ and *p53*Δ cell-lines in this study (gRNA sequences in Supplementary Table S1). **(C)** Immunoblot analysis of lysates prepared from untreated or IR-treated (5 Gy) cells of indicated genotype. **(D)** 53BP1-loss impairs IR-induced p53-dependent transactivation events. Total RNA was purified from cells treated with N3 (4 µM) or IR (5 Gy) and monitored for the abundance of indicated p53-responsive transcripts by RT-qPCR. Data is representative of two independent experiments, mean ± SD.

Supplementary Figure S2, **related to Figure 1**. **53BP1 is an enhancer of p53-dependent transcriptional programs.**

(A) 53BP1 is required for p53-dependent transcriptional repression events. Representative p53-responsive transcripts from three RNA-seq replicates. Total RNA was sequenced from indicated MCF-7 lines following N3 (4 µM, 8 h), IR (5 Gy, 4 h) or control treatments. CPM, counts per million; *ns*, non significant; *$p<0.05$; **$p<0.01$; ***$p<0.001$; ****$p<0.001$ (two-way ANOVA). Bars represent mean ± SD. **(B)**, **(C)** RNA-seq results and reproducibility across biological replicates. Heatmaps showing relative expression levels for each independent biological replicate of each condition for the top 1000 treatment-responsive genes, in respect to the untreated control samples. Unsupervised hierarchical clustering was used to cluster the samples based on expression levels of the respective set of 1000 genes for N3 and IR treatments. **(D)** Normal expression of Nutlin-3 non-responsive genes in *53BP1*Δ and *p53*Δ MCF-7. Heatmaps showing relative expression levels for each independent biological replicate of each condition for the 4970 treatment non-responsive genes in *WT* MCF-7, in respect to the untreated control samples. Unsupervised hierarchical clustering was used to cluster the samples based on expression levels of the respective set of 4970 genes for N3 and IR treatments. **(E)** As in **A**, but for three commonly employed reference transcripts.

Supplementary Figure S3, related to Figure 2. Expected nuclear localization patterns of N3-response defective 53BP1 mutants.

The localization and IR-dependent recruitment patterns of the indicated 53BP1 mutant proteins was analyzed in stably transduced *53BP1*Δ MCF-7 lines following mock or IR (5Gy 4 h) treatment. Cells were fixed, immunostained with indicated antibodies, and then processed by indirect immunofluorescence.

Notably, 53BP1 oligomerization mutant proteins formed residual IRIF, consistent with a previous report (Lottersberger et al., 2013).

Supplementary Figure S4, related to Figure 3. 53BP1 dependent p53 regulation and DSB repair activities are distinct and separable.

(A), (B) *53BP1*$^{\Delta BRCT}$ cells show reduced MDM2 and p21 induction following N3 and IR treatments. Western blot analysis of lysates prepared from indicated cell-lines following treatment with 4 μM N3 (A) or 5 Gy IR (B) relative to untreated control samples. **(C)** Unlike *53BP1*Δ cells, *53BP1*$^{BRCT\Delta}$ cells are proficient in supporting RIF1 recruitment into IR-induced foci. Cells mock-treated or irradiated (5 Gy, 4 h), were fixed, immunostained with indicated antibodies, and processed by indirect immunofluorescence.

Supplementary Figure S5, related to Figure 4. The 53BP1 BRCT domain mediates bivalent interactions with p53 and USP28.

Western blot showing stable expression of indicated WT and 53BP1 BRCT mutants in lentivirus-transduced *53BP1Δ* cells.

Supplementary Figure S6, related to Figure 5. USP28 is a novel component of the p53-53BP1 axis.

(A) Schematic representation of the human *USP28* locus depicting the hybridization sites for each gRNA used to generate *USP28Δ* cell-lines (gRNA sequences in supplementary Table S1). **(B)** USP28 catalytic activity is required for its p53-regulatory role. Indicated WT and mutant USP28 transgenes were stably expresses in *USP28Δ* cells following lentivirus-mediated transduction. Lysates were prepared from cells following treatment with N3 (4 μM, 8 h), and immunoblotted with indicated antibodies. **(C)** USP28-loss impairs N3-induced p53-dependent PUMA transactivation. Total RNA was purified from cells treated with N3 (4 μM) and monitored for the abundance of BBC3 (PUMA) transcript for the indicated times. Data is representative of two independent experiments, mean ± SD.

Supplementary Figure S7, related to Figure 7. Normal p53 stability and localization in *53BP1*Δ and *USP28*Δ cells.

(A) Steady-state p53 half-life in WT, *53BP1*Δ and *USP28*Δ cells. p53 half-life analysis was performed in time-course experiments following cyclohexamide addition to halt *de novo* protein synthesis. Cell lysates prepared at indicated time-points following cycloheximide addition were immunoblotted with indicated antibodies. **(B)** p53 half-life in N3-treated WT, *53BP1*Δ and *USP28*Δ cells. Similar to B, except cycloheximide was added 2 h following p53-activation by N3-treatment. **(C)** Normal p53 nuclear localization in WT, *53BP1*Δ and *USP28*Δ cells. Cells were treated with 4 μM N3 for 2 h or left untreated,

were fixed and immunostained with the monoclonal p53 DO-7 antibody. Cyclin A-counterstaining enabled discrimination of G1 (Cyclin A negative) and S/G2 (Cyclin A positive) cell populations, and provided no evidence for cell-cycle-dependent defects in p53 localization.

	**Gene #**	**Nutlin-3**	**IR**
WT	Activated	3559	1043
	Repressed	3318	1343
53BP1Δ-1	Activated	1143	497
	Repressed	1096	686
53BP1Δ-2	Activated	1366	551
	Repressed	1624	767
p53Δ	Activated	10	6
	Repressed	3	19

Supplementary Table S1, related to Figure 1. Number of genes showing significant changes (*adjusted p-value <0.05*) in RNA-seq experiments upon N3 and IR treatments with respect to the corresponding untreated control.

EXTENDED EXPERIMENTAL PROCEDURES

Protein analysis: SDS/PAGE-Western Blot

Whole cell protein extracts were isolated using Benzonase buffer [25 mM Tris (pH 8.0), 40 mM NaCl, 0.05% SDS, 2 mM $MgCl_2$, 10 U/ml Benzonase (Sigma-Aldrich), 0.05% (v/v) phosphatase inhibitor cocktail 3 (P5726; Sigma-Aldrich) and protease inhibitors (Complete, Roche)], diluted in 3X Laemmli buffer and boiled. SDS-PAGE was performed using NuPAGE® Novex® (Life Technologies) or Criterion® (Bio-Rad) gradient acrylamide gels before transfer onto 0.45 µM nitrocellulose membranes (Life Technologies). Primary antibodies used in this study are listed below. Proteins were detected using HRP-conjugated secondary antibodies and enhanced chemiluminescence (Clarity, Bio-Rad). Signals were acquired digitally on a Gel Doc™ XR system (Bio-Rad).

Immunofluorescence assays

Cells were seeded in coverslips in 6-well plates at a cell density of $4x10^5$ cells/well. After treatments, cells were fixed in a 2% *p*-formaldehyde solution and permeabilized with 0.2% (v/v) Triton-X100 in PBS. Coverslips were blocked in 3% (w/v) BSA/0.1% (v/v) Triton-X100 in PBS prior to sequential incubations with primary (listed below) and secondary antibodies (Alexa Fluor 488, 594 and 648; Molecular Probes). Images were captured in a confocal scanning microscope (LSM 510 Meta, Zeiss).

Clonogenic IR survival assay

The sensitivity of cells plated on 10 cm dishes in triplicate and exposed to indicated X-ray doses (CellRad, Faxitron), was assessed relative to non-irradiated control plates. Fourteen days after treatment, plates were stained using crystal violet dye and colonies counted.

Real time quantitative PCR (RT-qPCR)

Gene expression was determined by RT-qPCR. Trizol/chloroform-extracted total RNA was further purified with the RNeasy Mini Kit (Qiagen) incorporating a DNaseI step (Qiagen) to remove DNA contamination. cDNA was generated from 1 µg of total RNA using the iScript® cDNA Synthesis Kit (Bio-Rad). qPCR was carried out using QuantiFast SYBR Green Master Mix (Qiagen) and transcript-specific primer pairs (sequences listed below). PCR reactions were analysed on a CFX96® Real Time analyzer (Bio-Rad) with the following conditions: enzyme activation-5 min 95 ^oC; 40 cycles denaturation-10 s 95 ^oC annealing/extension-30 s 60 ^oC; final melting curve-15s 65 ^oC, 15 s 95 ^oC. Cycle threshold values (C_T) were used to perform quantification and analysis using CFX Manager software (Bio-Rad).

Hypoxanthine-guanine phosphoribosyl-transferase 1 (*HPRT1*) was used as housekeeping gene for normalization. All values were presented as fold-changes compared to the appropriate control.

Whole transcriptome analysis: RNA-seq

Libraries prepared from ribosomal-RNA depleted total RNA isolates (Ribo-Zero rRNA removal kit, Illumina) were subjected to RNA-seq analysis. Results presented are based on three biological experimental replicates for each condition and genotype. Briefly, sample preparation was carried out according to Illumina guidelines using in-house adapters for library preparation (Lamble et al., 2013). Library quantification and quality control was performed using Picogreen and Tapestation measurements, and equimolar quantities of each library pooled into a 36-plex. 36-plex pools were sequenced (100 bp paired-end reads) across two lanes of a HiSeq4000 sequencer (Illumina). Following subtraction of low-quality reads and duplicate reads (Picard Tools MarkDuplicates), between 10-15 million high-quality reads per sample were analyzed and aligned to the human reference genome (GRCh37), using TopHat2 (Kim et al., 2013). Counts for Ensembl-annotated genes were summarised from the mapped reads, and filtered to exclude genes with fewer than 10 reads on average per sample. Analysis for differential expression was performed using the edgeR package (Robinson et al., 2010). All raw RNA-sequencing datasets generated in this study will be archived and made publically available at the European Nucleotide Archive upon publication.

Immunoprecipitation

Cells initially lyzed in Benzonase Lysis Buffer [20 mM HEPES (pH 7.9), 40 mM KCl, 2 mM $MgCl_2$, 12% glycerol, 0.5% CHAPS, 50 U/ml Benzonase (Novagen), 0.05 % (v/v) phosphatase inhibitors (P0044 and P5726; Sigma-Aldrich) and protease inhibitors (Complete, Roche)], were supplemented with KCL to a 450 mM final concentration and gently mixed for 30 min at 4˚C. Following clarification by centrifugation, lysates were then cassette dialyzed (Slide-A-Lyzer™ MINI, Thermo Fisher Scientific) into dialysis buffer [20 mM HEPES (pH 7.9), 100 mM KCl, 0.2 mM EDTA, 10 % Glycerol, 0.5 mM DTT, 0.5 mM PMSF, 5 mM NaF, 10 mM b-glycerolphosphate]. Flag-HA-53BP1 or endogenous p53 complexes were purified from 1-2 mg total protein using anti-FLAG® M2 magnetic resin (Sigma-Aldrich) or p53 DO-1 antibody (Santa Cruz Biotechnology) coupled to protein G Dynabeads (Invitrogen). Magnetically purified protein-bead complexes washed extensively in dialysis buffer were either boiled in Laemmli buffer or eluted in 3X Flag peptide (Sigma-Aldrich) according to manufacturers instructions.

G1/S checkpoint analysis

The G1/S checkpoint was assessed by BrdU incorporation. Briefly, cells were synchronised in G0 by serum starvation for 24 h and further released in medium containing 0.25 µg/ml nocodazole to prevent G2/M-phase cells recycling. 4 h post-release, G1-phase cells were treated with mock- or 4 Gy irradiation. Twelve and 18 h later, cells were then pulsed for 30 min with 10 µM BrdU, collected and fixed overnight in ice-cold 70% ethanol. DNA denaturation was performed using a solution of 0.2 mg/ml of pepsin (Sigma-Aldrich) in 2 M HCl. BrdU was detected using an anti-BrdU-FITC conjugated antibody (AbD Serotec) and a solution of PI/RNaseA (10 µg/ml and 0.1 mg/ml respectively) was used for total DNA staining. Cells were analysed in an Attune NxT flow cytometer (Life Technologies) and data processed using FlowJo software (Three Star Inc).

Statistical methods

Prism 6 software (GraphPad Software Inc.) was typically used for statistical analysis of datasets, with the exception of the RNAseq analyses that were performed using R (www.r-project.org).

CRISPR-Cas9 guide-RNAs (gRNAs) used to generate knockout and 53BP1$^{\Delta BRCT}$ cell lines.

Target gene/Name		Sequence (5'-3')	Description
TP53		gtgcagctgtgggttgattc	Targeted to exon 5, antisense.
53BP1	1	gaatccaactgacttccagt	Targeted to exon 2, antisense
	2	gctgagaatcttcaattatc	Targeted to exon 2, antisense
	3	gaacgaggagacggtaatagt	Targeted to exon 3, sense.
USP28	1	tgtagcaacagtgtcttgac	Targeted to exon 3, antisense.
	2	tgccattgctttgagtctac	Targeted to exon 4, sense.
53BP1	BRCT Nt 1	ctgtgagagtggagacaaca	Targeted to BRCT 1 N-term, antisense.
	BRCT Nt 2	tttgtgagcccctgtgagag	Targeted to BRCT 1 N-term, sense.
	BRCT Ct	ctcattgttggggagagaat	Targeted to BRCT 2 C-term, sense.

53BP1 lentiviral plasmids used throughout this study.

**Name**		**Description**	**Application**
pLenti-PGK-PURO-DEST	eGFP	Control plasmid	N3 survival studies
	GST	Control plasmid	
	53BP1	Full length 53BP1 ORF	
	53BP1$^{\Delta BRCT}$	Deletion of BRCT tandem domain	
	53BP1$^{\Delta OD}$	Deletion of Oligomerisation domain	
	53BP1^{ODm}	Mutation YYVD1258AAAA	
	53BP1^{20AQ}	Mutation of 20 N-terminal S/TQ sites	
	53BP1^{D1521R}	Mutation of the Tudor domain	
	53BP1^{L1619A}	Mutation of the UDR domain	
	53BP1^{R1811A}	Mutation in BRCT P-binding pocket	
	53BP1^{K1814M}	Mutation in BRCT P-binding pocket	
	53BP1^{N1845R}	Mutation in p53 binding interphase	
	53BP1^{N1845A}		
	53BP1^{D1861R}	Mutation in p53 binding interphase	
	53BP1^{D1861A}		
	USP28	Full length USP28 ORF	
	USP28$^{\Delta UBA}$	Deletion of predicted CUE domain (S20-E65)	
	USP28$^{\Delta UIM}$	Deletion of UIM domain (K99-I116)	
	USP28^{C171A}	Catalytic dead USP28 version	
pHAGE-N-FLAG-HA-DEST	eGFP	Control plasmid	Interaction studies (Co-IP)
	53BP1	Full length 53BP1 ORF	
	53BP1$^{\Delta BRCT}$	Deletion of BRCT tandem domain	
	53BP1^{ODm}	Mutation YYVD1258AAAA	
	53BP1^{D1521R}	Mutation of the Tudor domain	
	53BP1^{R1811A}	Mutation in BRCT P-binding pocket	
	53BP1^{K1814M}	Mutation in BRCT P-binding pocket	
	53BP1^{N1845R}	Mutation in p53 binding interphase	
	53BP1^{N1845A}		
	53BP1^{D1861R}	Mutation in p53 binding interphase	
	53BP1^{D1861A}		

Sequences of primer pairs used throughout this study.

**Target gene / locus**		**Sequence (5'-3')**	**Application**
CDKN1A (*p21*)	Fwd	CCTCATCCCGTGTTCTCCTTT	Transcript-specific qRT-PCR primers
	Rev	GTACCACCCAGCGGACAAGT	
BAX	Fwd	CCTTTTCTACTTTGCCAGCAAAC	
	Rev	GAGGCCGTCCCAACCAC	
BBC3 (*PUMA*)	Fwd	CCTGGAGGGTCCTGTACAATCT	
	Rev	GCACCTAATTGGGCTCCATCT	
TP53I3	Fwd	AGGGTGAAGTCCTCCTGAAGGT	
	Rev	GTGGGTCATACTGGCCTTGTCT	
MDM2	Fwd	GGCCTGCTTTACATGTGCAA	
	Rev	GCACAATCATTTGAATTGGTTGTC	
CDKN1A (*p21*) -2965	Fwd	CCGGCCAGTATATATTTTTAATTGAGA	Locus-specific ChIP primers
	Rev	AGTGGTTAGTAATTTTCAGTTTGCTCAT	
CDKN1A (*p21*) -2283	Fwd	AGCAGGCTGTGGCTCTGATT	
	Rev	CAAAATAGCCACCAGCCTCTTCT	
CDKN1A (*p21*) -1391	Fwd	CTGTCCTCCCCGAGGTCA	
	Rev	ACATCTCAGGCTGCTCAGAGTCT	
CDKN1A (*p21*) -20	Fwd	TATATCAGGGCCGCGCTG	
	Rev	GGCTCCACAAGGAACTGACTTC	
CDKN1A (*p21*) +507	Fwd	CCAGGAAGGGCGAGGAAA	
	Rev	GGGACCGATCCTAGACGAACTT	
CDKN1A (*p21*) +4001	Fwd	AGTCACTCAGCCCTGGAGTCAA	
	Rev	GGAGAGTGAGTTTGCCCATGA	
CDKN1A (*p21*) +8566	Fwd	CCTCCCACAATGCTGAATATACAG	
	Rev	AGTCACTAAGAATCATTTATTGAGCACC	
CDKN1A (*p21*) +11443	Fwd	TCTGTCTCGGCAGCTGACAT	
	Rev	ACCACAAAAGATCAAGGTGAGTGA	
MDM2 5'	Fwd	GGGCTATTTAAACCATGCATTTTC	
	Rev	GTCCGTGCCCACAGGTCTA	
MDM2 3'	Fwd	CTTTCTCGAGGAGGCAGGTTT	
	Rev	GCTCAACCCTAGGCGCTATTC	
GADD45A p53-RE	Fwd	GCCTTTGTCCGACTAGAGTGT	
	Rev	GGATCTCTTCCGCTGCTG	
FAS p53-RE	Fwd	GCACCGAAGCAGTGGTTAAG	
	Rev	GCCTCCAGAAGCTCATTCAG	

Antibodies used in this study.

Target	Manufacturer	Application
53BP1	Novus Biological (NB100-304)	WB, Immunofluorescence
	Novus Biological (NB100-305)	WB
	Millipore (clone B13)	WB, Immunofluorescence
MDM2	Santa Cruz Biotechnology (clone SMP14)	WB
p53	Dako (clone DO-7)	WB, Immunofluorescence
	Santa Cruz Biotechnology (clone DO-1)	Immunoprecipitation, ChIP
p21	BD Transduction Laboratories	WB
CHK2	Millipore (clone 7)	WB
$pCHK2^{T68}$	Cell signalling (clone C13C1)	WB
USP28	Abcam (EPR4249)	WB
HA-11	Covance Research (Clone 16B12)	WB
β-actin	Sigma-Aldrich	WB
α-tubulin	Sigma-Aldrich (Tat-1)	WB
RIF1	Bethyl Laboratories (A300-569A)	Immunofluorescence
ɣ-H2AX	Millipore	Immunofluorescence
Cyclin A	Clone E23.1, a gift from Julian Gannon, The Francis Crick Institute	Immunofluorescence

Supplementary References

Kim, D., Pertea, G., Trapnell, C., Pimentel, H., Kelley, R., Salzberg, S.L. (2013). TopHat2: accurate alignment of transcriptomes in the presence of insertions, deletions and gene fusions. Genome Biol *14*, R36.

Lamble, S., Batty, E., Attar, M., Buck, D., Bowden, R., Lunter, G., Crook, D., El-Fahmawi, B., Piazza, P. (2013). Improved workflows for high throughput library preparation using the transposome-based nextera system. BMC Biotech *13*, 104.

Lottersberger, F., Bothmer, A., Robbiani, D.F., Nussenzweig, M.C., and De Lange, T. (2013). Role of 53BP1 oligomerization in regulating double-strand break repair. Proc. Natl. Acad. Sci. U.S.A. *110*, 2146–2151.

Robinson, M.D., McCarthy, D.J., Smyth, G.K. (2010). edgeR: a Bioconductor package for differential expression analysis of digital gene expression data. Bioinformatics *26*, 139-140.
