# Supplementary material for: 53BP1 Integrates DNA Repair and p53-Dependent Cell Fate Decisions via Distinct Mechanisms
Source: Mol Cell. 2016 Oct 6;64(1):51–64. doi: 10.1016/j.molcel.2016.08.002 (PMC5065530; doi:10.1016/j.molcel.2016.08.002)
Supplement: Document S2. Article plus Supplemental Information [file mmc2.pdf]

Molecular Cell

Article

53BP1 Integrates DNA Repair and p53-Dependent Cell Fate Decisions via Distinct Mechanisms

Graphical Abstract

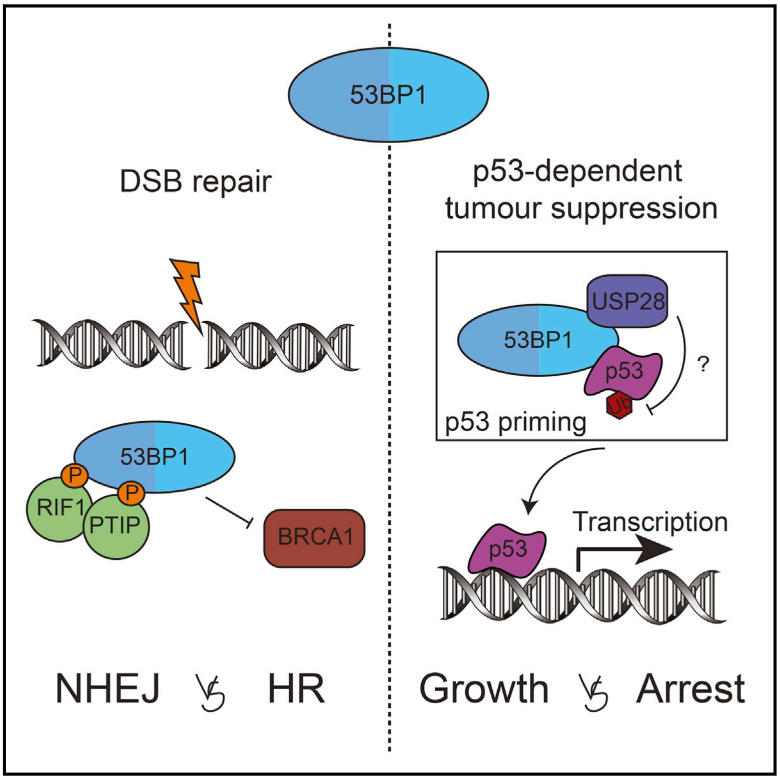

Authors

Raquel Cuella-Martin,
Catarina Oliveira, Helen E. Lockstone,
Suzanne Snellenberg,
Natalia Grolmusova, J. Ross Chapman

Correspondence

rchapman@well.ox.ac.uk

In Brief

53BP1 was first identified as p53 binding protein 1, yet the function of the p53-53BP1 interaction was unclear. Cuella-Martin et al. show that 53BP1 bridges interactions between p53 and the deubiquitinating enzyme USP28, promoting p53-DNA interactions to globally enhance p53-dependent transcriptional programs.

Highlights

- 53BP1 enhances genome-wide p53-dependent transcriptional responses
- 53BP1's DNA repair and p53-regulatory roles are distinct and separable
- Regulation requires 53BP1 oligomerization and BRCT domain interactions with p53 and USP28
- 53BP1-USP28 complexes function to stimulate p53 DNA-binding activity

Accession Numbers

GSE84986

Cuella-Martin et al., 2016, Molecular Cell *64*, 51–64
October 6, 2016 © 2016 The Authors. Published by Elsevier Inc.
http://dx.doi.org/10.1016/j.molcel.2016.08.002

Molecular Cell
Article

CellPress

53BP1 Integrates DNA Repair and p53-Dependent Cell Fate Decisions via Distinct Mechanisms

Raquel Cuella-Martin,[1] Catarina Oliveira,[1] Helen E. Lockstone,[2] Suzanne Snellenberg,[1] Natalia Grolmusova,[1] and J. Ross Chapman[1,3,*]
[1]Chromatin and Genome Integrity Laboratory, Wellcome Trust Centre for Human Genetics, University of Oxford, Oxford OX3 7BN, UK
[2]Bioinformatics and Statistical Genetics Core, Wellcome Trust Centre for Human Genetics, University of Oxford, Oxford OX3 7BN, UK
[3]Lead Contact
*Correspondence: rchapman@well.ox.ac.uk
http://dx.doi.org/10.1016/j.molcel.2016.08.002

SUMMARY

The tumor suppressor protein 53BP1, a pivotal regulator of DNA double-strand break (DSB) repair, was first identified as a p53-interacting protein over two decades ago. However, its direct contributions to p53-dependent cellular activities remain undefined. Here, we reveal that 53BP1 stimulates genome-wide p53-dependent gene transactivation and repression events in response to ionizing radiation (IR) and synthetic p53 activation. 53BP1-dependent p53 modulation requires both auto-oligomerization and tandem-BRCT domain-mediated bivalent interactions with p53 and the ubiquitin-specific protease USP28. Loss of these activities results in inefficient p53-dependent cell-cycle checkpoint and exit responses. Furthermore, we demonstrate 53BP1-USP28 cooperation to be essential for normal p53-promoter element interactions and gene transactivation-associated events, yet dispensable for 53BP1-dependent DSB repair regulation. Collectively, our data provide a mechanistic explanation for 53BP1-p53 cooperation in controlling anti-tumorigenic cell-fate decisions and reveal these activities to be distinct and separable from 53BP1's regulation of DNA double-strand break repair pathway choice.

INTRODUCTION

Tumor suppression requires the integration of molecular signals that collectively function to safeguard genome integrity, while preventing the inheritance of potentially oncogenic mutations within proliferating cell populations. The transcription factor p53 is an impressive integrator and propagator of anti-tumorigenic cellular signals. Responding to a variety of stress signals that include DNA damage and aneuploidy, p53 regulates the transcription of a multitude of target genes to elicit cell-cycle arrest, apoptosis, senescence, DNA repair, and metabolic responses (Toledo and Wahl, 2006; Vousden and Prives, 2009). p53's importance as a tumor suppressor is well exemplified by estimates that *p53* mutations are founder and/or driver events in around 50% of all cancers. Given that p53 elicits life and death decisions in response to an array of stimuli, it is subject to exquisite control via multiple mechanisms involving an array of interaction partners. One such partner, p53-binding protein 1 (53BP1) was first identified alongside ASPP2 (53BP2) as yeast two-hybrid interactors of the p53 DNA binding domain (DBD) (Iwabuchi et al., 1994). While ASPP2 has since been attributed vital tumor-suppressive roles in regulating p53's pro-apoptotic responses (Samuels-Lev et al., 2001; Vousden and Prives, 2009), the regulatory role of p53-53BP1 interactions has remained ill defined.

In contrast, 53BP1 has been extensively characterized in the context of its contribution to the non-homologous end-joining (NHEJ) DNA double-strand break (DSB) repair pathway (Chapman et al., 2012; Zimmermann and de Lange, 2014). 53BP1 is an oligomeric chromatin reader that binds a combination of methylated (H4 Lys20me1/2) and ubiquitinated histone (H2A ubi-Lys15) epitopes within DSB-associated chromatin to promote NHEJ (Fradet-Turcotte et al., 2013). Its key function during DSB repair is to inhibit the nucleolytic processing of DNA ends, which it accomplishes by interactions with its downstream effector proteins, including Rif1 and PTIP (Zimmermann and de Lange, 2014). 53BP1-dependent NHEJ is crucial in the immune system, where it is necessary for immunoglobulin class switch recombination (CSR) and T cell receptor rearrangements (Difilippantonio et al., 2008; Manis et al., 2004; Ward et al., 2004), but this same process is oncogenic in cancers driven by *BRCA1* mutation or loss (Bouwman et al., 2010; Bunting et al., 2010). In these circumstances, 53BP1 blocks the accurate homologous recombination repair of DSBs and supports unrestrained NHEJ activity resulting in the massive genome rearrangements that drive oncogenesis (Chapman et al., 2012).

53BP1 also possesses tumor suppressor functions, and germline *53BP1* mutations predispose mice to T cell lymphoma, in a manner exacerbated by p53 loss (Morales et al., 2006; Ward et al., 2005). Thymic lymphomas derived from *53BP1*$^{-/-}$ *p53*$^{-/-}$ mice fall into two distinct cytogenetic categories: those characterized by aneuploidy that largely resemble equivalent tumors from *p53*$^{-/-}$ mice (Liao et al., 1998) and those without aneuploidy that harbor clonal translocations thought to arise from DSB repair intermediates that accumulate as a result of abortive antigen receptor gene-rearrangements and progress into oncogenic translocations when p53-dependent apoptotic responses are ablated (Difilippantonio et al., 2008; Morales

This is an open access article under the CC BY license (http://creativecommons.org/licenses/by/4.0/).

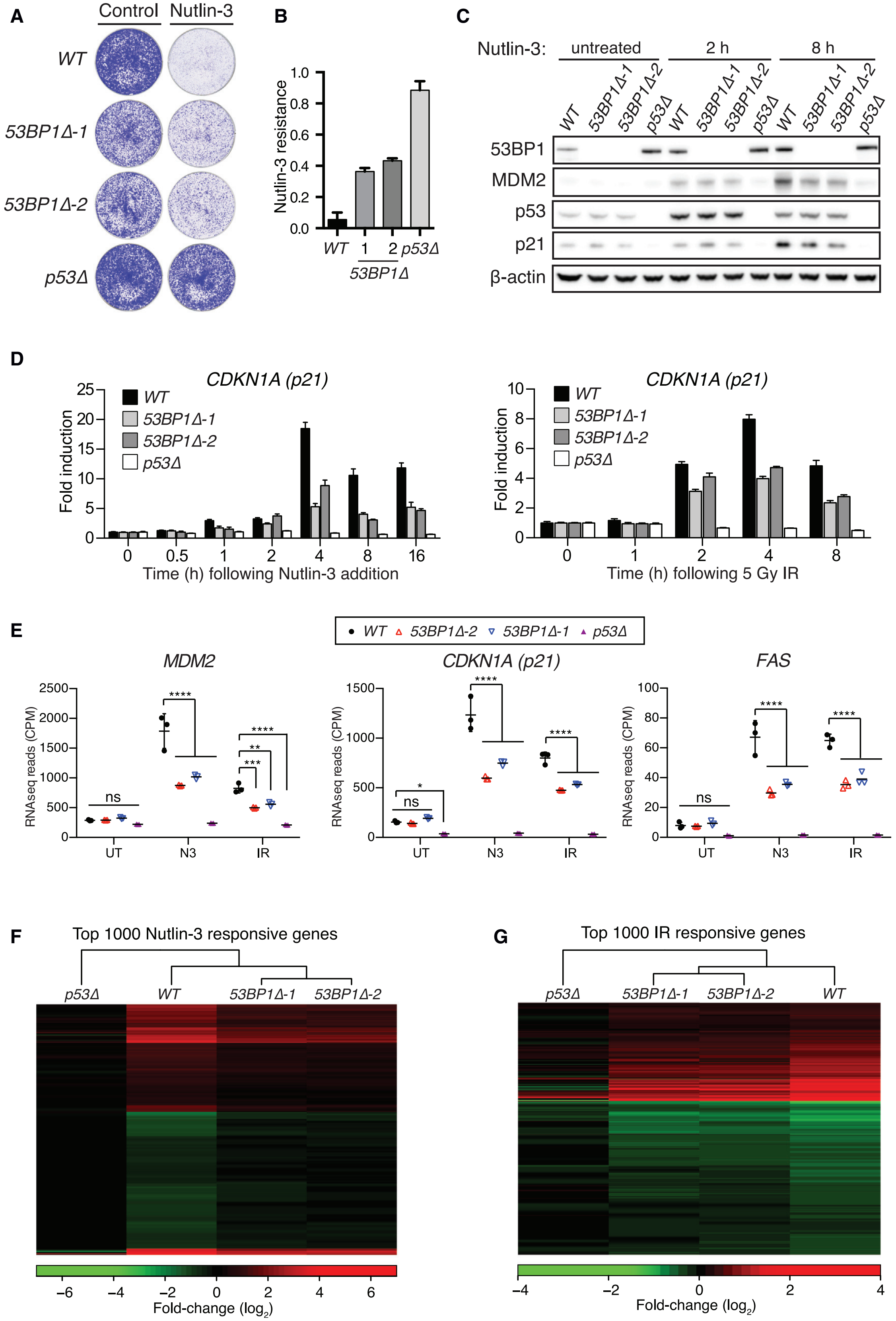

(legend on next page)

et al., 2006; Ward et al., 2005). While these results indicate a synergy between 53BP1 and p53 function, several lines of evidence have suggested putative roles for 53BP1 and p53 cooperation in tumor suppression (Huang et al., 2007; Iwabuchi et al., 1994; Iwabuchi et al., 1998). Interestingly, 53BP1 was identified in a genetic screen designed to reveal components of the p53 network that mediate cytotoxicity in response to Nutlin-3 (N3) (Brummelkamp et al., 2006). N3 is a small molecule antagonist of p53's inhibitor protein MDM2 that, by binding to MDM2, outcompetes its interaction with p53 resulting in p53 stabilization and activation (Vassilev et al., 2004). 53BP1 depletion conferred resistance to N3-induced senescence in MCF-7, a human breast cancer cell line typically used to model wild-type (WT) p53 function, providing strong evidence for a physiological 53BP1-p53 cooperation. Despite this, the functional relevance and molecular basis of such cooperation, and its relationship to 53BP1's DNA repair roles, has remained enigmatic.

Here, we use the synthetic viability phenotype of 53BP1-deficient cells in the presence of N3 as a means to dissect the molecular basis of 53BP1's p53-regulatory roles in the absence of crosstalk from its repair functions. We reveal a role for 53BP1 in directly modulating p53's transcriptional activities in response to multiple stimuli and find this is genetically, biochemically, and functionally separable from its DSB repair functions. Thus, 53BP1 integrates both p53-dependent functions and DNA repair activities to promote tumor suppression.

RESULTS

53BP1 Is Required for Optimal p53-Dependent Transactivation Events

To investigate 53BP1's contribution to p53-dependent signaling events, multiple isogenic *TP53BP1*-knockout lines were generated using CRISPR-Cas9 technology in MCF-7 (henceforth termed *53BP1Δ*; Figure S1A). In parallel, *TP53*-knockout lines were generated (*p53Δ*; Figure S1B) to control for p53 loss of function. As expected (Brummelkamp et al., 2006), *53BP1Δ* cells show reduced N3-induced growth arrest (Figure 1A). This reduced sensitivity to N3 was partial when compared to *p53Δ* lines (~40%; Figure 1B). Consistent with a disruption of p53 function in *53BP1Δ* cells, the N3-induced expression of the p53 targets p21 and MDM2 was strongly perturbed in 53BP1-deficient lines (Figure 1C). The fact that equivalent defects in MDM2 and p21 induction were also apparent following IR treatment (Figure S1C) suggested that 53BP1-mediated p53 regulation was not N3 specific, and 53BP1 likely modulates broader p53 function.

Next, 53BP1's contribution to p53-dependent transcriptional outputs was examined by quantitative RT-PCR. The induced expression of *p21* (*CDKN1A*) transcripts was consistently reduced by ~2-fold in *53BP1Δ* cells across all time points examined following each treatment (Figure 1D). Likewise, 53BP1 deficiency resulted in induction defects in other p53-responsive genes, including auto-regulatory (*MDM2*), cell-cycle (*GADD45A*, *TP53I3*), and pro-apoptotic (*BAX*, *PUMA*/*BBC3*) targets (Figure S1D). To characterize 53BP1's contribution to p53-dependent transcriptional programs genome wide, total RNA from WT, *p53Δ*, and two different *53BP1Δ* cell lines was analyzed by RNA-sequencing (RNA-seq) following mock, N3, and IR treatments. N3 and IR treatments induced significant alterations to the expression of 6,877 and 2,386 genes, respectively, in WT MCF-7, encompassing both gene activation and repression events (summarized in Table S1). These changes were almost exclusively p53 dependent, as in *p53Δ* cells N3 or IR induced significant changes to the abundance of only 13 and 25 transcripts, respectively. RNA-seq confirmed that N3- and IR-induced transactivation of p53-responsive genes was strongly attenuated in *53BP1Δ* cell lines (Figure 1E). *53BP1Δ* cell lines also displayed defects in p53-dependent gene repression events (Figure S2A). Moreover, the p53-dependent transcriptional programs induced by both treatments were dramatically impaired in 53BP1-deficient cells, with defects in gene activation and repression evident across p53-responsive genes (Figures 1F, 1G, S2B, and S2C). These defects are not due to differences in global transcription, as the abundance of non-treatment-responsive transcripts (Figure S2D) and candidate reference transcripts (Figure S2E) was equivalent across the different genotypes, irrespective of treatment. Taken together, these results indicate a key role for 53BP1 in propagating p53-dependent transcriptional programs. That this effect is seen genome wide excludes a role for 53BP1 in modulating p53 target specificity but rather implies that 53BP1 is a global enhancer of p53-dependent signals.

Structural Requirements for 53BP1-Dependent p53 Regulation

53BP1 is a protein scaffold and chromatin reader whose modular domain architecture supports binding to an array of interaction partners including post-translationally modified histones (Fradet-Turcotte et al., 2013; Zimmermann and de Lange, 2014).

Figure 1. 53BP1 Enhances p53-Dependent Transcriptional Programs

(A) Cells of indicated genotype were treated with 4 μM N3 for 7 days or mock treated, fixed, and stained with crystal violet.

(B) Quantification of two experiments as in (A), each plated in triplicate (mean ± SD).

(C) Immunoblot analysis of MCF-7 lines of indicated genotype following exposure to N3.

(D) *p21* transcript abundance in RNA isolates from cells treated with 4 μM N3 or 5 Gy IR, as evaluated by qRT-PCR. Fold induction calculated upon normalization against *HPRT1* transcripts. Data are representative of two independent experiments; mean ± SD.

(E) Representative p53-responsive transcripts from three RNA-seq replicates. Total RNA was sequenced from indicated MCF-7 lines following N3 (4 μM, 8 hr), IR (5 Gy, 4 hr), or control treatments. CPM, counts per million; *ns*, non-significant; *p < 0.05; **p < 0.01; ***p < 0.001; ****p < 0.0001 (two-way ANOVA). Bars represent mean ± SD.

(F and G) Heatmaps depicting $\log_2$ fold changes for top 1,000 responsive genes for each treatment relative to untreated controls (RNA-seq analysis of three biological replicates per condition). Ribo-depleted RNA was sequenced from indicated MCF-7 lines following N3 (4 μM 8 hr), IR (5 Gy, 4 hr), or control treatments. See also Figures S1 and S2 and Table S1.

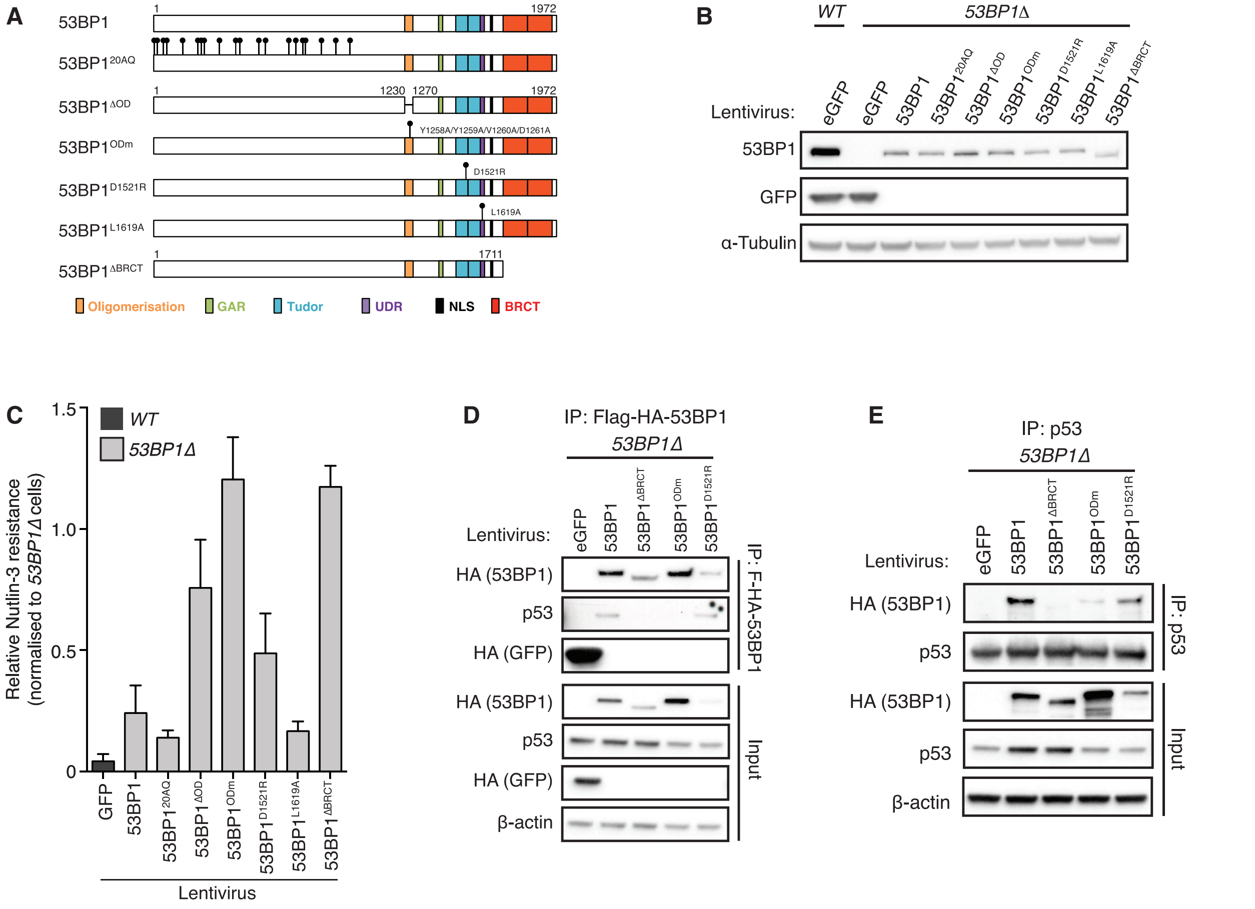

Figure 2. 53BP1-Dependent p53 Regulation Requires Oligomerization and BRCT Domain-Mediated p53 Interactions

(A) Schematic representation of the 53BP1 domain and point mutants examined for restoration of N3 sensitivity.

(B) Western blot showing comparable expression of 53BP1 mutants upon stable lentivirus-mediated transduction in *53BP1Δ* MCF-7.

(C) Indicated cell lines treated with N3 (4 μM) for 11 days or left untreated for 7 days were fixed and stained with crystal violet. Relative N3 resistance normalized to control (GFP)-complemented *53BP1Δ*. Mean of three biological replicates ± SD.

(D) Flag-HA-53BP1 proteins purified from cell lysates of indicated stably complemented *53BP1Δ* MCF-7 lines following N3 treatment. Interacting proteins analyzed by immunoblotting.

(E) Similar to (D), but the composition of p53 immunoprecipitates was analyzed. See also Figure S3.

The individual contributions of 53BP1's domains, motifs, and interaction partners toward its DNA repair roles have been well characterized yet remain uncharacterized in the context of p53 modulation. To address this, we generated *53BP1Δ* lines in which equivalent levels of WT and mutant 53BP1 proteins, or control protein (GFP), are stably expressed (Figures 2A and 2B) and assessed their ability to rescue N3 sensitivity. WT 53BP1 expression largely restored N3 sensitivity, when compared to control GFP-expressing *53BP1Δ* cells (Figure 2C). Likewise, expression of the 53BP1^{L1619A} ubiquitin-dependent recruitment (UDR) motif mutant, whose recruitment to DSB sites is compromised (Fradet-Turcotte et al., 2013), or the 53BP1^{20AQ} mutant that confers NHEJ defects owing to an inability to sustain Rif1 and PTIP recruitment at DSB sites (Callen et al., 2013; Chapman et al., 2013; Di Virgilio et al., 2013) both restored cellular N3 sensitivity to equivalent WT levels. In contrast, 53BP1 mutants bearing a deleted or mutated oligomerization domain (53BP1$^{\Delta OD}$ or 53BP1^{ODm}) or a C-terminal tandem-BRCT domain deletion (53BP1$^{\Delta BRCT}$) failed to restore N3 sensitivity, highlighting the important contribution of these two domains in N3 responses. Interestingly, an intermediate N3 sensitivity was detected in *53BP1Δ* cells complemented with the 53BP1^{D1521R} tudor domain mutant, indicating methyl-lysine-directed interactions with p53 (Huang et al., 2007; Kachirskaia et al., 2008), retinoblastoma protein pRb (Carr et al., 2014), and/or histone H4 methylated on lysine 20 (H4K20me1/2) (Botuyan et al., 2006) might additionally influence p53-dependent transcription. Oligomerization, tudor, and BRCT domain mutant 53BP1 proteins also showed their expected localization patterns, indicating that their inability to restore WT N3 sensitivity is not due to aberrant protein expression or localization (Figure S3).

Given the importance of the 53BP1 oligomerization, BRCT, and tudor domains in conferring N3 sensitivity, the function of each domain in 53BP1-p53 protein interactions was examined

in co-immunoprecipitation experiments. Flag-HA-tagged WT or tudor mutant (53BP1^{D1521R}) 53BP1 proteins both co-precipitated p53 from cell lysates (Figure 2D). Hence, reported tudor domain-mediated binding to p53 methyl-lysine residues (Huang et al., 2007; Kachirskaia et al., 2008) does not mediate bulk cellular p53-53BP1 interactions. In contrast, p53 was undetectable in 53BP1$^{\Delta BRCT}$ immunoprecipitates or when multiple conserved residues within the 53BP1 oligomerization domain were mutated (Zgheib et al., 2009) (Figure 2D). Concordant p53-53BP1 interaction profiles were reproduced upon immunoprecipitation of endogenous p53 from each mutant 53BP1 cell lysate (Figure 2E). These data indicate that the 53BP1 tandem-BRCT domain is responsible for interacting with p53, and this interaction is significantly destabilized by mutation of the 53BP1 oligomerization domain. Combined with our N3 sensitivity data (Figure 2C), this shows that p53-53BP1 interactions are essential for normal p53 function.

53BP1-Dependent p53 Regulation and DSB Repair Activities Are Distinct and Separable

Mutant *53BP1* alleles that encode C-terminal BRCT domain deletion mutations are indistinguishable from WT in their ability to support physiological NHEJ during CSR and pathological NHEJ events in the context of *BRCA1* deficiency and dysfunctional telomeres (Bothmer et al., 2011; Lottersberger et al., 2013). However, several recent reports have suggested roles for the 53BP1 BRCT domain in heterochromatin DSB repair (Baldock et al., 2015; Lee et al., 2010; Noon et al., 2010). Given the importance of this domain in mediating functional p53 interactions, we next explored the relative contributions of this domain toward p53 regulation and DSB repair. Paired guide RNA molecules were used to direct the Cas9-dependent excision of genomic sequences encoding 53BP1's BRCT domain (Figure 3A), and two validated isogenic clones were further characterized (denoted *53BP1*$^{\Delta BRCT}$, clones A and B). Consistent with previous work in which the 53BP1-BRCTs were deleted in the mouse germline (Bothmer et al., 2011), 53BP1 expression in *53BP1*$^{\Delta BRCT}$ cell lines was reduced relative to WT (Figure 3A). Nevertheless, *53BP1*$^{\Delta BRCT}$ cells proliferated at a normal rate in comparison to slow-growing *53BP1Δ* cells (Figure 3B and data not shown), suggesting that reduced 53BP1$^{\Delta BRCT}$ protein levels can support normal cell growth. Despite this, *53BP1*$^{\Delta BRCT}$ lines were N3 resistant like *53BP1Δ* cells (Figure 3B) and showed attenuated p53-dependent induction of MDM2 and p21 following N3 and IR treatments (Figures S4A and S4B), confirming the importance of an intact BRCT domain in modulating p53 function. As the BRCT domains are largely dispensable for 53BP1-dependent NHEJ (Bothmer et al., 2011; Lottersberger et al., 2013), we reasoned the normal growth rate of *53BP1*$^{\Delta BRCT}$ lines indicated that 53BP1$^{\Delta BRCT}$ expression could support 53BP1-dependent repair activities. In line with this, 53BP1$^{\Delta BRCT}$ protein was recruited into IRIF (Figure 3C), where it supported normal Rif1 recruitment (Figure S4C), events essential for its canonical NHEJ functions (Chapman et al., 2013; Di Virgilio et al., 2013; Escribano-Díaz et al., 2013; Zimmermann et al., 2013). To investigate this potential separation of function further, the relative IR sensitivity of WT, *53BP1Δ*, *p53Δ*, and *53BP1*$^{\Delta BRCT}$ cell lines was compared (Figure 3D). As expected (Chapman et al., 2013; Iwabuchi et al., 2006), asynchronous *53BP1Δ* cultures were moderately IR sensitive (Figure 3D). Remarkably, however, *53BP1*$^{\Delta BRCT}$ lines showed significantly improved survival following IR treatments when compared to WT cells, closely mimicking the radioresistance evident in *p53Δ* cells (Figure 3D). It is known that p53 deficiency bypasses apoptotic and cellular senescence responses following IR treatments (Krenning et al., 2014; Lowe et al., 1993). Our data thus suggest that 53BP1 BRCT-mediated p53 interactions positively influence these functions. We therefore speculated that the expression of 53BP1 mutants that only disrupt its repair activities might further increase the sensitivity of *53BP1Δ* cells to IR treatments by restoring their ability to promote p53-dependent cell-cycle arrest or exit responses. In line with this, stable expression of the 53BP1 UDR mutant 53BP1^{L1619A}, which is unable to assemble at DSB sites (Fradet-Turcotte et al., 2013) yet is proficient for N3 responses, increased the radiosensitivity of *53BP1Δ* cells, in contrast to WT 53BP1 expression that suppressed IR sensitivity (Figure 3E). These data therefore reveal a major function for the 53BP1 BRCT domain in regulating p53-dependent cell-cycle exit responses and argue that these activities function independently of 53BP1-dependent DNA repair.

The 53BP1 BRCT Domain Mediates Bivalent Interactions with p53 and USP28

Tandem-BRCT domains are common structural features of DDR proteins that typically possess a phosphopeptide-binding surface within the inter-BRCT repeat interface that interacts with phospho-serine-containing motifs in partner proteins (Reinhardt and Yaffe, 2013). Somewhat atypically, the 53BP1 tandem-BRCT-mediated p53 interaction utilizes the opposite face of the BRCTs, which contains conserved surface residues spanning the first BRCT (BRCT1) and the inter-BRCT linker that mediate multiple contacts with residues in the L3-loop of the p53 DBD (Figure 4A) (Joo et al., 2002). In the p53-53BP1 co-crystal structure, the conserved phospho-binding pocket within the 53BP1 tandem-BRCT domain remains available, suggesting additional BRCT-mediated protein interactions could contribute to p53 modulation. We thus investigated the contributions of the p53- and phospho-binding surfaces of the 53BP1 BRCTs in p53 responses. In validation of structural models (Derbyshire et al., 2002; Joo et al., 2002), alanine substitution of p53-binding residues Asn1845 and Asp1861 in 53BP1 (Figure 4A) weakened 53BP1-p53 interactions (Figure 4B). Moreover, p53-binding was more dramatically destabilized by bulky arginine substitutions at these positions (Figure 4B). Notably, the differential effects of Ala and Arg mutations at these positions on p53 binding were consistent with the greater N3 resistance of 53BP1^{N1845R}- and 53BP1^{D1861R}-expressing *53BP1Δ* lines, compared to their 53BP1^{N1845A}- and 53BP1^{D1861A}-expressing counterparts (Figures 4C and S5), confirming the importance of these residues in regulating p53 function. In contrast, p53-53BP1 interactions were enhanced by 53BP1^{R1811A} and 53BP1^{K1814M} phospho-binding pocket mutations (Figures 4A and 4B), suggesting the 53BP1 BRCTs might bind p53 and phospho-ligands within distinct and competing protein complexes. Intriguingly, 53BP1^{R1811A} expression could only partially suppress N3 resistance in *53BP1Δ* cells (Figure 4C), which was in contrast to 53BP1^{K1814M} expression

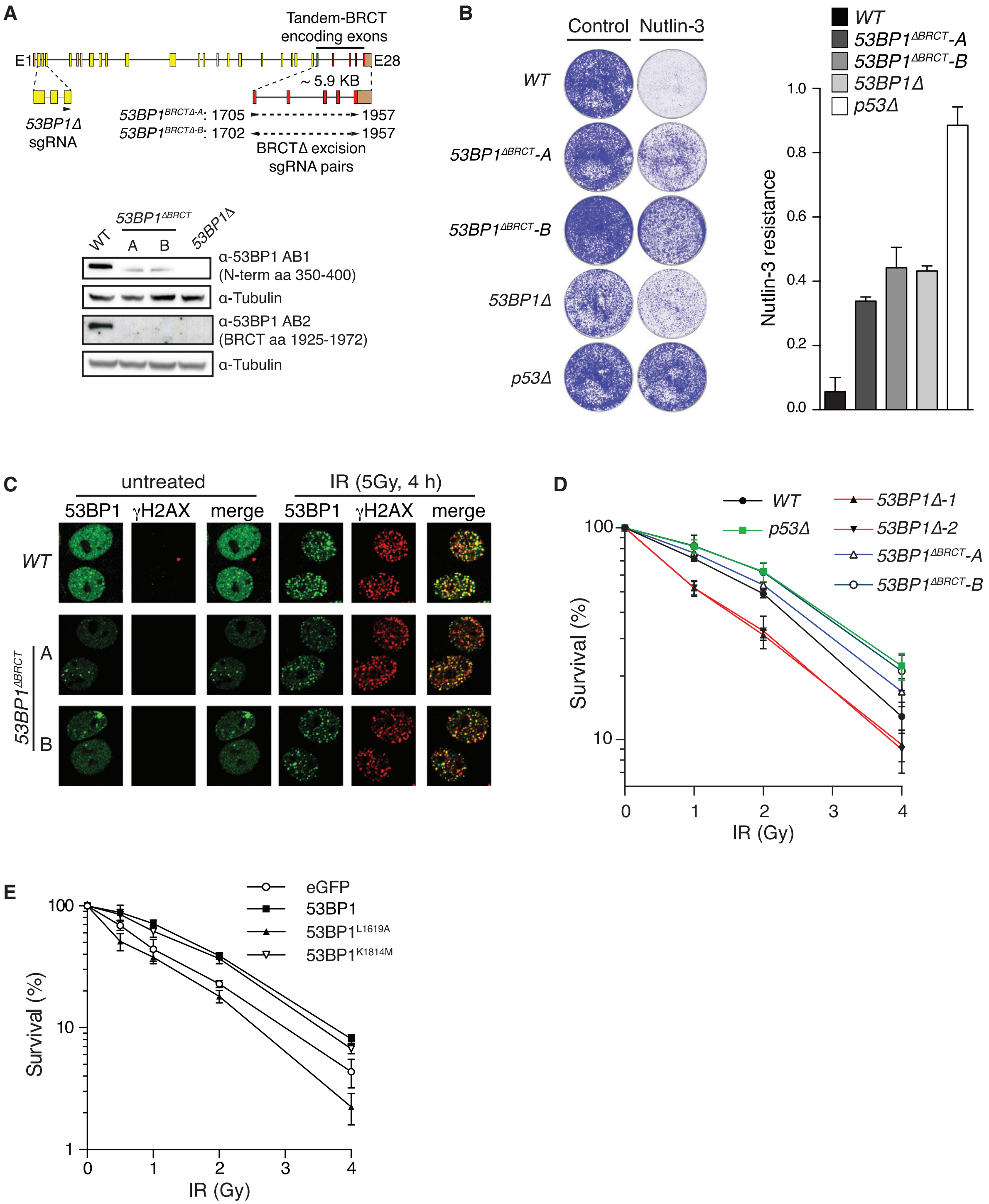

Figure 3. 53BP1's p53-Regulatory and DSB Repair Activities Are Distinct and Separable

(A) Generation of *53BP1*$^{\Delta BRCT}$ alleles using CRISPR-Cas9 technology. Top: schematic representation of *TP53BP1* gene locus showing the two sgRNA pairs (triangles) used to excise BRCT-encoding exonic sequences. Bottom: immunoblot of lysates prepared from two *53BP1*$^{\Delta BRCT}$ MCF-7 lines with epitope-specific 53BP1 antibodies showing the expression of mutant 53BP1$^{\Delta BRCT}$ protein.

(B) N3 resistance assay was performed as in Figures 1A and 1B. Mean ± SD (n = 2, plated in triplicate).

(C) Subnuclear 53BP1 localization was analyzed by indirect immunofluorescence in indicated cell lines following mock or irradiation (5 Gy, 4 hr) treatments.

(legend continued on next page)

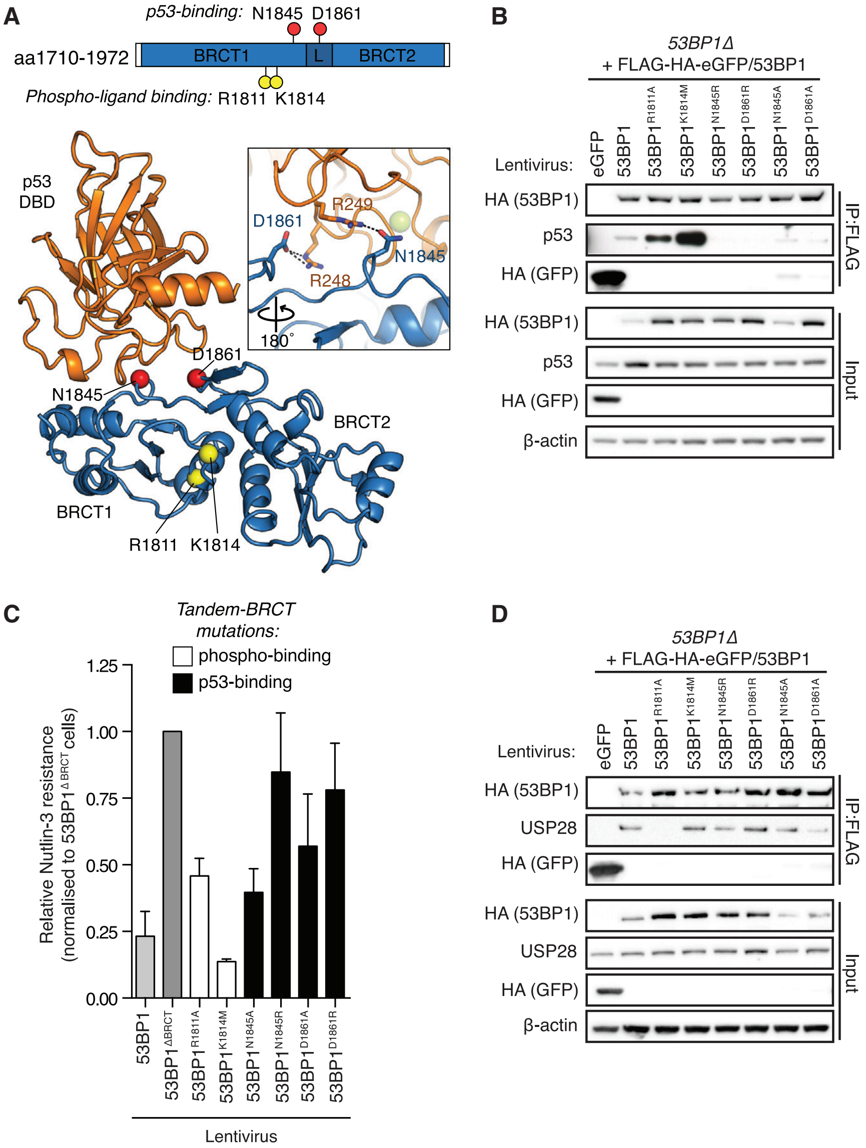

Figure 4. The 53BP1 BRCT Domain Mediates Bivalent Interactions with p53 and USP28

(A) The p53-53BP1 co-crystal structure (PDB: 1KZY; Joo et al., 2002) indicates the distinct tandem-BRCT surface residues involved in p53- and phospho-ligand interactions (red and yellow spheres, respectively). Dotted lines in zoom panel indicate hydrogen bonds between residues in 53BP1 and p53.

(B) The interaction of p53 with indicated FLAG-HA-53BP1 protein complexes was probed by immunoblotting, following immunopurification from N3-treated cell lysates.

(C) N3 resistance of *53BP1Δ* cells complemented with indicated 53BP1 point mutants. N3 resistance was normalized to 53BP1$^{\Delta BRCT}$-complemented lines. Mean ± SD (n = 3, in triplicate).

(D) As in (B), but FLAG-HA-53BP1 protein complexes were examined for USP28 co-purification. See also Figure S5.

that rescued N3 sensitivity to levels equivalent to WT-53BP1 complementation. This suggested that Arg1811 might support interactions with an additional 53BP1 interactor independently of Lys1814, despite their usual cooperation in phospho-peptide binding (Baldock et al., 2015; Kleiner et al., 2015). The Ubiquitin Specific Protease USP28 is a reported interactor of the 53BP1 BRCT domain (Knobel et al., 2014; Zhang et al., 2006), and a USP28-targeting shRNA construct was modestly enriched in the same loss-of-function screen that identified 53BP1 as a suppressor of N3-induced senescence (Brummelkamp et al., 2006). We found that 53BP1 binding to USP28 was specifically attenuated by the R1811A BRCT mutation, yet unaffected by the K1814M phospho-binding or p53-binding mutations (Figure 4D). These data show that 53BP1 binds independently to p53 and USP28 via distinct BRCT domain surfaces and pointed toward a potential cooperative role for USP28-53BP1 complexes in p53 regulation.

USP28 Is a Component of the p53-53BP1 Axis

USP28 was originally implicated in counteracting the proteasomal degradation of 53BP1, CHK2, and multiple additional DDR proteins (Zhang et al., 2006). However 53BP1's stability or repair activities have since been found to be unaffected by germline *USP28* deletion in mice (Knobel et al., 2014). We thus considered if USP28 might cooperate with 53BP1 in regulating p53. Multiple isogenic *USP28Δ* MCF-7 lines generated using CRISPR-Cas9 (Figure S6A) displayed N3 resistance at levels equivalent to *53BP1Δ* cells (Figures 5A and 5B). Moreover, the N3 resistance of a *USP28Δ 53BP1Δ* double-knockout line was not enhanced over single mutants (Figure 5C), confirming USP28 and 53BP1 function epistatically in this context. Further consistent with USP28's participation in p53-dependent signaling, *USP28Δ* cells were defective at inducing MDM2 and p21 protein expression following N3 treatment (Figure 5D), correlating with an impaired stimulation of p53-dependent transcription under similar conditions (Figure 5E). Lastly, USP28 deubiquitinase (DUB) activity was essential in this context, as

(D) The survival of MCF-7 cell lines of indicated genotype following control or X-ray irradiation treatments was assessed by colony survival assay. Mean ± SD (n = 3, plated in triplicate).

(E) The survival of *53BP1Δ* cells stably complemented with indicated 53BP1 transgenes following control or X-ray irradiation treatments was assessed as in (D). Mean ± SD (n = 3, plated in triplicate). See also Figure S4.

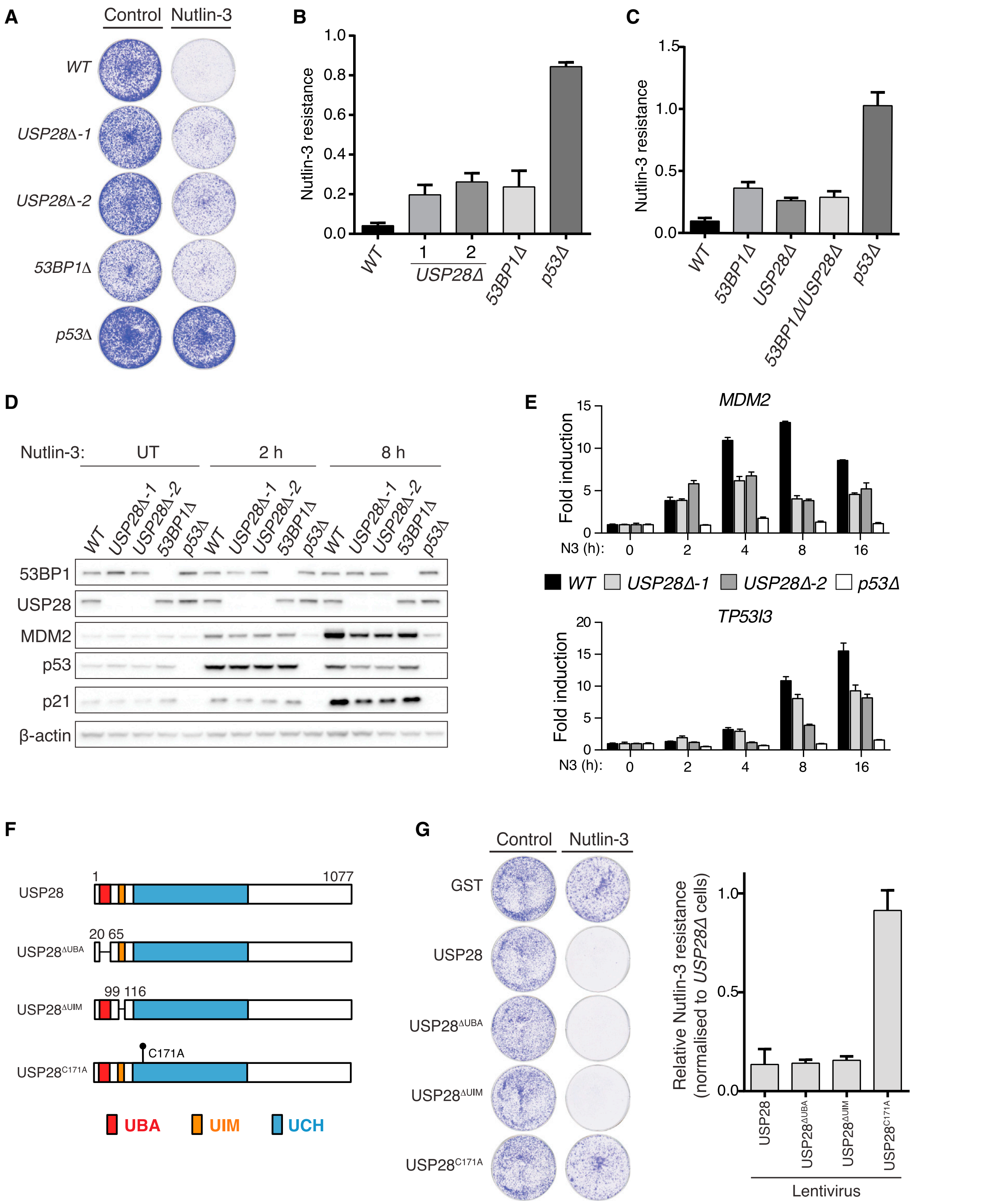

Figure 5. USP28 Is a Component of the p53-53BP1 Axis

(A) N3 resistance of indicated cell lines assessed as in Figures 1A and 1B.

(B) Quantification of (A). Mean ± SD (n = 3).

(C) N3 resistance of a *53BP1Δ*, *USP28Δ* double-knockout cell line relative to single mutants. Mean ± SD (n = 3, in triplicate).

(D) Lysates prepared from indicated control and N3-treated cell lines were immunoblotted with indicated antibodies.

(E) The transactivation of p53-responsive genes *MDM2* and *TP53I3* was assessed by qRT-PCR in indicated cell lines. Indicated fold changes were calculated upon normalization against *HPRT1* transcripts. Data are representative of two independent experiments; mean ± SD.

(legend continued on next page)

restored expression of WT USP28, or mutants in which its N-terminal ubiquitin-binding UBA or UIM domains were deleted, restored N3 sensitivity, while the catalytic dead USP28^{C171A} mutant (Zhang et al., 2006) could not (Figures 5F and 5G). Consistent with this, the expression of WT or ubiquitin-binding mutant USP28 rescued p21 and MDM2 induction defects in *USP28Δ* cells, whereas expression of USP28^{C171A} did not (Figure S6B). Together, these data reveal a co-regulatory role for 53BP1 and USP28 in supporting p53 function.

53BP1 and USP28 Are Co-regulators of p53-Dependent Cell-Cycle Checkpoints

Central to p53's suppression of tumor growth is its ability to sustain cell-cycle arrest and exit responses following stress signals (Vousden and Prives, 2009). The cooperation between 53BP1 and USP28 in triggering p53-dependent growth arrest following N3 treatments prompted us to investigate their function in arresting the cell cycle in response to DNA damage. p53-dependent p21 upregulation is essential for G1-phase arrest in response to irradiation (Dulić et al., 1994), and 53BP1 has additionally been implicated in enforcing this checkpoint in a manner involving interactions with the TopBP1 checkpoint protein (Cescutti et al., 2010). To examine the relative contributions of 53BP1, its BRCT domains, and USP28 to G1 checkpoint activation, the progression of serum-released WT, *53BP1Δ*, *53BP1^{ΔBRCT}*, and *USP28Δ* cultures into S phase was monitored following mock or IR treatments (Figure 6A). While untreated WT cultures readily proceeded from G1 into S following serum release, irradiated cells remained arrested in G1 (Figure 6B), consistent with robust activation of the G1 checkpoint. As expected (Dulić et al., 1994), G1 arrest was completely p53 dependent; IR-treated *p53Δ* cells re-entered the cell cycle with kinetics indistinguishable from non-irradiated controls (Figure 6B). The G1 checkpoint activation was strongly influenced by 53BP1 and its BRCT domains, as a significant proportion of *53BP1Δ* and *53BP1^{ΔBRCT}* cell lines entered S phase at time points where WT cells remained robustly arrested, with escape from G1 arrest increasing to around half of all cells at 22 hr following irradiation (Figures 6C and 6D). These defects correlate well with the intermediate effects that were seen for 53BP1-deficient and 53BP1^{ΔBRCT} mutant cell lines in N3 and IR responses, indicating that while 53BP1 and its BRCT-mediated p53 interactions function to reinforce p53 function, some residual p53 function remains in the absence of this regulation. Similar intermediate G1 checkpoint defects were observed in *USP28Δ* cells (Figures 6E and 6F), revealing a role for USP28 in regulating p53-dependent checkpoint responses. Thus, our data identify an important role for 53BP1-bridged p53-USP28 interaction in regulating p53 function in human cells.

53BP1 and USP28 Co-stimulate p53-Responsive Element Interactions

We next considered the mechanism by which 53BP1-USP28 complexes might modulate p53-dependent transcription. p53 transcriptional activities under basal and stimulated conditions are fine-tuned via regulated changes to p53's stability, subcellular localization, and DNA-binding activities (Kruse and Gu, 2009; Vousden and Prives, 2009). However, we were unable to link the defects we detected to alterations in the stability (Figures S7A and S7B) or sub-cellular localization (Figure S7C) of p53 in untreated or irradiated *53BP1Δ* or *USP28Δ* cells. To determine the contribution of 53BP1-USP28 to p53-responsive element (p53-RE) binding, we performed chromatin immunoprecipitation (ChIP) experiments under basal and N3-stimulated conditions. N3 stimulated ~7-fold increases in p53 binding to its two binding sites in the *p21* promoter in WT cells (Figures 7A and 7B). Both of these binding events strongly relied on 53BP1 and USP28 status. Specifically, basal p53 binding to both p53 REs was reduced by ~2-fold in both *53BP1Δ* and *USP28Δ* cells, and its induction upon N3 treatment was severely impaired, resulting in overall ~3-fold reductions in p53 residency at both loci upon stimulation (Figure 7B). These defects corresponded to a diminished induction of histone acetylation events associated with p53-dependent gene transactivation (Donner et al., 2007). Specifically, histone H4 pan-acetylation (H4ac) across the *p21* promoter was reduced by ~3-fold in N3-treated *53BP1Δ* and *USP28Δ* cultures relative to WT (Figure 7C), with equivalent defects detected in H3 K9 acetylation (H3 K9ac) across promoter and 5′ intragenic regions (Figure 7D). These abnormalities correlated to diminished elongating RNA Pol2 residency across the *p21* gene body in *53BP1Δ* and *USP28Δ* cells (Figure 7E), a defect entirely consistent in magnitude with the p53-dependent transcriptional defects detected in earlier experiments (Figures 1F, 1G, and 5E). These abnormalities were not unique to the *p21* locus, as p53 DNA binding, histone acetylation, and RNA Pol2 residency defects were reproduced at p53 REs within multiple additional p53-responsive genes in *53BP1Δ* and *USP28Δ* cells (Figure 7F). In line with our transcriptomic analyses (Figure 1), our data collectively reveal a function for 53BP1-dependent bivalent interactions with USP28 and p53 in enhancing p53-promoter element interactions, thereby amplifying p53-dependent transcriptional programs.

DISCUSSION

The repair activities of 53BP1 synergize with p53 in tumor suppression (Difilippantonio et al., 2008; Morales et al., 2006; Ward et al., 2005), yet interactions between 53BP1 and p53 have hinted at additional cooperative contributions. Taking advantage of the discovery that 53BP1 and p53 co-participate in N3-induced senescence responses (Brummelkamp et al., 2006), we now reveal the molecular basis of 53BP1-p53 cooperation. In contrast to initial speculation that p53-53BP1 cooperation relied on a synergy between p53-dependent transcriptional responses and the signaling of stochastic DNA damage via 53BP1 (Brummelkamp et al., 2006), we find that 53BP1 plays a direct role in enhancing the magnitude of p53-dependent gene

(F) Schematic representation of the USP28 protein domain architecture and the domain and point mutants used in this study. UBA, ubiquitin-associated domain; UIM, ubiquitin-interaction motif; UCH, ubiquitin carboxyl-terminal hydrolase domain.

(G) Visual and quantitative analysis of N3 resistance of *USP28Δ* cell lines stably transduced with indicated USP28 expressing lentiviruses. N3 resistance relative to a control (GST)-transduced *USP28Δ* cell line was calculated as in (A) and (B). Mean ± SD (n = 3, in triplicate). See also Figure S6.

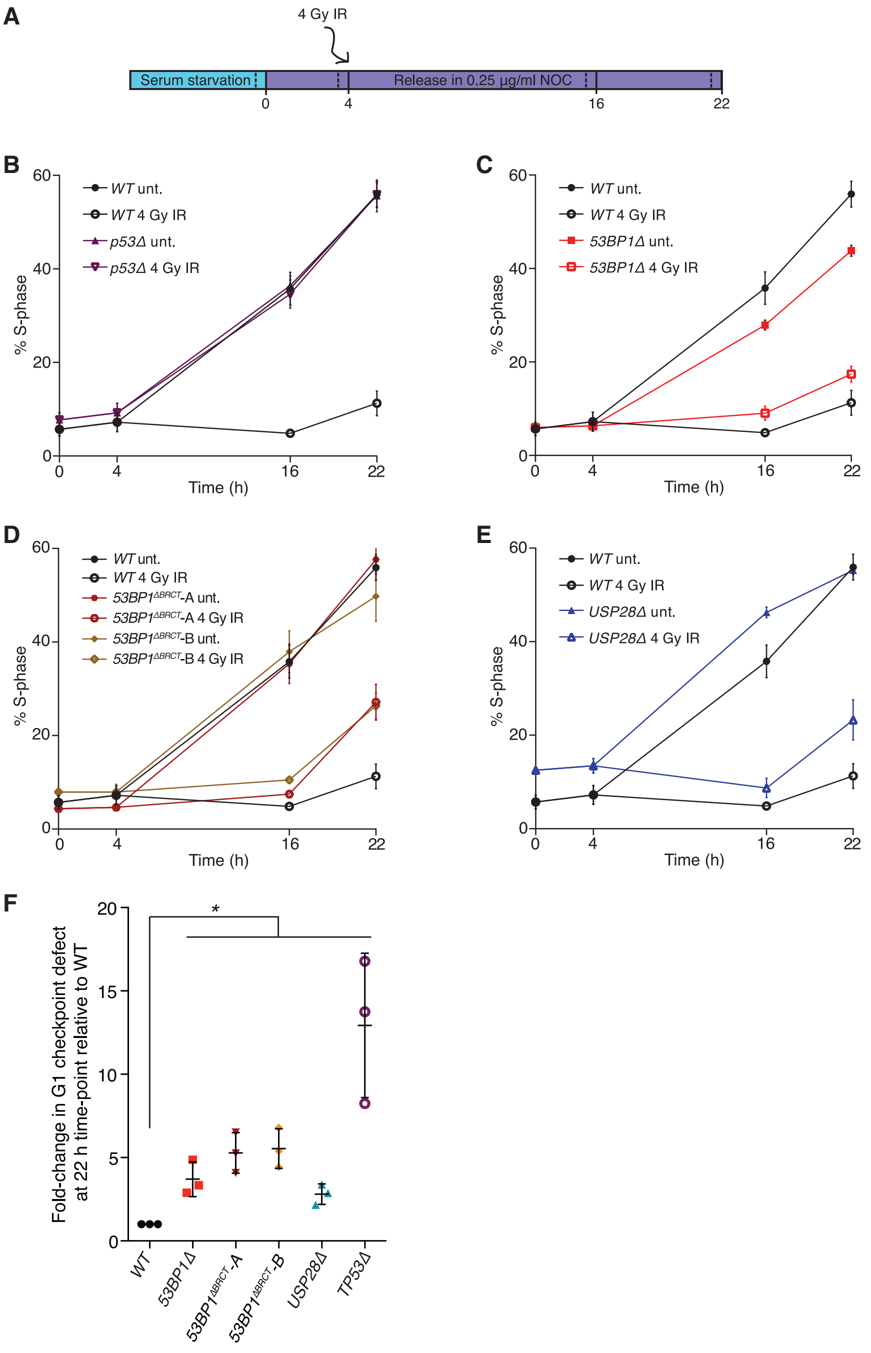

Figure 6. 53BP1 and USP28 Co-regulate p53-Dependent G1 Checkpoint Arrest

(A) Schematic representation of the G1 checkpoint assay. Briefly, cells serum arrested in G0 for 24 hr were released in serum-containing medium supplemented with 0.25 µg/ml nocodazole before irradiation (4 Gy) and collection at indicated time points for cell-cycle analysis. Solid bars indicate experimental time points. Dotted lines indicate time of BrdU pulse addition.

(B–E) S phase cell indices for each indicated condition as defined by BrdU pulse labeling immediately prior to collection at indicated time points. Relative cell-cycle phase distributions were calculated by flow cytometry in BrdU immunolabeled cells counterstained for DNA content. Three biological replicates mean ± SD.

(F) Significance of G1 checkpoint defects detected in (B)–(E) as a measurement of changes in S phase indices in irradiated samples between 4 and 22 hr time points. IR-treated sample values first corrected against the S phase index change in the corresponding untreated sample then normalized against the WT value for each experiment. Values are plotted as fold change relative to WT. Mean ± SD; *$p < 0.05$ (Student's t test).

activation and repression events triggered by N3 and IR. Perhaps surprisingly, 53BP1-dependent p53-regulatory and DNA-repair activities can be entirely separated at the level of mutations in 53BP1 that selectively either block p53 binding or prevent its enrichment at DSB sites and interaction with downstream repair effector proteins. Thus, our data demonstrate that 53BP1 DNA repair and p53-regulatory roles are independent of one another and rely on thier association with distinct interaction partners. This separation of function is best illustrated by mutation of 53BP1's tandem-BRCT domain, where our data are consistent with previous models in which the 53BP1 tandem-BRCT domain is dispensable for its canonical repair activities (Bothmer et al., 2011; Lottersberger et al., 2013; Ward et al., 2006). In addition, by disrupting p53-53BP1 interactions with single point mutations, we have validated previous structural models of the p53-53BP1 interaction (Derbyshire et al., 2002; Joo et al., 2002) and revealed that these interactions are critical for optimal p53 function.

Recent evidence implicates the 53BP1 tandem-BRCT domain in binding the Rad50, ATM, and γH2AX proteins in interactions important for DSB resolution within heterochromatin (Baldock et al., 2015; Lee et al., 2010; Noon et al., 2010). In these studies, loss of this function resulted in persistent γH2AX foci in irradiated 53BP1 BRCT mutant-expressing cell lines. However, our data suggest that this defect plays no role in the IR hypersensitivity of *53BP1Δ* lines, as reconstitution of a 53BP1 BRCT domain phospho-ligand binding mutant (53BP1^{K1814M}) that blocks γH2AX binding (Baldock et al., 2015; Kleiner et al., 2015) restored the IR resistance of *53BP1Δ* lines to WT levels (Figure 3E). In fact, the creation of *53BP1^{ΔBRCT}* alleles actually enhanced cellular survival following IR treatments relative to WT, correlating closely with the radioresistant phenotype we and others have described for cells of diminished p53 function (Figure 3D) (Lowe et al., 1993) while also reflecting an overall proficiency

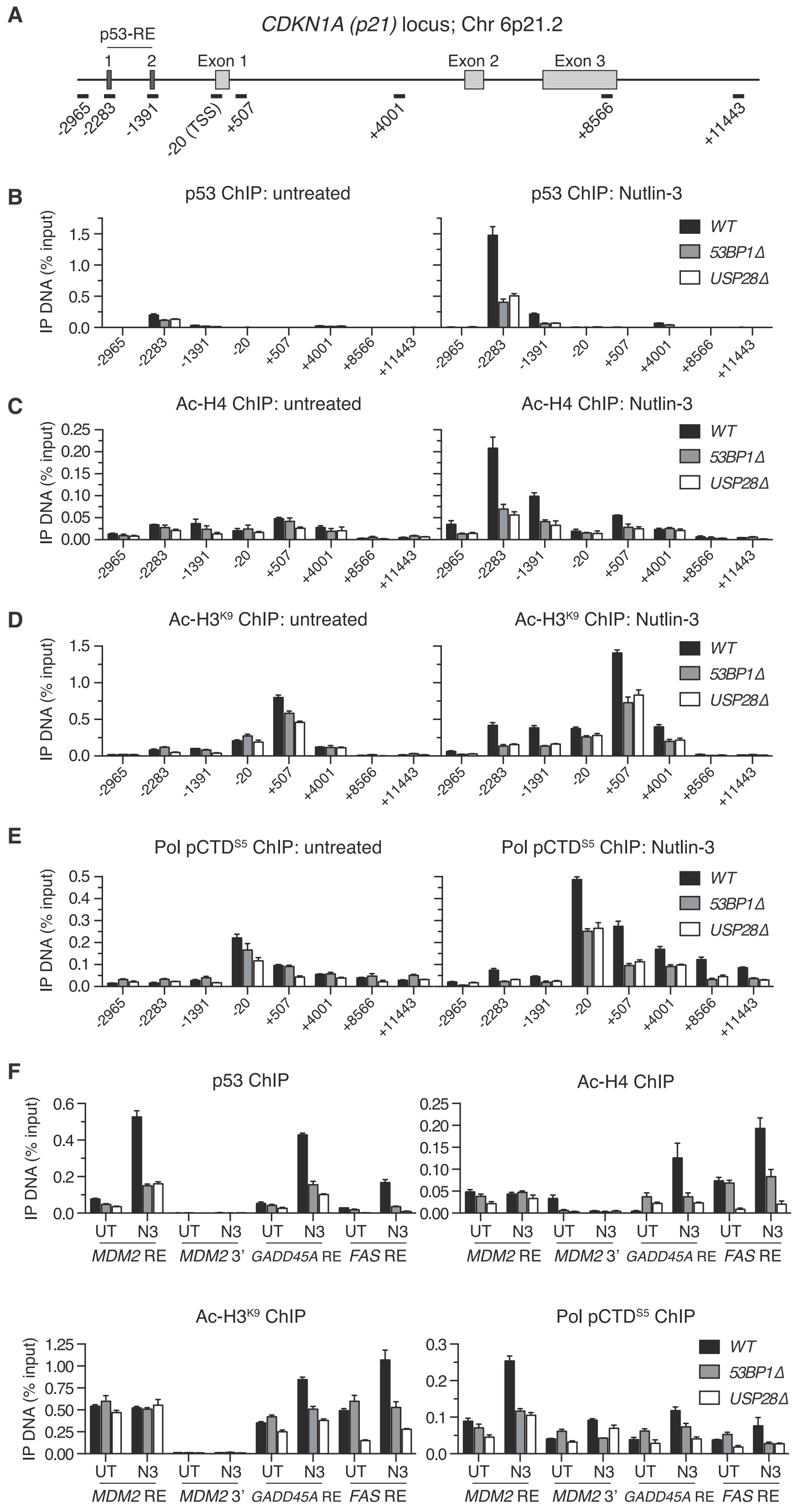

Figure 7. 53BP1 and USP28 Enhance p53-Responsive Element Interactions

(A) Schematic of the *p21* (*CDKN1A*) locus indicating high and low affinity p53 binding sites (p53-REs 1 and 2, respectively) and general gene structure. qPCR amplicons used to quantify ChIP-enriched DNA are indicated (bars) and named according to their relative distance to the transcription start site (TSS).

(B–E) ChIP was performed in chromatin extracts prepared from indicated untreated or N3-treated (4 μM, 6 hr) cell lines using antibodies against p53 (B), pan-acetyl-H4 (C), acetyl-histone H3 Lys9 (D), and RNAP2 CTD phosphorylation (pSer5) (E). Immunoprecipitated DNA was calculated as a percentage of total input DNA. Data are representative of two independent experiments with PCRs performed in triplicate. Mean ± SD.

(F) As in (B)–(E), but using p53-RE-spanning amplicons in indicated p53-responsive genes, except MDM2 3′, which indicates a control amplicon within the last *MDM2* coding exon. See also Figure S7.

for 53BP1 BRCT mutant protein in supporting bulk 53BP1-dependent DNA repair activities. Thus, we propose that the direct regulation of p53 represents a prime function for the conserved 53BP1 tandem-BRCT domain.

In addition to distinct 53BP1 domain requirements for p53 and DNA repair regulation, some 53BP1 domain features were found to contribute to both roles. 53BP1's recruitment to DSB sites is critical for its repair functions and involves auto-oligomerization and bivalent nucleosomal contacts (Fradet-Turcotte et al., 2013). Here, we report that 53BP1 oligomerization is similarly crucial for 53BP1-dependent p53 regulation. Mutations that prevent oligomerization also destabilize p53-53BP1 interactions (Figures 2D and 2E), suggesting 53BP1 oligomerization either reinforces BRCT-mediated p53 interactions or increases its avidity toward multimeric p53 complexes. Interestingly, 53BP1 UDR and tudor domain mutations elicit differential effects on p53 function, despite their cooperation during DSB repair. Specifically, 53BP1 UDR point mutants were able to fully rescue N3 sensitivity, while methyl-lysine-binding tudor domain mutants did so only partially. While the tudor domain's participation in this context might be explained by its ability to mediate binding to methyl-K370/K382 residues in p53 (Huang et al., 2007; Kachirskaia et al., 2008), we were unable to detect any significant impact of tudor domain mutation on bulk 53BP1-p53 interactions, in contrast to previous findings (Huang et al., 2007). Despite this, it is noteworthy that p53-K370me2-mediated 53BP1 interactions were speculated to fine tune p53 function by potentially enhancing p53-RE interactions in a manner counteracted by the lysine-demethylase LSD1 (Huang et al., 2007). Indeed, this is in line with our observations that 53BP1 protein complexes have a role in stimulating p53-RE interactions (see below).

Lastly, we establish a role for USP28-53BP1 cooperation in regulating p53-dependent transcription, attributing function to protein interactions first described a decade ago (Zhang et al., 2006). Our data provide a mechanistic explanation for USP28's reported contribution to apoptotic responses via a Chk2-p53-PUMA pathway, and in agreement we detected defects in the p53-dependent activation of PUMA and other genes in *USP28Δ* cell lines (Figures S6C and 5E). Importantly, the binding of p53 to p53-REs across multiple genes was consistently diminished under both basal and stimulated conditions in 53BP1- and USP28-deficient cell lines to similar levels, confirming the positive regulation of p53 DNA binding to be a central role for 53BP1-USP28 complexes. While we have not yet defined the precise mechanism in which 53BP1-USP28 complexes stimulate p53 DNA binding, our data show that the DUB activity of USP28 is required in this context. Given that p53 activity is quenched via MDM2-dependent p53 ubiquitination (Brooks and Gu, 2006; Toledo and Wahl, 2006), it is tempting to speculate that a 53BP1-dependent targeting of USP28 into p53-protein complexes might counteract such events. In this light, it is interesting that HAUSP (USP7), a DUB originally proposed to counteract MDM2-dependent p53 ubiquination (Li et al., 2002), has since been found to target MDM2 as its prime substrate, reconciling contradictory observations that p53 was stabilized in cells of diminished HAUSP (Cummins et al., 2004; Li et al., 2004). Thus, the existence of alternative DUBs that target p53 has been speculated (Brooks and Gu, 2006), and USP28 represents an attractive candidate. While undoubtedly important for p53 turnover, not all of MDM2's inhibitory roles on p53 activity can be explained at this level, and MDM2 has been proposed to inhibit p53 activities in other ways (Brooks and Gu, 2006). A ubiquitination-dependent inhibition of p53 DNA-binding activity could represent an additional means by which p53 activity can be down-tuned by MDM2. If so, the 53BP1-USP28-p53 axis identified here could represent a means by which such inhibition can be reversed, rapidly enhancing p53's transcriptional potential upon release from inhibitory MDM2 complexes. In this regard, it is noteworthy that while 53BP1 readily co-immunoprecipitated p53 from cell lysates, we were unable to detect 53BP1 at p53-REs by ChIP. We therefore hypothesize that 53BP1-USP28 complexes interact with nucleoplasmic p53 pools, where they function to prime p53 DNA-binding activity. Such a notion would be consistent with structural observations that showed common p53 DBD residues mediate both 53BP1 and DNA binding and the accompanying prediction that the two events could not occur simultaneously (Joo et al., 2002). While further work will be needed to define the exact mechanism of 53BP1-USP28-p53 interplay, here we provide an integrated model in which p53's transcription-regulating activities are actively enhanced by 53BP1-USP28 protein complexes. This would be consistent with a putative cooperation between p53, 53BP1, and USP28 in tumor suppression. Indeed, our study paves the way for investigations to disentangle 53BP1's roles during p53 and DNA repair regulation to define the relative importance of each function in counteracting cancer.

EXPERIMENTAL PROCEDURES

Cell Lines, Cell Culture, and Genome Editing

All MCF-7 human breast adenocarcinoma cell lines were cultured in DMEM supplemented with 10% FBS, Pen-Strep, and 2 mM L-Glutamine at 37°C in 5% CO_2. *p53Δ*, *53BP1Δ*, *53BP1*^{ΔBRCT}, and *USP28Δ* MCF-7 were generated using the CRISPR-Cas9 system. Briefly, gene-specific gRNAs (sequences in Supplemental Experimental Procedures) were cloned in a modified pX330 vector (Addgene #42230) containing a puromycin resistance cassette. MCF-7 transfected (Fugene HD, Promega) with pX330-puro were enriched by puromycin pulse selection (48 hr, 2 μg/ml), and isogenic clones were isolated by limiting dilution. The presence of gene-disrupting indels in edited cell lines was confirmed by Sanger sequencing, and the ablation of protein production was assessed by immunoblotting and indirect immunofluorescence. Stable-complemented cell lines were generated by lentivirus-mediated transduction, using viral supernatants harvested from 293T cells co-transfected with third generation packaging vectors and pLenti-PGK-PURO-DEST (Addgene #19068) or pHAGE-N-FLAG-HA DEST vectors containing cloned transgene inserts.

Nutlin-3 Sensitivity Assays

Cells seeded in triplicate at 1.25 × 10^4 cells/well in 6-well plates were untreated or treated with 4 μM (±)-Nutlin-3 (Cayman Chemicals) 16 hr later. 7 days after treatment, cells were fixed and stained using crystal violet solution (0.5% [w/v] in 20% methanol). For quantification, bound crystal violet was dissolved in 10% (v/v) acetic acid, and absorbance of 1:50 dilutions was measured at 595 nm.

Immunoprecipitation

Cells lyzed in Benzonase Lysis Buffer (20 mM HEPES [pH 7.9], 40 mM KCl, 2 mM $MgCl_2$, 12% glycerol, 0.5% CHAPS, 50 U/ml Benzonase [Novagen], 0.05% [v/v] phosphatase inhibitors [Sigma-Aldrich] and protease inhibitors

[Roche]) were supplemented with KCL to a 450 mM final concentration and gently mixed for 30 min at 4°C. Clarified lysates were then cassette dialyzed (Slide-A-Lyzer, Thermo Fisher Scientific) in dialysis buffer (20 mM HEPES [pH 7.9], 100 mM KCl, 0.2 mM EDTA, 10% Glycerol, 0.5 mM DTT, 0.5 mM PMSF, 5 mM NaF, 10 mM β-glycerolphosphate). Flag-HA-53BP1 or endogenous p53 complexes were purified from 1–2 mg total protein using anti-FLAG M2 magnetic resin (Sigma-Aldrich) or p53 DO-1 antibody (Santa Cruz Biotechnology) coupled to protein G Dynabeads (Invitrogen). Protein-bead complexes washed extensively in dialysis buffer were either boiled in Laemmli buffer or eluted in 3× Flag peptide (Sigma-Aldrich).

Chromatin Immunoprecipitation

ChIP experiments were performed from 30–50 μg MCF-7 chromatin essentially as previously described (Chapman et al., 2013). Briefly, chromatin was immunoprecipitated using 3 μg anti-p53 (DO-1, Santa Cruz), 1.5 μg anti-RNAP2 CTD (phospho-S5; Clone 4H8), 2.5μg Anti-Histone H3 (acetyl K9) (ab4441, Abcam), or 2.5 μg anti-acetyl-histone H4 (06-598, Millipore) antibody coupled to 25 μl Protein-A/G Dynabeads (Life Technologies). DNA quantities recovered in control IgG ChIP experiments were consistently below the detectable range. Relative quantities of ChIP-enriched DNA were calculated relative to total input chromatin by qPCR in triplicate on a CFX96 Real-Time Analyzer (Bio-Rad) using Quantifast SYBR Green reagent (QIAGEN) and locus-specific primer pairs (sequences in Supplemental Experimental Procedures).

ACCESSION NUMBERS

The accession number for RNA-seq data reported in this paper is NCBI GEO: GSE84986.

SUPPLEMENTAL INFORMATION

Supplemental Information includes Supplemental Experimental Procedures, seven figures, and one table and can be found with this article online at http://dx.doi.org/10.1016/j.molcel.2016.08.002.

AUTHOR CONTRIBUTIONS

J.R.C. conceived and supervised the study and wrote the manuscript. J.R.C. and R.C.-M. designed the experiments. R.C.-M. performed the majority of the experiments with assistance from C.O., N.G., S.S., and J.R.C. H.E.L. analyzed all NGS datasets.

ACKNOWLEDGMENTS

We thank all J.R.C. lab members for input and Catherine Green for comments on the manuscript. We also thank B. Davies for reagents, H. Castillo Ècija, L. Stokes, and the WTCHG Chromosome Dynamics core for assistance in cell-line generation and characterization, C. Siebold for structural advice, and the WTCHG High Throughput Genomics core for NGS. Research in the J.R.C. group is supported by a Cancer Research UK (CRUK) Career Development Fellowship awarded to J.R.C. (C52690/A19270), CRUK grant number C5255/A18085, and Wellcome Trust grant (090532/Z/09/Z). R.C.-M. is supported by a Nuffield Department of Medicine Prize DPhil studentship and S.S. by a Marie Skłodowska-Curie Actions Individual Fellowship from the European Commission (H2020-MSCA-IF-2014-656357).

Received: June 15, 2016
Revised: July 8, 2016
Accepted: July 29, 2016
Published: August 18, 2016

REFERENCES

Baldock, R.A., Day, M., Wilkinson, O.J., Cloney, R., Jeggo, P.A., Oliver, A.W., Watts, F.Z., and Pearl, L.H. (2015). ATM Localization and Heterochromatin Repair Depend on Direct Interaction of the 53BP1-BRCT2 Domain with γH2AX. Cell Rep. *13*, 2081–2089.

Bothmer, A., Robbiani, D.F., Di Virgilio, M., Bunting, S.F., Klein, I.A., Feldhahn, N., Barlow, J., Chen, H.-T., Bosque, D., Callen, E., et al. (2011). Regulation of DNA end joining, resection, and immunoglobulin class switch recombination by 53BP1. Mol. Cell *42*, 319–329.

Botuyan, M.V., Lee, J., Ward, I.M., Kim, J.-E., Thompson, J.R., Chen, J., and Mer, G. (2006). Structural basis for the methylation state-specific recognition of histone H4-K20 by 53BP1 and Crb2 in DNA repair. Cell *127*, 1361–1373.

Bouwman, P., Aly, A., Escandell, J.M., Pieterse, M., Bartkova, J., van der Gulden, H., Hiddingh, S., Thanasoula, M., Kulkarni, A., Yang, Q., et al. (2010). 53BP1 loss rescues BRCA1 deficiency and is associated with triple-negative and BRCA-mutated breast cancers. Nat. Struct. Mol. Biol. *17*, 688–695.

Brooks, C.L., and Gu, W. (2006). p53 ubiquitination: Mdm2 and beyond. Mol. Cell *21*, 307–315.

Brummelkamp, T.R., Fabius, A.W.M., Mullenders, J., Madiredjo, M., Velds, A., Kerkhoven, R.M., Bernards, R., and Beijersbergen, R.L. (2006). An shRNA barcode screen provides insight into cancer cell vulnerability to MDM2 inhibitors. Nat. Chem. Biol. *2*, 202–206.

Bunting, S.F., Callén, E., Wong, N., Chen, H.-T., Polato, F., Gunn, A., Bothmer, A., Feldhahn, N., Fernandez-Capetillo, O., Cao, L., et al. (2010). 53BP1 inhibits homologous recombination in Brca1-deficient cells by blocking resection of DNA breaks. Cell *141*, 243–254.

Callen, E., Di Virgilio, M., Kruhlak, M.J., Nieto-Soler, M., Wong, N., Chen, H.-T., Faryabi, R.B., Polato, F., Santos, M., Starnes, L.M., et al. (2013). 53BP1 mediates productive and mutagenic DNA repair through distinct phosphoprotein interactions. Cell *153*, 1266–1280.

Carr, S.M., Munro, S., Zalmas, L.-P., Fedorov, O., Johansson, C., Krojer, T., Sagum, C.A., Bedford, M.T., Oppermann, U., and La Thangue, N.B. (2014). Lysine methylation-dependent binding of 53BP1 to the pRb tumor suppressor. Proc. Natl. Acad. Sci. USA *111*, 11341–11346.

Cescutti, R., Negrini, S., Kohzaki, M., and Halazonetis, T.D. (2010). TopBP1 functions with 53BP1 in the G1 DNA damage checkpoint. EMBO J. *29*, 3723–3732.

Chapman, J.R., Taylor, M.R.G., and Boulton, S.J. (2012). Playing the end game: DNA double-strand break repair pathway choice. Mol. Cell *47*, 497–510.

Chapman, J.R., Barral, P., Vannier, J.-B., Borel, V., Steger, M., Tomas-Loba, A., Sartori, A.A., Adams, I.R., Batista, F.D., and Boulton, S.J. (2013). RIF1 is essential for 53BP1-dependent nonhomologous end joining and suppression of DNA double-strand break resection. Mol. Cell *49*, 858–871.

Cummins, J.M., Rago, C., Kohli, M., Kinzler, K.W., Lengauer, C., and Vogelstein, B. (2004). Tumour suppression: disruption of HAUSP gene stabilizes p53. Nature *428*, 1–486.

Derbyshire, D.J., Basu, B.P., Serpell, L.C., Joo, W.S., Date, T., Iwabuchi, K., and Doherty, A.J. (2002). Crystal structure of human 53BP1 BRCT domains bound to p53 tumour suppressor. EMBO J. *21*, 3863–3872.

Di Virgilio, M., Callen, E., Yamane, A., Zhang, W., Jankovic, M., Gitlin, A.D., Feldhahn, N., Resch, W., Oliveira, T.Y., Chait, B.T., et al. (2013). Rif1 prevents resection of DNA breaks and promotes immunoglobulin class switching. Science *339*, 711–715.

Difilippantonio, S., Gapud, E., Wong, N., Huang, C.-Y., Mahowald, G., Chen, H.-T., Kruhlak, M.J., Callen, E., Livak, F., Nussenzweig, M.C., et al. (2008). 53BP1 facilitates long-range DNA end-joining during V(D)J recombination. Nature *456*, 529–533.

Donner, A.J., Szostek, S., Hoover, J.M., and Espinosa, J.M. (2007). CDK8 is a stimulus-specific positive coregulator of p53 target genes. Mol. Cell *27*, 121–133.

Dulić, V., Kaufmann, W.K., Wilson, S.J., Tlsty, T.D., Lees, E., Harper, J.W., Elledge, S.J., and Reed, S.I. (1994). p53-dependent inhibition of cyclin-dependent kinase activities in human fibroblasts during radiation-induced G1 arrest. Cell *76*, 1013–1023.

Escribano-Díaz, C., Orthwein, A., Fradet-Turcotte, A., Xing, M., Young, J.T.F., Tkáč, J., Cook, M.A., Rosebrock, A.P., Munro, M., Canny, M.D., et al. (2013). A cell cycle-dependent regulatory circuit composed of 53BP1-RIF1 and BRCA1-CtIP controls DNA repair pathway choice. Mol. Cell *49*, 872–883.

Fradet-Turcotte, A., Canny, M.D., Escribano-Díaz, C., Orthwein, A., Leung, C.C.Y., Huang, H., Landry, M.-C., Kitevski-LeBlanc, J., Noordermeer, S.M., Sicheri, F., and Durocher, D. (2013). 53BP1 is a reader of the DNA-damage-induced H2A Lys 15 ubiquitin mark. Nature *499*, 50–54.

Huang, J., Sengupta, R., Espejo, A.B., Lee, M.G., Dorsey, J.A., Richter, M., Opravil, S., Shiekhattar, R., Bedford, M.T., Jenuwein, T., and Berger, S.L. (2007). p53 is regulated by the lysine demethylase LSD1. Nature *449*, 105–108.

Iwabuchi, K., Bartel, P.L., Li, B., Marraccino, R., and Fields, S. (1994). Two cellular proteins that bind to wild-type but not mutant p53. Proc. Natl. Acad. Sci. USA *91*, 6098–6102.

Iwabuchi, K., Li, B., Massa, H.F., Trask, B.J., Date, T., and Fields, S. (1998). Stimulation of p53-mediated transcriptional activation by the p53-binding proteins, 53BP1 and 53BP2. J. Biol. Chem. *273*, 26061–26068.

Iwabuchi, K., Hashimoto, M., Matsui, T., Kurihara, T., Shimizu, H., Adachi, N., Ishiai, M., Yamamoto, K., Tauchi, H., Takata, M., et al. (2006). 53BP1 contributes to survival of cells irradiated with X-ray during G1 without Ku70 or Artemis. Genes Cells *11*, 935–948.

Joo, W.S., Jeffrey, P.D., Cantor, S.B., Finnin, M.S., Livingston, D.M., and Pavletich, N.P. (2002). Structure of the 53BP1 BRCT region bound to p53 and its comparison to the Brca1 BRCT structure. Genes Dev. *16*, 583–593.

Kachirskaia, I., Shi, X., Yamaguchi, H., Tanoue, K., Wen, H., Wang, E.W., Appella, E., and Gozani, O. (2008). Role for 53BP1 Tudor domain recognition of p53 dimethylated at lysine 382 in DNA damage signaling. J. Biol. Chem. *283*, 34660–34666.

Kleiner, R.E., Verma, P., Molloy, K.R., Chait, B.T., and Kapoor, T.M. (2015). Chemical proteomics reveals a γH2AX-53BP1 interaction in the DNA damage response. Nat. Chem. Biol. *11*, 807–814.

Knobel, P.A., Belotserkovskaya, R., Galanty, Y., Schmidt, C.K., Jackson, S.P., and Stracker, T.H. (2014). USP28 is recruited to sites of DNA damage by the tandem BRCT domains of 53BP1 but plays a minor role in double-strand break metabolism. Mol. Cell. Biol. *34*, 2062–2074.

Krenning, L., Feringa, F.M., Shaltiel, I.A., van den Berg, J., and Medema, R.H. (2014). Transient activation of p53 in G2 phase is sufficient to induce senescence. Mol. Cell *55*, 59–72.

Kruse, J.-P., and Gu, W. (2009). Modes of p53 regulation. Cell *137*, 609–622.

Lee, J.-H., Goodarzi, A.A., Jeggo, P.A., and Paull, T.T. (2010). 53BP1 promotes ATM activity through direct interactions with the MRN complex. EMBO J. *29*, 574–585.

Li, M., Chen, D., Shiloh, A., Luo, J., Nikolaev, A.Y., Qin, J., and Gu, W. (2002). Deubiquitination of p53 by HAUSP is an important pathway for p53 stabilization. Nature *416*, 648–653.

Li, M., Brooks, C.L., Kon, N., and Gu, W. (2004). A dynamic role of HAUSP in the p53-Mdm2 pathway. Mol. Cell *13*, 879–886.

Liao, M.J., Zhang, X.X., Hill, R., Gao, J., Qumsiyeh, M.B., Nichols, W., and Van Dyke, T. (1998). No requirement for V(D)J recombination in p53-deficient thymic lymphoma. Mol. Cell. Biol. *18*, 3495–3501.

Lottersberger, F., Bothmer, A., Robbiani, D.F., Nussenzweig, M.C., and de Lange, T. (2013). Role of 53BP1 oligomerization in regulating double-strand break repair. Proc. Natl. Acad. Sci. USA *110*, 2146–2151.

Lowe, S.W., Schmitt, E.M., Smith, S.W., Osborne, B.A., and Jacks, T. (1993). p53 is required for radiation-induced apoptosis in mouse thymocytes. Nature *362*, 847–849.

Manis, J.P., Morales, J.C., Xia, Z., Kutok, J.L., Alt, F.W., and Carpenter, P.B. (2004). 53BP1 links DNA damage-response pathways to immunoglobulin heavy chain class-switch recombination. Nat. Immunol. *5*, 481–487.

Morales, J.C., Franco, S., Murphy, M.M., Bassing, C.H., Mills, K.D., Adams, M.M., Walsh, N.C., Manis, J.P., Rassidakis, G.Z., Alt, F.W., and Carpenter, P.B. (2006). 53BP1 and p53 synergize to suppress genomic instability and lymphomagenesis. Proc. Natl. Acad. Sci. USA *103*, 3310–3315.

Noon, A.T., Shibata, A., Rief, N., Löbrich, M., Stewart, G.S., Jeggo, P.A., and Goodarzi, A.A. (2010). 53BP1-dependent robust localized KAP-1 phosphorylation is essential for heterochromatic DNA double-strand break repair. Nat. Cell Biol. *12*, 177–184.

Reinhardt, H.C., and Yaffe, M.B. (2013). Phospho-Ser/Thr-binding domains: navigating the cell cycle and DNA damage response. Nat. Rev. Mol. Cell Biol. *14*, 563–580.

Samuels-Lev, Y., O'Connor, D.J., Bergamaschi, D., Trigiante, G., Hsieh, J.K., Zhong, S., Campargue, I., Naumovski, L., Crook, T., and Lu, X. (2001). ASPP proteins specifically stimulate the apoptotic function of p53. Mol. Cell *8*, 781–794.

Toledo, F., and Wahl, G.M. (2006). Regulating the p53 pathway: in vitro hypotheses, in vivo veritas. Nat. Rev. Cancer *6*, 909–923.

Vassilev, L.T., Vu, B.T., Graves, B., Carvajal, D., Podlaski, F., Filipovic, Z., Kong, N., Kammlott, U., Lukacs, C., Klein, C., et al. (2004). In vivo activation of the p53 pathway by small-molecule antagonists of MDM2. Science *303*, 844–848.

Vousden, K.H., and Prives, C. (2009). Blinded by the Light: The Growing Complexity of p53. Cell *137*, 413–431.

Ward, I.M., Reina-San-Martin, B., Olaru, A., Minn, K., Tamada, K., Lau, J.S., Cascalho, M., Chen, L., Nussenzweig, A., Livak, F., et al. (2004). 53BP1 is required for class switch recombination. J. Cell Biol. *165*, 459–464.

Ward, I.M., Difilippantonio, S., Minn, K., Mueller, M.D., Molina, J.R., Yu, X., Frisk, C.S., Ried, T., Nussenzweig, A., and Chen, J. (2005). 53BP1 cooperates with p53 and functions as a haploinsufficient tumor suppressor in mice. Mol. Cell. Biol. *25*, 10079–10086.

Ward, I., Kim, J.-E., Minn, K., Chini, C.C., Mer, G., and Chen, J. (2006). The tandem BRCT domain of 53BP1 is not required for its repair function. J. Biol. Chem. *281*, 38472–38477.

Zgheib, O., Pataky, K., Brugger, J., and Halazonetis, T.D. (2009). An oligomerized 53BP1 tudor domain suffices for recognition of DNA double-strand breaks. Mol. Cell. Biol. *29*, 1050–1058.

Zhang, D., Zaugg, K., Mak, T.W., and Elledge, S.J. (2006). A role for the deubiquitinating enzyme USP28 in control of the DNA-damage response. Cell *126*, 529–542.

Zimmermann, M., and de Lange, T. (2014). 53BP1: pro choice in DNA repair. Trends Cell Biol. *24*, 108–117.

Zimmermann, M., Lottersberger, F., Buonomo, S.B., Sfeir, A., and de Lange, T. (2013). 53BP1 regulates DSB repair using Rif1 to control 5′ end resection. Science *339*, 700–704.

Molecular Cell, Volume *64*

Supplemental Information

53BP1 Integrates DNA Repair and p53-Dependent Cell Fate Decisions via Distinct Mechanisms

Raquel Cuella-Martin, Catarina Oliveira, Helen E. Lockstone, Suzanne Snellenberg, Natalia Grolmusova, and J. Ross Chapman

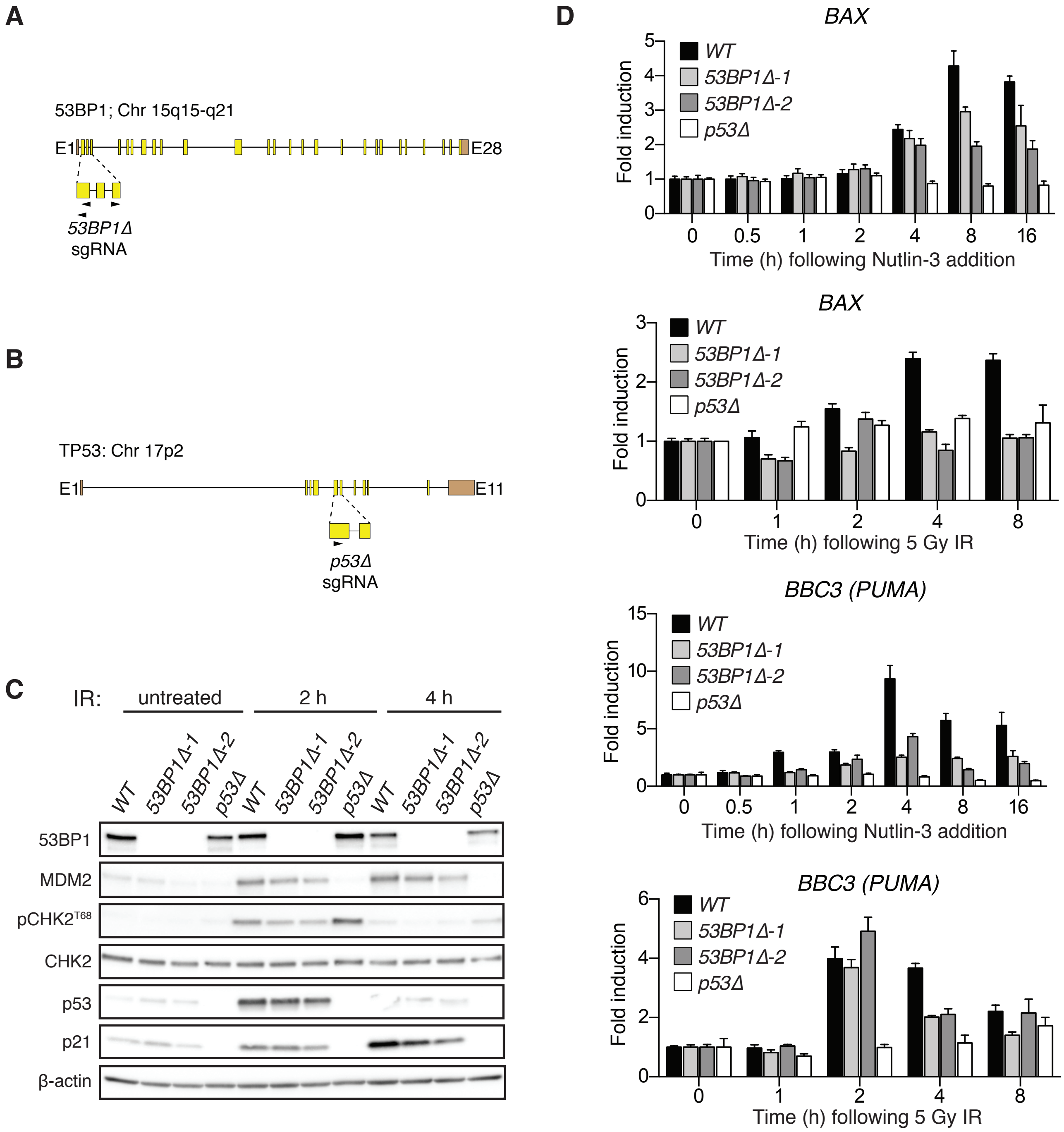

A
53BP1; Chr 15q15-q21
E1
E28
53BP1Δ
sgRNA
B
TP53: Chr 17p2
E1
E11
p53Δ
sgRNA
C
IR:
untreated
2 h
4 h
WT
53BP1Δ-1
53BP1Δ-2
p53Δ
53BP1
MDM2
pCHK2T68
CHK2
p53
p21
β-actin
D
BAX
Fold induction
Time (h) following Nutlin-3 addition
Time (h) following 5 Gy IR
BBC3 (PUMA)

Figure S1

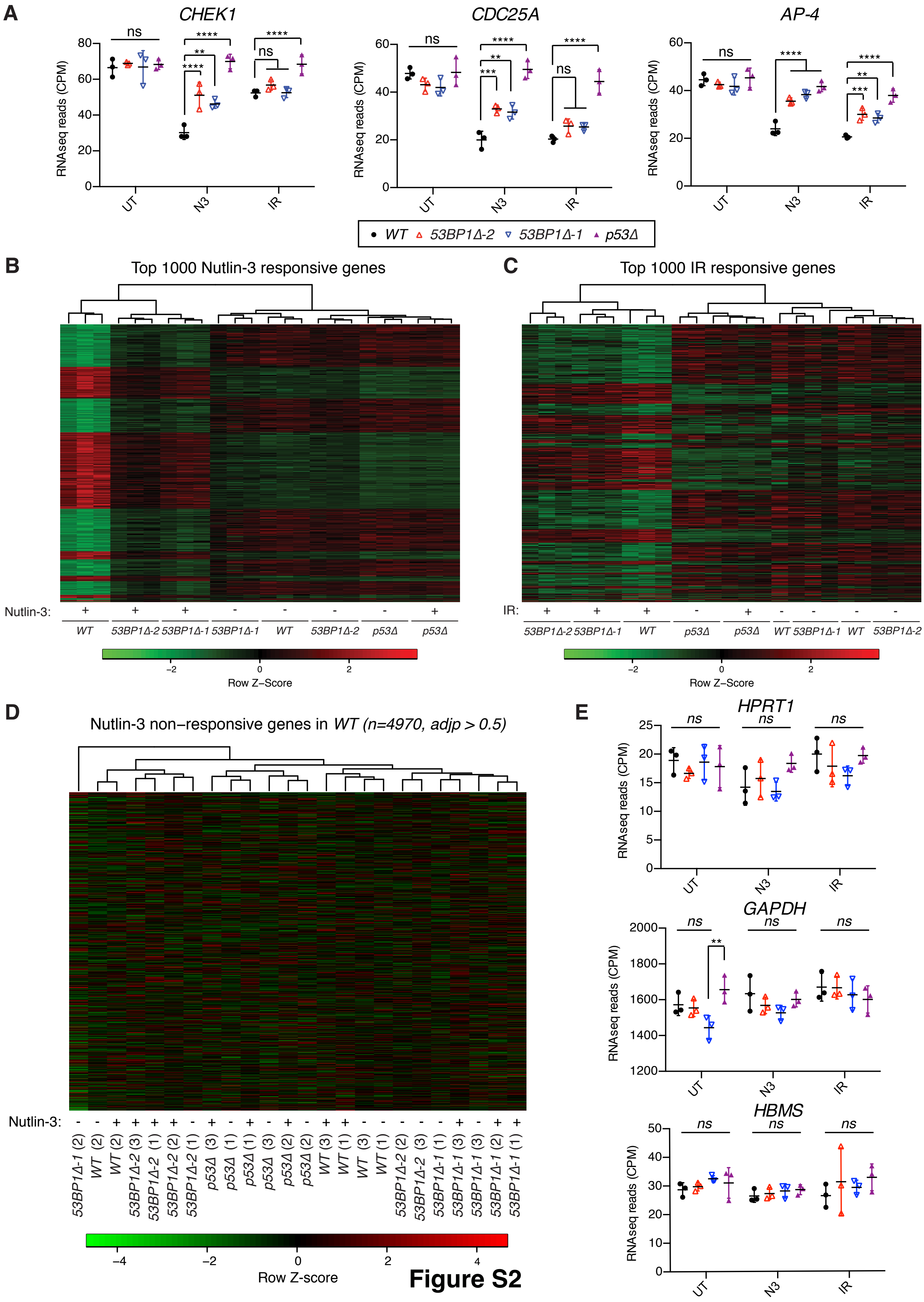
A
CHEK1
CDC25A
AP-4
RNAseq reads (CPM)
UT
N3
IR
ns
WT 53BP1Δ-2 53BP1Δ-1 p53Δ
B
Top 1000 Nutlin-3 responsive genes
Nutlin-3:
WT 53BP1Δ-2 53BP1Δ-1 53BP1Δ-1 WT 53BP1Δ-2 p53Δ p53Δ
Row Z–Score
C
Top 1000 IR responsive genes
IR:
53BP1Δ-2 53BP1Δ-1 WT p53Δ p53Δ WT 53BP1Δ-1 WT 53BP1Δ-2
Row Z–Score
D
Nutlin-3 non–responsive genes in WT (n=4970, adjp > 0.5)
Nutlin-3:
Row Z-score
E
HPRT1
GAPDH
HBMS

Figure S2

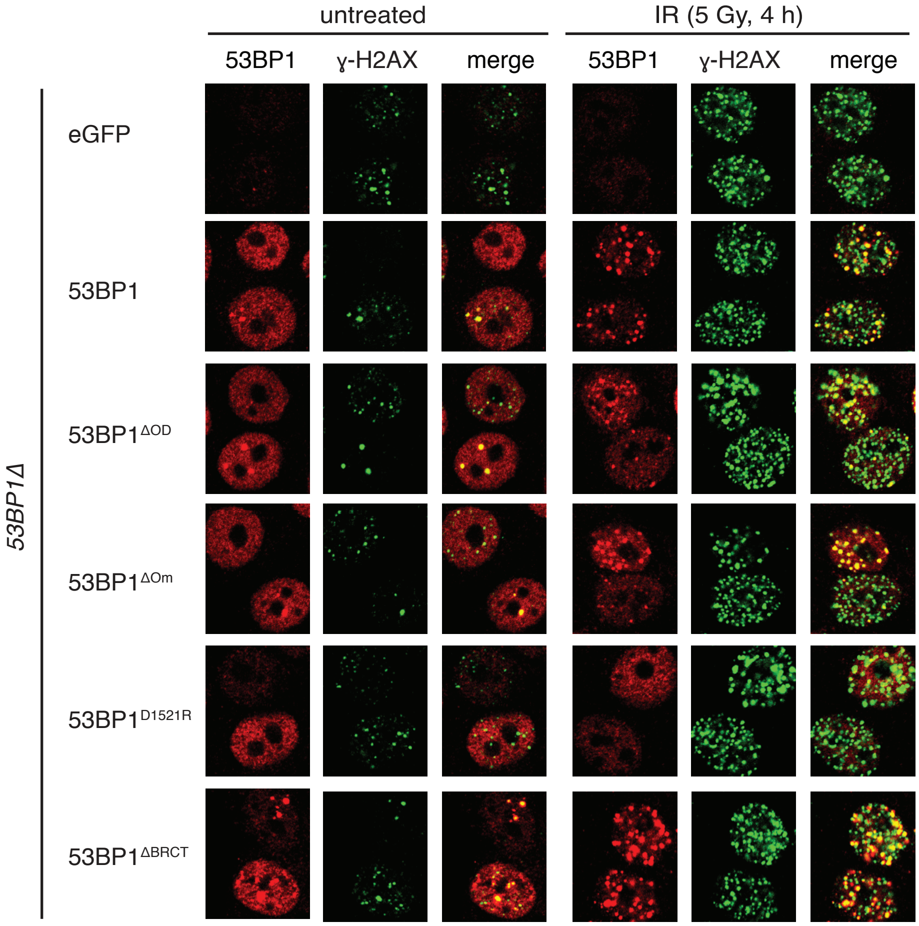
untreated
IR (5 Gy, 4 h)
53BP1
γ-H2AX
merge
53BP1
γ-H2AX
merge
53BP1Δ
eGFP
53BP1
53BP1ΔOD
53BP1ΔOm
53BP1D1521R
53BP1ΔBRCT

Figure S3

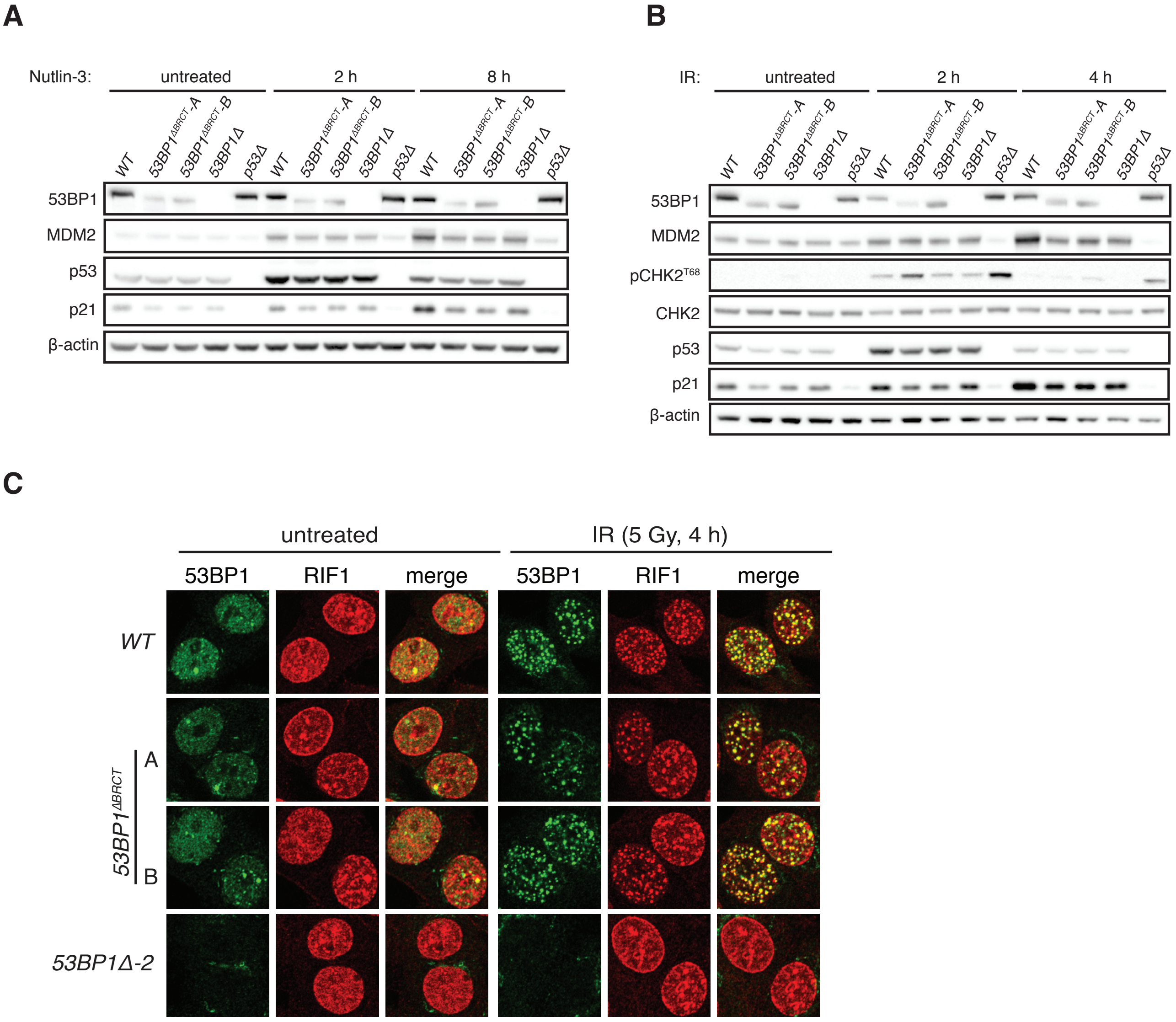
A
Nutlin-3:
untreated
2 h
8 h
WT
53BP1ΔBRCT-A
53BP1ΔBRCT-B
53BP1Δ
p53Δ
53BP1
MDM2
p53
p21
β-actin
B
IR:
untreated
2 h
4 h
53BP1
MDM2
pCHK2T68
CHK2
p53
p21
β-actin
C
untreated
IR (5 Gy, 4 h)
53BP1
RIF1
merge
WT
53BP1ΔBRCT
A
B
53BP1Δ-2

Figure S4

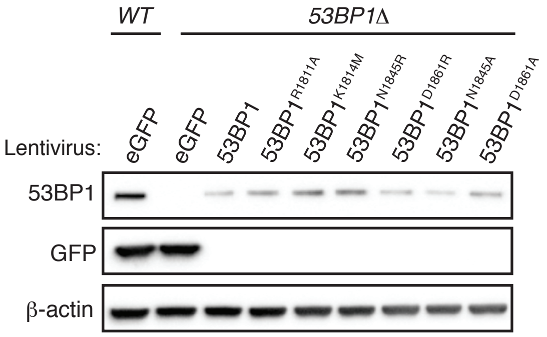
WT
53BP1Δ
Lentivirus:
eGFP
eGFP
53BP1
53BP1R1811A
53BP1K1814M
53BP1N1845R
53BP1D1861R
53BP1N1845A
53BP1D1861A
53BP1
GFP
β-actin

Figure S5

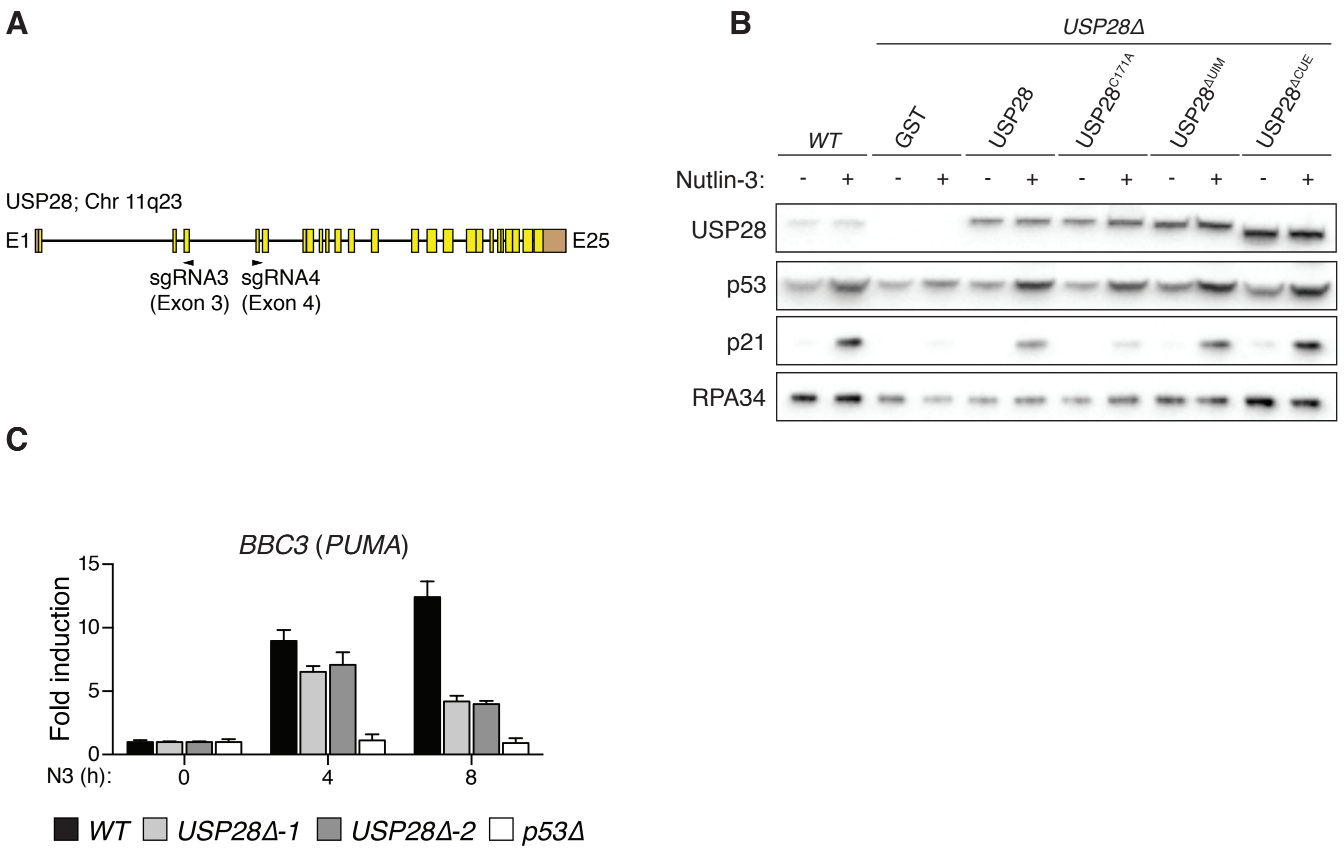
A
USP28; Chr 11q23
E1
E25
sgRNA3
(Exon 3)
sgRNA4
(Exon 4)
B
USP28Δ
WT
GST
USP28
USP28C171A
USP28ΔUIM
USP28ΔCUE
Nutlin-3:
USP28
p53
p21
RPA34
C
BBC3 (PUMA)
Fold induction
15
10
5
0
N3 (h):
0
4
8
WT
USP28Δ-1
USP28Δ-2
p53Δ

Figure S6

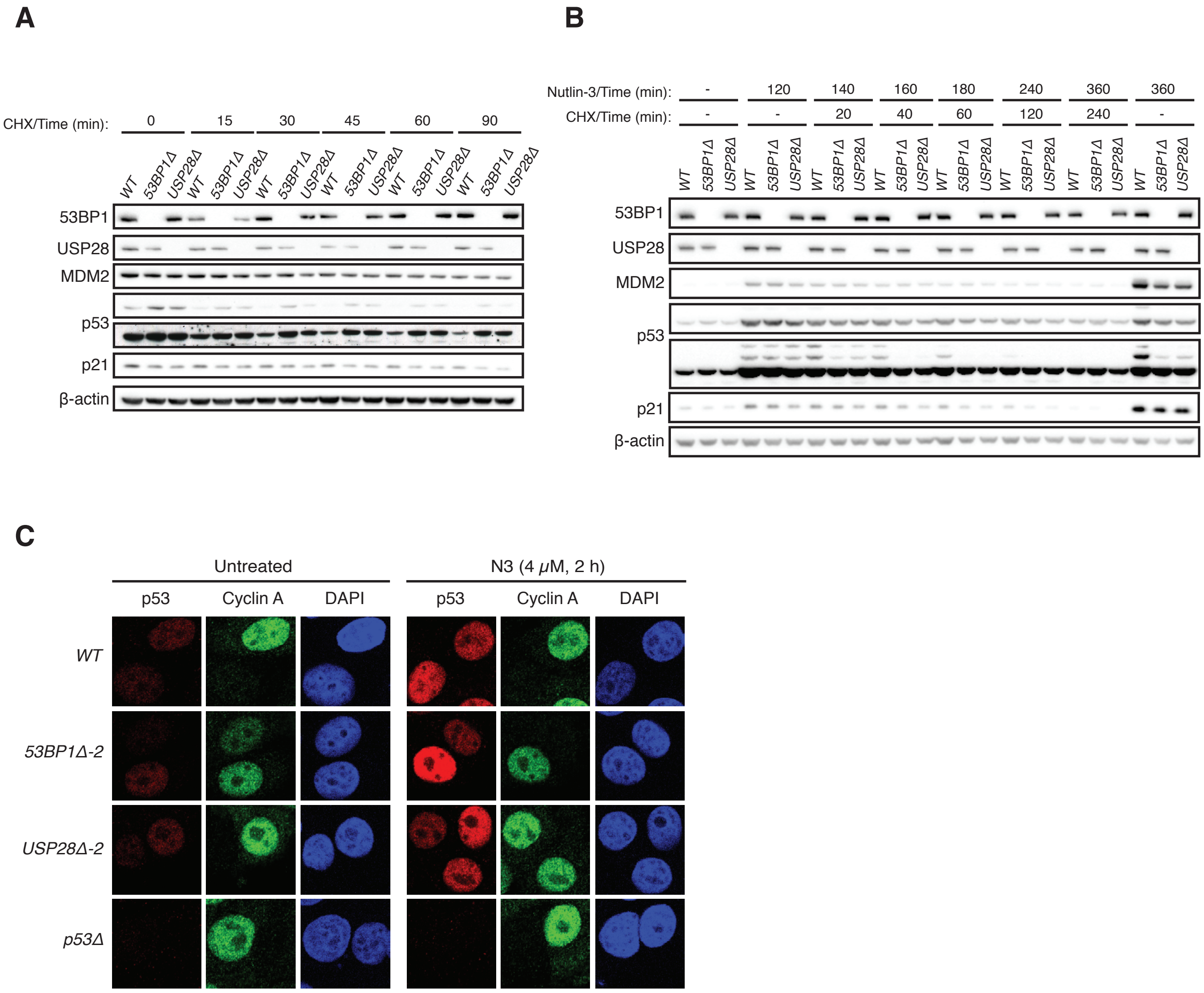
A
CHX/Time (min):
0
15
30
45
60
90
WT
53BP1Δ
USP28Δ
53BP1
USP28
MDM2
p53
p21
β-actin
B
Nutlin-3/Time (min):
-
120
140
160
180
240
360
360
CHX/Time (min):
-
-
20
40
60
120
240
-
WT
53BP1Δ
USP28Δ
53BP1
USP28
MDM2
p53
p21
β-actin
C
Untreated
N3 (4 μM, 2 h)
p53
Cyclin A
DAPI
WT
53BP1Δ-2
USP28Δ-2
p53Δ

Figure S7

SUPPLEMENTARY FIGURE LEGENDS

Supplementary Figure S1, related to Figure 1. 53BP1 is required for optimal p53-dependent transactivation events.

Schematic representations of the human *53BP1* **(A)** and *TP53* **(B)** loci, illustrating the hybridization sites of the gRNAs selected for the generation of *53BP1*Δ and *p53*Δ cell-lines in this study (gRNA sequences in Supplementary Table S1). **(C)** Immunoblot analysis of lysates prepared from untreated or IR-treated (5 Gy) cells of indicated genotype. **(D)** 53BP1-loss impairs IR-induced p53-dependent transactivation events. Total RNA was purified from cells treated with N3 (4 μM) or IR (5 Gy) and monitored for the abundance of indicated p53-responsive transcripts by RT-qPCR. Data is representative of two independent experiments, mean ± SD.

Supplementary Figure S2, related to Figure 1. 53BP1 is an enhancer of p53-dependent transcriptional programs.

(A) 53BP1 is required for p53-dependent transcriptional repression events. Representative p53-responsive transcripts from three RNA-seq replicates. Total RNA was sequenced from indicated MCF-7 lines following N3 (4 μM, 8 h), IR (5 Gy, 4 h) or control treatments. CPM, counts per million; *ns*, non significant; *$p<0.05$; **$p<0.01$; ***$p<0.001$; ****$p<0.001$ (two-way ANOVA). Bars represent mean ± SD. **(B)**, **(C)** RNA-seq results and reproducibility across biological replicates. Heatmaps showing relative expression levels for each independent biological replicate of each condition for the top 1000 treatment-responsive genes, in respect to the untreated control samples. Unsupervised hierarchical clustering was used to cluster the samples based on expression levels of the respective set of 1000 genes for N3 and IR treatments. **(D)** Normal expression of Nutlin-3 non-responsive genes in *53BP1*Δ and *p53*Δ MCF-7. Heatmaps showing relative expression levels for each independent biological replicate of each condition for the 4970 treatment non-responsive genes in *WT* MCF-7, in respect to the untreated control samples. Unsupervised hierarchical clustering was used to cluster the samples based on expression levels of the respective set of 4970 genes for N3 and IR treatments. **(E)** As in **A**, but for three commonly employed reference transcripts.

Supplementary Figure S3, related to Figure 2. Expected nuclear localization patterns of N3-response defective 53BP1 mutants.

The localization and IR-dependent recruitment patterns of the indicated 53BP1 mutant proteins was analyzed in stably transduced *53BP1*Δ MCF-7 lines following mock or IR (5Gy 4 h) treatment. Cells were fixed, immunostained with indicated antibodies, and then processed by indirect immunofluorescence.

Notably, 53BP1 oligomerization mutant proteins formed residual IRIF, consistent with a previous report (Lottersberger et al., 2013).

Supplementary Figure S4, related to Figure 3. 53BP1 dependent p53 regulation and DSB repair activities are distinct and separable.

(A), (B) *53BP1*$^{\Delta BRCT}$ cells show reduced MDM2 and p21 induction following N3 and IR treatments. Western blot analysis of lysates prepared from indicated cell-lines following treatment with 4 µM N3 (A) or 5 Gy IR (B) relative to untreated control samples. **(C)** Unlike *53BP1*Δ cells, *53BP1*$^{BRCT\Delta}$ cells are proficient in supporting RIF1 recruitment into IR-induced foci. Cells mock-treated or irradiated (5 Gy, 4 h), were fixed, immunostained with indicated antibodies, and processed by indirect immunofluorescence.

Supplementary Figure S5, related to Figure 4. The 53BP1 BRCT domain mediates bivalent interactions with p53 and USP28.

Western blot showing stable expression of indicated WT and 53BP1 BRCT mutants in lentivirus-transduced *53BP1Δ* cells.

Supplementary Figure S6, related to Figure 5. USP28 is a novel component of the p53-53BP1 axis.

(A) Schematic representation of the human *USP28* locus depicting the hybridization sites for each gRNA used to generate *USP28Δ* cell-lines (gRNA sequences in supplementary Table S1). **(B)** USP28 catalytic activity is required for its p53-regulatory role. Indicated WT and mutant USP28 transgenes were stably expresses in *USP28Δ* cells following lentivirus-mediated transduction. Lysates were prepared from cells following treatment with N3 (4 µM, 8 h), and immunoblotted with indicated antibodies. **(C)** USP28-loss impairs N3-induced p53-dependent PUMA transactivation. Total RNA was purified from cells treated with N3 (4 µM) and monitored for the abundance of BBC3 (PUMA) transcript for the indicated times. Data is representative of two independent experiments, mean ± SD.

Supplementary Figure S7, related to Figure 7. Normal p53 stability and localization in *53BP1*Δ and *USP28*Δ cells.

(A) Steady-state p53 half-life in WT, *53BP1*Δ and *USP28*Δ cells. p53 half-life analysis was performed in time-course experiments following cyclohexamide addition to halt *de novo* protein synthesis. Cell lysates prepared at indicated time-points following cycloheximide addition were immunoblotted with indicated antibodies. **(B)** p53 half-life in N3-treated WT, *53BP1*Δ and *USP28*Δ cells. Similar to B, except cycloheximide was added 2 h following p53-activation by N3-treatment. **(C)** Normal p53 nuclear localization in WT, *53BP1*Δ and *USP28*Δ cells. Cells were treated with 4 µM N3 for 2 h or left untreated,

were fixed and immunostained with the monoclonal p53 DO-7 antibody. Cyclin A-counterstaining enabled discrimination of G1 (Cyclin A negative) and S/G2 (Cyclin A positive) cell populations, and provided no evidence for cell-cycle-dependent defects in p53 localization.

	**Gene #**	**Nutlin-3**	**IR**
WT	Activated	3559	1043
	Repressed	3318	1343
53BP1Δ-1	Activated	1143	497
	Repressed	1096	686
53BP1Δ-2	Activated	1366	551
	Repressed	1624	767
p53Δ	Activated	10	6
	Repressed	3	19

Supplementary Table S1, related to Figure 1. Number of genes showing significant changes (*adjusted p-value <0.05*) in RNA-seq experiments upon N3 and IR treatments with respect to the corresponding untreated control.

EXTENDED EXPERIMENTAL PROCEDURES

Protein analysis: SDS/PAGE-Western Blot

Whole cell protein extracts were isolated using Benzonase buffer [25 mM Tris (pH 8.0), 40 mM NaCl, 0.05% SDS, 2 mM $MgCl_2$, 10 U/ml Benzonase (Sigma-Aldrich), 0.05% (v/v) phosphatase inhibitor cocktail 3 (P5726; Sigma-Aldrich) and protease inhibitors (Complete, Roche)], diluted in 3X Laemmli buffer and boiled. SDS-PAGE was performed using NuPAGE® Novex® (Life Technologies) or Criterion® (Bio-Rad) gradient acrylamide gels before transfer onto 0.45 μM nitrocellulose membranes (Life Technologies). Primary antibodies used in this study are listed below. Proteins were detected using HRP-conjugated secondary antibodies and enhanced chemiluminescence (Clarity, Bio-Rad). Signals were acquired digitally on a Gel Doc™ XR system (Bio-Rad).

Immunofluorescence assays

Cells were seeded in coverslips in 6-well plates at a cell density of $4x10^5$ cells/well. After treatments, cells were fixed in a 2% *p*-formaldehyde solution and permeabilized with 0.2% (v/v) Triton-X100 in PBS. Coverslips were blocked in 3% (w/v) BSA/0.1% (v/v) Triton-X100 in PBS prior to sequential incubations with primary (listed below) and secondary antibodies (Alexa Fluor 488, 594 and 648; Molecular Probes). Images were captured in a confocal scanning microscope (LSM 510 Meta, Zeiss).

Clonogenic IR survival assay

The sensitivity of cells plated on 10 cm dishes in triplicate and exposed to indicated X-ray doses (CellRad, Faxitron), was assessed relative to non-irradiated control plates. Fourteen days after treatment, plates were stained using crystal violet dye and colonies counted.

Real time quantitative PCR (RT-qPCR)

Gene expression was determined by RT-qPCR. Trizol/chloroform-extracted total RNA was further purified with the RNeasy Mini Kit (Qiagen) incorporating a DNaseI step (Qiagen) to remove DNA contamination. cDNA was generated from 1 μg of total RNA using the iScript® cDNA Synthesis Kit (Bio-Rad). qPCR was carried out using QuantiFast SYBR Green Master Mix (Qiagen) and transcript-specific primer pairs (sequences listed below). PCR reactions were analysed on a CFX96® Real Time analyzer (Bio-Rad) with the following conditions: enzyme activation-5 min 95 ^oC; 40 cycles denaturation-10 s 95 ^oC annealing/extension-30 s 60 ^oC; final melting curve-15s 65 ^oC, 15 s 95 ^oC. Cycle threshold values (C_T) were used to perform quantification and analysis using CFX Manager software (Bio-Rad).

Hypoxanthine-guanine phosphoribosyl-transferase 1 (*HPRT1*) was used as housekeeping gene for normalization. All values were presented as fold-changes compared to the appropriate control.

Whole transcriptome analysis: RNA-seq

Libraries prepared from ribosomal-RNA depleted total RNA isolates (Ribo-Zero rRNA removal kit, Illumina) were subjected to RNA-seq analysis. Results presented are based on three biological experimental replicates for each condition and genotype. Briefly, sample preparation was carried out according to Illumina guidelines using in-house adapters for library preparation (Lamble et al., 2013). Library quantification and quality control was performed using Picogreen and Tapestation measurements, and equimolar quantities of each library pooled into a 36-plex. 36-plex pools were sequenced (100 bp paired-end reads) across two lanes of a HiSeq4000 sequencer (Illumina). Following subtraction of low-quality reads and duplicate reads (Picard Tools MarkDuplicates), between 10-15 million high-quality reads per sample were analyzed and aligned to the human reference genome (GRCh37), using TopHat2 (Kim et al., 2013). Counts for Ensembl-annotated genes were summarised from the mapped reads, and filtered to exclude genes with fewer than 10 reads on average per sample. Analysis for differential expression was performed using the edgeR package (Robinson et al., 2010). All raw RNA-sequencing datasets generated in this study will be archived and made publically available at the European Nucleotide Archive upon publication.

Immunoprecipitation

Cells initially lyzed in Benzonase Lysis Buffer [20 mM HEPES (pH 7.9), 40 mM KCl, 2 mM $MgCl_2$, 12% glycerol, 0.5% CHAPS, 50 U/ml Benzonase (Novagen), 0.05 % (v/v) phosphatase inhibitors (P0044 and P5726; Sigma-Aldrich) and protease inhibitors (Complete, Roche)], were supplemented with KCL to a 450 mM final concentration and gently mixed for 30 min at 4˚C. Following clarification by centrifugation, lysates were then cassette dialyzed (Slide-A-Lyzer™ MINI, Thermo Fisher Scientific) into dialysis buffer [20 mM HEPES (pH 7.9), 100 mM KCl, 0.2 mM EDTA, 10 % Glycerol, 0.5 mM DTT, 0.5 mM PMSF, 5 mM NaF, 10 mM b-glycerolphosphate]. Flag-HA-53BP1 or endogenous p53 complexes were purified from 1-2 mg total protein using anti-FLAG® M2 magnetic resin (Sigma-Aldrich) or p53 DO-1 antibody (Santa Cruz Biotechnology) coupled to protein G Dynabeads (Invitrogen). Magnetically purified protein-bead complexes washed extensively in dialysis buffer were either boiled in Laemmli buffer or eluted in 3X Flag peptide (Sigma-Aldrich) according to manufacturers instructions.

G1/S checkpoint analysis

The G1/S checkpoint was assessed by BrdU incorporation. Briefly, cells were synchronised in G0 by serum starvation for 24 h and further released in medium containing 0.25 µg/ml nocodazole to prevent G2/M-phase cells recycling. 4 h post-release, G1-phase cells were treated with mock- or 4 Gy irradiation. Twelve and 18 h later, cells were then pulsed for 30 min with 10 µM BrdU, collected and fixed overnight in ice-cold 70% ethanol. DNA denaturation was performed using a solution of 0.2 mg/ml of pepsin (Sigma-Aldrich) in 2 M HCl. BrdU was detected using an anti-BrdU-FITC conjugated antibody (AbD Serotec) and a solution of PI/RNaseA (10 µg/ml and 0.1 mg/ml respectively) was used for total DNA staining. Cells were analysed in an Attune NxT flow cytometer (Life Technologies) and data processed using FlowJo software (Three Star Inc).

Statistical methods

Prism 6 software (GraphPad Software Inc.) was typically used for statistical analysis of datasets, with the exception of the RNAseq analyses that were performed using R (www.r-project.org).

CRISPR-Cas9 guide-RNAs (gRNAs) used to generate knockout and 53BP1$^{\Delta BRCT}$ cell lines.

Target gene/Name		Sequence (5'-3')	Description
TP53		gtgcagctgtgggttgattc	Targeted to exon 5, antisense.
53BP1	1	gaatccaactgacttccagt	Targeted to exon 2, antisense
	2	gctgagaatcttcaattatc	Targeted to exon 2, antisense
	3	gaacgaggagacggtaatagt	Targeted to exon 3, sense.
USP28	1	tgtagcaacagtgtcttgac	Targeted to exon 3, antisense.
	2	tgccattgctttgagtctac	Targeted to exon 4, sense.
53BP1	BRCT Nt 1	ctgtgagagtggagacaaca	Targeted to BRCT 1 N-term, antisense.
	BRCT Nt 2	tttgtgagcccctgtgagag	Targeted to BRCT 1 N-term, sense.
	BRCT Ct	ctcattgttggggagagaat	Targeted to BRCT 2 C-term, sense.

53BP1 lentiviral plasmids used throughout this study.

**Name**		**Description**	**Application**
pLenti-PGK-PURO-DEST	eGFP	Control plasmid	N3 survival studies
	GST	Control plasmid	
	53BP1	Full length 53BP1 ORF	
	53BP1$^{\Delta BRCT}$	Deletion of BRCT tandem domain	
	53BP1$^{\Delta OD}$	Deletion of Oligomerisation domain	
	53BP1^{ODm}	Mutation YYVD1258AAAA	
	53BP1^{20AQ}	Mutation of 20 N-terminal S/TQ sites	
	53BP1^{D1521R}	Mutation of the Tudor domain	
	53BP1^{L1619A}	Mutation of the UDR domain	
	53BP1^{R1811A}	Mutation in BRCT P-binding pocket	
	53BP1^{K1814M}	Mutation in BRCT P-binding pocket	
	53BP1^{N1845R}	Mutation in p53 binding interphase	
	53BP1^{N1845A}		
	53BP1^{D1861R}	Mutation in p53 binding interphase	
	53BP1^{D1861A}		
	USP28	Full length USP28 ORF	
	USP28$^{\Delta UBA}$	Deletion of predicted CUE domain (S20-E65)	
	USP28$^{\Delta UIM}$	Deletion of UIM domain (K99-I116)	
	USP28^{C171A}	Catalytic dead USP28 version	
pHAGE-N-FLAG-HA-DEST	eGFP	Control plasmid	Interaction studies (Co-IP)
	53BP1	Full length 53BP1 ORF	
	53BP1$^{\Delta BRCT}$	Deletion of BRCT tandem domain	
	53BP1^{ODm}	Mutation YYVD1258AAAA	
	53BP1^{D1521R}	Mutation of the Tudor domain	
	53BP1^{R1811A}	Mutation in BRCT P-binding pocket	
	53BP1^{K1814M}	Mutation in BRCT P-binding pocket	
	53BP1^{N1845R}	Mutation in p53 binding interphase	
	53BP1^{N1845A}		
	53BP1^{D1861R}	Mutation in p53 binding interphase	
	53BP1^{D1861A}		

Sequences of primer pairs used throughout this study.

Target gene / locus		Sequence (5'-3')	Application
CDKN1A (*p21*)	Fwd	CCTCATCCCGTGTTCTCCTTT	Transcript-specific qRT-PCR primers
	Rev	GTACCACCCAGCGGACAAGT	
BAX	Fwd	CCTTTTCTACTTTGCCAGCAAAC	
	Rev	GAGGCCGTCCCAACCAC	
BBC3 (*PUMA*)	Fwd	CCTGGAGGGTCCTGTACAATCT	
	Rev	GCACCTAATTGGGCTCCATCT	
TP53I3	Fwd	AGGGTGAAGTCCTCCTGAAGGT	
	Rev	GTGGGTCATACTGGCCTTGTCT	
MDM2	Fwd	GGCCTGCTTTACATGTGCAA	
	Rev	GCACAATCATTTGAATTGGTTGTC	
CDKN1A (*p21*) -2965	Fwd	CCGGCCAGTATATATTTTTAATTGAGA	Locus-specific ChIP primers
	Rev	AGTGGTTAGTAATTTTCAGTTTGCTCAT	
CDKN1A (*p21*) -2283	Fwd	AGCAGGCTGTGGCTCTGATT	
	Rev	CAAAATAGCCACCAGCCTCTTCT	
CDKN1A (*p21*) -1391	Fwd	CTGTCCTCCCCGAGGTCA	
	Rev	ACATCTCAGGCTGCTCAGAGTCT	
CDKN1A (*p21*) -20	Fwd	TATATCAGGGCCGCGCTG	
	Rev	GGCTCCACAAGGAACTGACTTC	
CDKN1A (*p21*) +507	Fwd	CCAGGAAGGGCGAGGAAA	
	Rev	GGGACCGATCCTAGACGAACTT	
CDKN1A (*p21*) +4001	Fwd	AGTCACTCAGCCCTGGAGTCAA	
	Rev	GGAGAGTGAGTTTGCCCATGA	
CDKN1A (*p21*) +8566	Fwd	CCTCCCACAATGCTGAATATACAG	
	Rev	AGTCACTAAGAATCATTTATTGAGCACC	
CDKN1A (*p21*) +11443	Fwd	TCTGTCTCGGCAGCTGACAT	
	Rev	ACCACAAAAGATCAAGGTGAGTGA	
MDM2 5′	Fwd	GGGCTATTTAAACCATGCATTTTC	
	Rev	GTCCGTGCCCACAGGTCTA	
MDM2 3′	Fwd	CTTTCTCGAGGAGGCAGGTTT	
	Rev	GCTCAACCCTAGGCGCTATTC	
GADD45A p53-RE	Fwd	GCCTTTGTCCGACTAGAGTGT	
	Rev	GGATCTCTTCCGCTGCTG	
FAS p53-RE	Fwd	GCACCGAAGCAGTGGTTAAG	
	Rev	GCCTCCAGAAGCTCATTCAG	

Antibodies used in this study.

Target	Manufacturer	Application
53BP1	Novus Biological (NB100-304)	WB, Immunofluorescence
	Novus Biological (NB100-305)	WB
	Millipore (clone B13)	WB, Immunofluorescence
MDM2	Santa Cruz Biotechnology (clone SMP14)	WB
p53	Dako (clone DO-7)	WB, Immunofluorescence
	Santa Cruz Biotechnology (clone DO-1)	Immunoprecipitation, ChIP
p21	BD Transduction Laboratories	WB
CHK2	Millipore (clone 7)	WB
$pCHK2^{T68}$	Cell signalling (clone C13C1)	WB
USP28	Abcam (EPR4249)	WB
HA-11	Covance Research (Clone 16B12)	WB
β-actin	Sigma-Aldrich	WB
α-tubulin	Sigma-Aldrich (Tat-1)	WB
RIF1	Bethyl Laboratories (A300-569A)	Immunofluorescence
ɣ-H2AX	Millipore	Immunofluorescence
Cyclin A	Clone E23.1, a gift from Julian Gannon, The Francis Crick Institute	Immunofluorescence

Supplementary References

Kim, D., Pertea, G., Trapnell, C., Pimentel, H., Kelley, R., Salzberg, S.L. (2013). TopHat2: accurate alignment of transcriptomes in the presence of insertions, deletions and gene fusions. Genome Biol *14*, R36.

Lamble, S., Batty, E., Attar, M., Buck, D., Bowden, R., Lunter, G., Crook, D., El-Fahmawi, B., Piazza, P. (2013). Improved workflows for high throughput library preparation using the transposome-based nextera system. BMC Biotech *13*, 104.

Lottersberger, F., Bothmer, A., Robbiani, D.F., Nussenzweig, M.C., and De Lange, T. (2013). Role of 53BP1 oligomerization in regulating double-strand break repair. Proc. Natl. Acad. Sci. U.S.A. *110*, 2146–2151.

Robinson, M.D., McCarthy, D.J., Smyth, G.K. (2010). edgeR: a Bioconductor package for differential expression analysis of digital gene expression data. Bioinformatics *26*, 139-140.
